# Accounting for epistasis improves genomic prediction of phenotypes with univariate and bivariate models across environments

**DOI:** 10.1007/s00122-021-03868-1

**Published:** 2021-06-11

**Authors:** Elaheh Vojgani, Torsten Pook, Johannes W. R. Martini, Armin C. Hölker, Manfred Mayer, Chris-Carolin Schön, Henner Simianer

**Affiliations:** 1 Center for Integrated Breeding Research, Animal Breeding and Genetics Group, University of Goettingen, Goettingen, Germany; 2 International Maize and Wheat Improvement Center (CIMMYT), Texcoco, State of Mexico Mexico; 3 Plant Breeding, TUM School of Life Sciences Weihenstephan, Technical University of Munich, Freising, Germany

## Abstract

**Key Message:**

**The accuracy of genomic prediction of phenotypes can be increased by including the top-ranked pairwise SNP interactions into the prediction model.**

**Abstract:**

We compared the predictive ability of various prediction models for a maize dataset derived from 910 doubled haploid lines from two European landraces (Kemater Landmais Gelb and Petkuser Ferdinand Rot), which were tested at six locations in Germany and Spain. The compared models were Genomic Best Linear Unbiased Prediction (GBLUP) as an additive model, Epistatic Random Regression BLUP (ERRBLUP) accounting for all pairwise SNP interactions, and selective Epistatic Random Regression BLUP (sERRBLUP) accounting for a selected subset of pairwise SNP interactions. These models have been compared in both univariate and bivariate statistical settings for predictions within and across environments. Our results indicate that modeling all pairwise SNP interactions into the univariate/bivariate model (ERRBLUP) is not superior in predictive ability to the respective additive model (GBLUP). However, incorporating only a selected subset of interactions with the highest effect variances in univariate/bivariate sERRBLUP can increase predictive ability significantly compared to the univariate/bivariate GBLUP. Overall, bivariate models consistently outperform univariate models in predictive ability. Across all studied traits, locations and landraces, the increase in prediction accuracy from univariate GBLUP to univariate sERRBLUP ranged from 5.9 to 112.4 percent, with an average increase of 47 percent. For bivariate models, the change ranged from −0.3 to + 27.9 percent comparing the bivariate sERRBLUP to the bivariate GBLUP, with an average increase of 11 percent. This considerable increase in predictive ability achieved by sERRBLUP may be of interest for “sparse testing” approaches in which only a subset of the lines/hybrids of interest is observed at each location.

**Supplementary Information:**

The online version contains supplementary material available at 10.1007/s00122-021-03868-1.

## Introduction

Genomic prediction of phenotypes has been widely explored for crops (Crossa et al. 2010), livestock(Daetwyler et al. 2013), and clinical research (de los Campos et al*.* 2013). Broad availability and cost-effective generation of genomic data had a considerable impact on plant (Bernardo and Yu 2007; de los Campos et al. 2009; Crossa et al. 2010, 2011; de Los Campos et al. 2010; Pérez et al. 2010) and animal breeding programs (de los Campos et al*.* 2009; Hayes and Goddard 2010; Daetwyler et al. 2013). Genomic prediction relates a set of genome-wide markers to the variability in the observed phenotypes and enables the prediction of phenotypes or genetic values of genotyped but unobserved material (Meuwissen et al. 2001; Jones 2012; Windhausen et al. 2012). This approach has been positively evaluated in most major crop and livestock species (Albrecht et al. 2011; Daetwyler et al. 2013; Desta and Ortiz 2014) and is becoming a routine tool in commercial and public breeding programs (Stich and Ingheland 2018). In plant breeding, phenotyping is one of the major current bottlenecks and the optimization or minimization of phenotyping costs within breeding programs is needed (Akdemir and Isidro-Sánchez 2019). Therefore, the maximization of genomic prediction accuracy can be directly translated into reduced phenotyping costs (Akdemir and Isidro-Sánchez 2019; Jarquin et al. 2020).

Genomic selection and the corresponding prediction of breeding values are based on a covariance matrix describing the (additive) relationship between the considered individuals (Wolc et al. 2011; Burgueño et al. 2012). This matrix can be constructed from pedigree information, from marker information (VanRaden 2007), or from a combination of pedigree and available genotypic information in a single step approach (Aguilar et al. 2010; Legarra et al. 2014). It has been broadly demonstrated that marker-based relationship matrices enhance the reliability of breeding value estimation on average across traits and compared to pedigree-based approaches (Meuwissen et al. 2001; VanRaden 2007; Hayes and Goddard 2008; Crossa et al. 2010). Since breeding values are additive by definition (Falconer and Mackay 1996), the early development of prediction models exclusively accounted for the additive effects (Filho et al. 2016).

Concerning additive models, genomic best linear unbiased prediction (GBLUP, Meuwissen et al. 2001; VanRaden 2007) is a widely used linear mixed model (Da et al. 2014; Rönnegård and Shen 2016; Covarrubias-Pazaran et al. 2018). Although various new approaches such as methods from the Bayesian alphabet (Gianola et al. 2009) have been proposed, GBLUP remains the gold standard as new methods typically only perform marginally better, are less robust, require substantially more computing time, and are more difficult to implement (Wang et al. 2018). Daetwyler et al. (2010) showed that BayesB can yield higher accuracy than GBLUP for traits controlled by a small number of quantitative trait nucleotides, emphasizing that the genetic architecture of the trait has an important impact on which method may predict better (Wimmer et al. 2013; Momen et al. 2018). Moreover, the training set size was shown to play a role. For instance, human height prediction using BayesB and BayesC methods in a small reference population (< 6000 individuals) had no advantage over GBLUP. Only when increasing the size of the reference population (> 6000 individuals), these methods outperformed GBLUP (Karaman et al. 2016).

Understanding how genetic variation causes phenotypic variation in quantitative traits is still a major challenge of contemporary biology. It has been proved that epistasis as a statistical interaction between two or more loci (Falconer and Mackay 1996) contributes substantially to the genetic variation in quantitative traits (Wright 1931; Carlborg and Haley 2004; Hill et al. 2008; Huang et al. 2012; Mackay 2014). On the one hand, models which incorporate epistasis have the potential to increase predictive ability (de Los Campos et al. 2010; Hu et al. 2011; Wang et al. 2012; Mackay 2014). On the other hand, accounting for epistasis by modeling interactions explicitly was considered to be computationally challenging (Mackay 2014). In this context, the extended genomic best linear unbiased prediction (EG-BLUP), as an epistasis marker effect model (Jiang and Reif 2015; Martini et al. 2016), and reproducing kernel Hilbert space regression (RKHS), as a semi-parametric model (Gianola et al. 2006; Gianola and van Kaam 2008; de Los Campos et al. 2010) based on Gaussian kernel (Jiang and Reif 2015), were proposed to reduce the computational load by constructing marker-based epistatic relationship matrices (Jiang and Reif 2015; Martini et al. 2016). RKHS has shown to be as good as (Jiang and Reif 2015) or better than EG-BLUP (Martini et al. 2017). While EG-BLUP is potentially beneficial for genomic prediction, its performance depends on the marker coding (Martini et al. 2017, 2019). Moreover, it has been shown that the superiority of epistasis models over the additive GBLUP in terms of predictive ability may vanish when the number of markers increases (Schrauf et al. 2020). Also, the Hadamard products of the additive genomic relationship matrices provide only an approximation for the interaction effect model based on interactions between different loci (Martini et al. 2020), and more correcting factors are required for interactions of higher degree (Jiang and Reif 2020).

Another downside of epistasis models is that, due to the high number of interactions, a large number of unimportant variables can be introduced into the model (Rönnegård and Shen 2016). This ‘noise’ might prevent a gain in predictive ability. In this regard, Martini et al. (2016) showed that selecting just a subset of the largest epistatic interaction effects has the potential to improve predictive ability. Therefore, reducing the full epistasis model to a model based on a subnetwork of ‘most relevant’ pairwise SNP interactions may be beneficial for prediction performance (Martini et al. 2016).

In addition to the extension from additive effect models to models including epistatic interactions, genomic prediction models can be extended from univariate models to multivariate models. Univariate models consider each trait separately, while multivariate models treat several traits simultaneously with the objective to exploit the genetic correlation between them to increase predictive ability. Multivariate models which have been first proposed for the prediction of genetic values by Henderson and Quaas (1976) were shown to be potentially beneficial for prediction accuracy when the correlation between traits is strong (He et al. 2016; Covarrubias-Pazaran et al. 2018; Schulthess et al. 2018; Velazco et al. 2019). A situation of dealing with multiple environments can also be considered in the framework of a multivariate model by simply considering a trait-in-environment combination as another correlated trait. This is considered as the multi-environment model which is usually employed to assess $G \times E$ interaction (Montesinos-López et al. 2016; Ben et al. 2018) and captures the differences in genotypes’ performances from one environment to the other as one of the breeders’ major challenges in plant breeding (Kang and Gorman 1989). Prediction accuracy could be potentially enhanced through borrowing information across environments by utilizing multi-environment models (Burgueño et al. 2012). In addition to multi-environment models, Martini et al. (2016) showed that the predictive ability of EG-BLUP as a univariate model can be increased in one environment by variable selection in the other environment under the assumption of a relevant correlation of phenotypes in different environments. This, however, was only demonstrated with a data set of limited size, and especially a limited set of markers, and, thus, marker interactions.

In the present study, we use a data set of doubled haploid lines derived from two European landraces, to investigate how beneficial the use of subnetworks of interactions in the proposed sERRBLUP framework can be. This was compared in the context of univariate and bivariate models. We assess the optimum proportion of SNP interactions to be kept in the model in the variable selection step. The development of efficient selection strategies which could mitigate costly and time consuming phenotyping of a large number of selection candidates in multiple environments has been a particular focus of research in plant breeding (Jarquin et al. 2020). A successful application of our models may reduce the cost of phenotyping by reducing the number of test locations per line.

## Materials and methods

### Data used for analysis

We used a set of 501/409 doubled haploid lines of the European maize landraces Kemater Landmais Gelb/Petkuser Ferdinand Rot genotyped with 501,124 markers using the Affymetrix ® Axiom Maize Genotyping Array (Unterseer et al. 2014), out of which 471 and 402 lines were phenotyped for Kemater (KE) and Petkuser (PE), respectively. The performance of the lines has been evaluated by ten separate 10 × 10 lattice designs in four German locations and five separate 10 × 10 lattice designs in two Spanish locations with two replicates. For more details, see Hölker et al. (2019).

The lines were phenotyped in 2017 for a series of traits in six different environments which were Bernburg (BBG, Germany), Einbeck (EIN, Germany), Oberer Lindenhof (OLI, Germany), Roggenstein (ROG, Germany), Golada (GOL, Spain), and Tomeza (TOM, Spain).

The descriptions of the phenotypic traits, comprising early vigor and mean plant height of three plants of the plot at three growth stages (EV_V3, EV_V4, EV_V6, PH_V4, PH_V6, PH_final), days from sowing until female flowering (FF), and root lodging (RL), are given in the supplementary (Table S1), together with the number of phenotyped lines per location, phenotypic means, standard deviations, and maximum and minimum values. To correct for spatial structure and population effects, Best Linear Unbiased Estimations (BLUEs) were used as input for all considered prediction models. The interested reader is referred to Hölker et al. (2019) for details on the correction procedure and the detailed description of the considered traits, e.g., the trait “growth stage V4” indicates the growth stage at which four leaf collars are fully developed (Abendroth et al. 2011). In our study, we chose PH_V4 as the main trait for evaluating and illustrating our methods, since it is a relevant metric quantitative trait for early plant development which is suitable for testing our methods. The phenotypic correlations of PH_V4 across all environments are provided in Table 1.

**Table 1:** Phenotypic correlation across all environments for the trait PH_V4 in KE (*italic* numbers above diagonal) and PE (**bold** numbers below diagonal) which are highly significant (*p*_values < 0.001)

Location	BBG	EIN	OLI	ROG	GOL	TOM
BBG	–	*0.82*	*0.66*	*0.67*	*0.69*	*0.58*
EIN	**0.78**	–	*0.71*	*0.77*	*0.75*	*0.66*
OLI	**0.60**	**0.66**	–	*0.71*	*0.65*	*0.50*
ROG	**0.62**	**0.69**	**0.65**	–	*0.70*	*0.58*
GOL	**0.55**	**0.59**	**0.46**	**0.51**	–	*0.69*
TOM	**0.57**	**0.68**	**0.57**	**0.58**	**0.54**	–

Among the phenotypic traits, root lodging (RL) and female flowering (FF) were not phenotyped in all the environments: RL was only scored in BBG, ROG, OLI, and EIN, and FF was phenotyped in all environments except GOL.

### Quality control, coding, and imputing

As we would not expect any heterozygous calls in DH material, all heterozygous calls were set to missing. Genotype calls were coded according to the allele counts of the B73 AGPv4 reference sequence (Jiao et al. 2017) (0 = homozygous for the reference allele, 2 = homozygous for the alternative allele). Imputation of missing values was performed separately for each landrace, using BEAGLE version 4.0 with parameters buildwindow = 50, nsamples = 50 (Browning and Browning 2007; Pook et al. 2020). For the remaining heterozygous calls, the DS (dosage) information of the BEAGLE output was used and genotyped with DS < 1 being set to 0 and DS >  = 1–2.

### Linkage disequilibrium pruning

Linkage disequilibrium-based SNP pruning with PLINK v1.07 was used to generate a subset of SNPs which are in approximate linkage equilibrium with each other. The parameters: indep 50 5 2 were used, in which 50 is the window size in SNPs, 5 is the number of SNPs to shift the window at each step, and 2 is the variance inflation factor ${\text{VIF}} = 1/\left( {1 - r^{2} } \right)$, where $r^{2}$ is the squared correlation between single SNPs and linear combinations of all SNPs in the window. All variants in the 50 SNP window which had a VIF > 2 were removed. Then, the window was shifted 5 SNPs forward, and the procedure was repeated (Purcell et al. 2007; Chang et al. 2015).

In our study, LD pruning was carried out separately for each landrace, resulting in data panels containing 25′437 SNPs for KE and 30′212 SNPs for PE.

### Univariate statistical models for phenotype prediction

We used three different statistical models to predict phenotypes, which are all based on a linear mixed model (Henderson 1975). We assume that we have in total $n$ lines which are genotyped, and phenotypes are available for a subset of $n_{1}$ lines. These $n_{1}$ lines are used to train the model, and missing phenotypes for the remaining $n_{2} = n - n_{1}$ lines are predicted by using the genotypes of these lines. The basic univariate model is

$$y = 1\mu + \left( {\begin{array}{*{20}c} I & O \\ \end{array} } \right)g + \in$$

where $y$ is an $n_{1} \times 1$ vector of phenotypes, $1$ is an $n_{1} \times 1$ vector with all entries equal to 1, $\mu$ is a scalar fixed effect, $I$ is an identity matrix of dimension $n_{1} \times n_{1}$, and $O$ is a matrix of dimension $n_{1} \times n_{2}$ of zeros. The design matrix $$\left( {\begin{array}{*{20}c} I & O \\ \end{array} } \right)$$ is the $n_{1} \times (n_{1} + n_{2} )$ matrix resulting from the concatenation of $I$ and $O$. Moreover, $gN \to \left( {0, \Gamma \sigma_{g}^{2} } \right)$ is an $n \times 1$ vector of random genetic effects, and $\in \to \left( {0,I\sigma_{ \in }^{2} } \right)$ is a random error vector, where $\Gamma$ and $I$ are the respective dispersion matrices and $\sigma_{g}^{2}$ and $\sigma_{\varepsilon }^{2}$ are the corresponding variance components.

With this model, the population mean and the genetic effects $g$ for all lines, including those without phenotypes, are estimated using

1 $$\left[ {\begin{array}{*{20}c} {\hat{\mu }} \\ {\begin{array}{*{20}c} {\hat{g}_{1} } \\ {\hat{g}_{2} } \\ \end{array} } \\ \end{array} } \right] = \left[ {\begin{array}{*{20}c} {n_{1} } & {1^{\prime}} & 0 \\ 1 & {I + \lambda \Gamma^{11} } & {\lambda \Gamma^{12} } \\ 0 & {\lambda \Gamma^{21} } & {\lambda \Gamma^{22} } \\ \end{array} } \right]^{ - 1} \left[ {\begin{array}{*{20}c} {1^{\prime}y} \\ {\begin{array}{*{20}c} y \\ 0 \\ \end{array} } \\ \end{array} } \right]$$

where $\lambda = \sigma_{\varepsilon }^{2} /\sigma_{g}^{2}$, $$\Gamma^{ - 1} = \left[ {\begin{array}{*{20}c} {\Gamma^{11} } & {\Gamma^{12} } \\ {\Gamma^{21} } & {\Gamma^{22} } \\ \end{array} } \right]$$ and $$g = \left[ {\begin{array}{*{20}c} {g_{1} } \\ {g_{2} } \\ \end{array} } \right]$$ and the indices pertain to the subset of individuals with (index 1) or without (index 2) phenotypes, respectively.

With these estimates, the phenotypes for the set of unphenotyped individuals can be predicted as $\hat{y}_{2} = 1_{2} \hat{\mu } + \hat{g}_{2}$, where $\hat{y}_{2}$ is the $n_{2} \times 1$ vector of predicted phenotypes and $1_{2}$ is an $n_{2} \times 1$ vector of ones.

For $n = n_{1}$ and $n_{2} = 0$, the solution of Eq. (1) provides estimates of genetic effects when all lines are phenotyped and genotyped.

### Bivariate statistical models for phenotype prediction

Besides univariate models, we also used bivariate models, where the two variables represent the same trait measured in two different environments.

The basic bivariate model is

$$y = X\mu + Zg + e$$

or, in more detail,

2 $$\left[ {\begin{array}{*{20}c} {y_{1} } \\ {y_{2} } \\ \end{array} } \right] = \left[ {\begin{array}{*{20}c} {1_{1} } & 0 \\ 0 & {1_{2} } \\ \end{array} } \right]\left[ {\begin{array}{*{20}c} {\mu_{1} } \\ {\mu_{2} } \\ \end{array} } \right] + \left[ {\begin{array}{*{20}c} {I_{1} } & 0 \\ 0 & {I_{2} } \\ \end{array} } \right]\left[ {\begin{array}{*{20}c} {g_{1} } \\ {g_{2} } \\ \end{array} } \right] + \left[ {\begin{array}{*{20}c} {e_{1} } \\ {e_{2} } \\ \end{array} } \right]$$

where $$\left[ {\begin{array}{*{20}c} {y_{1} } \\ {y_{2} } \\ \end{array} } \right]$$ is the phenotype vector of length $m$ $= m_{1} + m_{2}$ for environment 1 ($m_{1}$) and 2 ($m_{2}$), $1_{1}$ and $1_{2}$ are, respectively, $m_{1} \times 1$ and $m_{2} \times 1$ vectors with all entries equal to 1, $$\left[ {\begin{array}{*{20}c} {\mu_{1} } \\ {\mu_{2} } \\ \end{array} } \right]$$ is the vector of population means for environments 1 and 2, $I_{1}$ and $I_{2}$ are identity matrices of dimension $m_{1} \times m_{1}$ and $m_{2} \times m_{2}$, respectively, assigning genomic values to phenotypes. Moreover, $$\left[ {\begin{array}{*{20}c} {g_{1} } \\ {g_{2} } \\ \end{array} } \right]$$ is the vector of random genomic values which is assumed to have a multivariate normal distribution with mean zero and variance $G = H \otimes \Gamma$, where $$H = \left[ {\begin{array}{*{20}c} {\sigma_{{g_{1} }}^{2} } & {\sigma_{{g_{12} }} } \\ {\sigma_{{g_{12} }} } & {\sigma_{{g_{2} }}^{2} } \\ \end{array} } \right]$$, $\Gamma$ is the dispersion matrix of genetic effects, and $\otimes$ is the Kronecker product. $$\left[ {\begin{array}{*{20}c} {{\varvec{e}}_{1} } \\ {{\varvec{e}}_{2} } \\ \end{array} } \right]$$ is the vector of random errors which is assumed to have a multivariate normal distribution with mean zero and variance $R = R_{0} \otimes I$**,** where $$R_{0} = \left[ {\begin{array}{*{20}c} {\sigma_{{e_{1} }}^{2} } & {\sigma_{{e_{12} }} } \\ {\sigma_{{e_{12} }} } & {\sigma_{{e_{2} }}^{2} } \\ \end{array} } \right]$$. $\sigma_{{g_{i} }}^{2}$ and $\sigma_{{e_{i} }}^{2}$ represent the genetic and residual variance of environment $i = 1,2$, and $\sigma_{{g_{12} }}$ and $\sigma_{{e_{12} }}$ are the genetic and residual covariance between the environments 1 and 2 (Guo et al. 2014). In this model, the phenotypes have to be ordered in the same way in both environments. In case the number of observations in environment 1 and environment 2 is not identical (i.e., in general terms $m_{1} \ne m_{2}$) or different lines are considered in the model, the incidence matrices have to be adapted accordingly.

With this model, the vector of environment specific population means and the vector of genetic effects for all lines are estimated using the standard mixed model equations

$$\left[ {\begin{array}{*{20}c} {\hat{\mu }} \\ {\hat{g}} \\ \end{array} } \right] = \left[ {\begin{array}{*{20}c} {X^{\prime}R^{ - 1} X} & {X^{\prime}R^{ - 1} Z} \\ {Z^{\prime}R^{ - 1} X} & {Z^{\prime}R^{ - 1} Z + G^{ - 1} } \\ \end{array} } \right]^{ - 1} \left[ {\begin{array}{*{20}c} {X^{\prime}R^{ - 1} y} \\ {Z^{\prime}R^{ - 1} y} \\ \end{array} } \right]$$

In analogy to the procedure described in the univariate setting, we consider a setting in which the last $l$ phenotypes for environment 2 are masked and predicted from all observations in environment 1 and the first $k = m_{2} - l$ non-masked observations in environment 2.

$$\left[ {\begin{array}{*{20}c} {y_{1} } \\ {\begin{array}{*{20}c} {y_{k} } \\ 0 \\ \end{array} } \\ \end{array} } \right] = \left[ {\begin{array}{*{20}c} {1_{1} } & 0 \\ {\begin{array}{*{20}c} 0 \\ 0 \\ \end{array} } & {\begin{array}{*{20}c} {1_{k} } \\ 0 \\ \end{array} } \\ \end{array} } \right]\left[ {\begin{array}{*{20}c} {\mu_{1} } \\ {\mu_{2} } \\ \end{array} } \right] + \left[ {\begin{array}{*{20}c} {I_{1} } & 0 & 0 \\ 0 & {I_{k} } & 0 \\ 0 & 0 & {I_{l} } \\ \end{array} } \right]\left[ {\begin{array}{*{20}c} {g_{1} } \\ {\begin{array}{*{20}c} {g_{2k} } \\ {g_{2l} } \\ \end{array} } \\ \end{array} } \right] + \left[ {\begin{array}{*{20}c} {e_{1} } \\ {\begin{array}{*{20}c} {e_{k} } \\ 0 \\ \end{array} } \\ \end{array} } \right]$$

From the solutions obtained with this model, the phenotypes for the set of unphenotyped individuals in environment 2 can be predicted as $\hat{y}_{l} = 1_{l} \hat{\mu }_{2} + \hat{g}_{2l}$, where $\hat{y}_{l}$ is the $l \times 1$ vector of predicted phenotypes and $1_{{\varvec{l}}}$ is an $l \times 1$ vector of ones.

The three models compared in this study only differ in the choice of the dispersion matrix $\Gamma$ of the genetic effects.

#### Model 1: genomic best linear unbiased prediction (GBLUP)

In this additive model, we use as $\Gamma$ the genomic relationship matrix which is calculated according to VanRaden (2008) as

$$\Gamma_{{{\text{VR}}}} = \frac{{\left( {M - P} \right)\left( {M - P} \right)^{^{\prime}} }}{{2 \cdot \mathop \sum \nolimits_{i = 1}^{m} \left( {p_{i} \left( {1 - p_{i} } \right)} \right)}}$$

where $$M$$ is the $$n \times m$$ marker matrix which gives $$m$$ marker values for $$n$$ lines under the assumption of having $$n$$ genotyped lines in total. $$P$$ is a matrix of equal dimension as $$M$$ with $$2$$$$\cdot$$ in the $$i{\text{th}}$$ column, and $$p_{i}$$ is the allele frequency of the minor allele of SNP$$i$$.

#### Model 2: Epistatic Random Regression BLUP (ERRBLUP)

This model accounts for all possible SNP interactions in the prediction model. With m markers and fully inbred lines, we have two possible genotypes at a single locus, i.e., 0 or 2 when coded as the counts of the minor allele. For each pair of loci, we have four different possible genotype combinations: {00, 02, 20, 22}. The total number of pairs of loci is $\frac{{m \times \left( {m + 1} \right)}}{2}$ if we allow for interaction of a locus with itself. Since for each of these pairs we have four possible genotype combinations, the total number of combinations to be considered as dummy variables is:

$$m^{*} = 4 \times \frac{{m \times \left( {m + 1} \right)}}{2} = 2m \times \left( {m + 1} \right)$$.

We define a $$i$$ marker combination matrix $$M^{*}$$ of dimension $$n \times m^{*}$$ whose element $$i$$$$,$$ is 1 if genotype combination $$j$$ is present in individual $$i$$ and is 0 otherwise. We further define for column of this matrix the average value $$p_{i}^{*}$$, giving the frequency of the respective genotype combination in the population, and a matrix $$P^{*}$$ being of equal dimension as $${\varvec{M}}^{*}$$ with $$p_{i}^{*}$$ in the $$i{\text{th}}$$ column.

Then, the relationship matrix based on all SNP interactions was calculated according to VanRaden (2008) as

$$\Gamma_{{{\text{ERR}}}} = \frac{{\left( {M^{*} - P^{*} } \right)\left( {M^{*} - P^{*} } \right)^{^{\prime}} }}{{\mathop \sum \nolimits_{i = 1}^{{m^{*} }} \left( {p_{i}^{*} \left( {1 - p_{i}^{*} } \right)} \right)}}$$

and this matrix was used in ERRBLUP as dispersion matrix for the genetic effects, which now are based on epistatic interaction effects. It should be noted that including the interaction of each locus with itself replaces the additive effect, so that it is not necessary to use a model that separately accounts for additive and epistatic effects. This model had been introduced earlier as “categorical epistasis model” (Martini et al. 2017).

#### Model 3: Selective Epistatic Random Regression BLUP (sERRBLUP)

sERRBLUP is based on the same approach as ERRBLUP, but here the $\Gamma$ -matrix is constructed from a selected subset of genotype interactions. We decided to use those interactions with the highest estimated marker effects variances. Selection based on highest absolute effects (as used by Martini et al. (2016) in the framework of the EGBLUP epistasis model) was also considered, but leads to similar to slightly worse results. For this, it was necessary to backsolve interaction effects $\hat{t}$ and effects variances $\mathop {\hat{\sigma }}\nolimits^{2}$from the ERRBLUP model using (Mrode 2014).

$$\hat{t} = \frac{{\mathop {\hat{\sigma }_{g}^{*} }\nolimits^{2} }}{{\mathop \sum \nolimits_{i = 1}^{{m^{*} }} \left( {p_{i}^{*} \left( {1 - p_{i}^{*} } \right)} \right)}}\left( {M^{*} - P^{*} } \right)^{^{\prime}} \left( {\mathop {\hat{\sigma }_{g}^{*} }\nolimits^{2} \Gamma_{{{\text{ERR}}}} + \mathop {\hat{\sigma }_{ \in }^{*} }\nolimits^{2} I} \right)^{ - 1} \left( {y - 1\hat{\mu }} \right)$$

$$\mathop {\hat{\sigma }}\nolimits^{2} = \left( {\hat{t} \circ \hat{t}} \right)2P^{*} \left( {1 - P^{*} } \right)$$

with $\circ$ denoting the Hadamard product.

After estimating SNP interaction effects in $\hat{t}$ and effects variances in $\hat{\sigma }^{2}$, we selected those interactions whose absolute estimated effects or effect variances were in the top $\pi = 0.05, 0.01, 0.001, 0.0001,{ }0.00001{\text{ or}} 0.000001$ proportion of all interactions, respectively. These proportions were chosen since it was observed in preliminary analyses that they cover the most relevant range. For each of these subsets, we generated reduced matrices $M_{\pi }^{*}$ and $P_{\pi }^{*}$ of dimension $n \times \pi m^{*}$, containing only those columns of $M^{*}$ and $P^{*}$ pertaining to the selected subset of genotype interactions, and then set up the dispersion matrix in analogy to VanRaden (2008) as

$$\Gamma_{{{\text{sERR}}}} = \frac{{\left( {M_{\pi }^{*} - P_{\pi }^{*} } \right)\left( {M_{\pi }^{*} - P_{\pi }^{*} } \right)^{^{\prime}} }}{{\mathop \sum \nolimits_{i = 1}^{{\pi m^{*} }} \left( {p_{\pi i}^{*} \left( {1 - p_{\pi i}^{*} } \right)} \right)}}$$

where $p_{\pi i}^{*}$ are the mean frequencies of the selected genotype combinations.

Note here that even for the univariate model, information from another environment is used for the prediction, namely for variable selection and the definition of $\Gamma_{{{\text{sERR}}}}$. However, having used the information from another environment to define the subset of interactions and to derive the relationship matrix $\Gamma_{{{\text{sERR}}}}$, the actual prediction is within the considered environment from the training to the test set.

We used the miraculix package (Schlather 2020) to efficiently calculate $\Gamma_{{{\text{ERR}}}}$, $\hat{t}$ and $\Gamma_{{{\text{sERR}}}}$.

### *Assessment of predictive ability *via* fivefold random cross-validation with 5 replicates*

In a fivefold cross-validation, the original sample is randomly partitioned into five subsamples of equal size. Out of the five subsamples, each subsample is subsequently considered as the test set for validating the model, and the remaining four subsamples are considered as training data. The training set is used to predict the test set. By this, all observations are used for both training and testing and each observation is only used once for testing (Utz et al. 2000). We repeated the cross-validation procedure 5 times, using random partitions of the original sample. The results of the 25 repetitions were then averaged (Erbe et al. 2010). We used the Pearson correlation between the predicted genetic value and the observed phenotype in the test set as the measure for predictive ability. In our study, predictive ability was assessed for PE and KE for all phenotypic traits separately. In addition, the trait’s prediction accuracy was calculated by dividing the obtained predictive ability by the square root of the respective trait’s heritability (Dekkers 2007). The numbers of KE and PE lines which are available for all combinations of environments are summarized in Table 2. For some traits, these numbers can be smaller or even zero for some environment combinations. We evaluated our univariate and bivariate models as follows:

**Table 2:** Number of KE (*italic* numbers above diagonal) and PE (**bold** numbers below diagonal) phenotyped lines in each pair of environments for trait PH_V4

Location	BBG	EIN	OLI	ROG	GOL	TOM
BBG	**393**/*461*	*461*	*441*	*461*	*200*	*200*
EIN	**393**	**393**/*462*	*441*	*461*	*201*	*201*
OLI	**390**	**390**	**390**/*441*	*441*	*182*	*181*
ROG	**390**	**390**	**389**	**390**/*461*	*200*	*200*
GOL	**195**	**195**	**195**	**195**	**204**/*211*	*209*
TOM	**195**	**195**	**195**	**195**	**204**	**204**/*210*

### Assessment of GBLUP, ERRBLUP, and sERRBLUP predictive abilities

The univariate GBLUP and ERRBLUP within environments were evaluated by training the model in the same environment as the test set was sampled from.

**Fig. 1:**
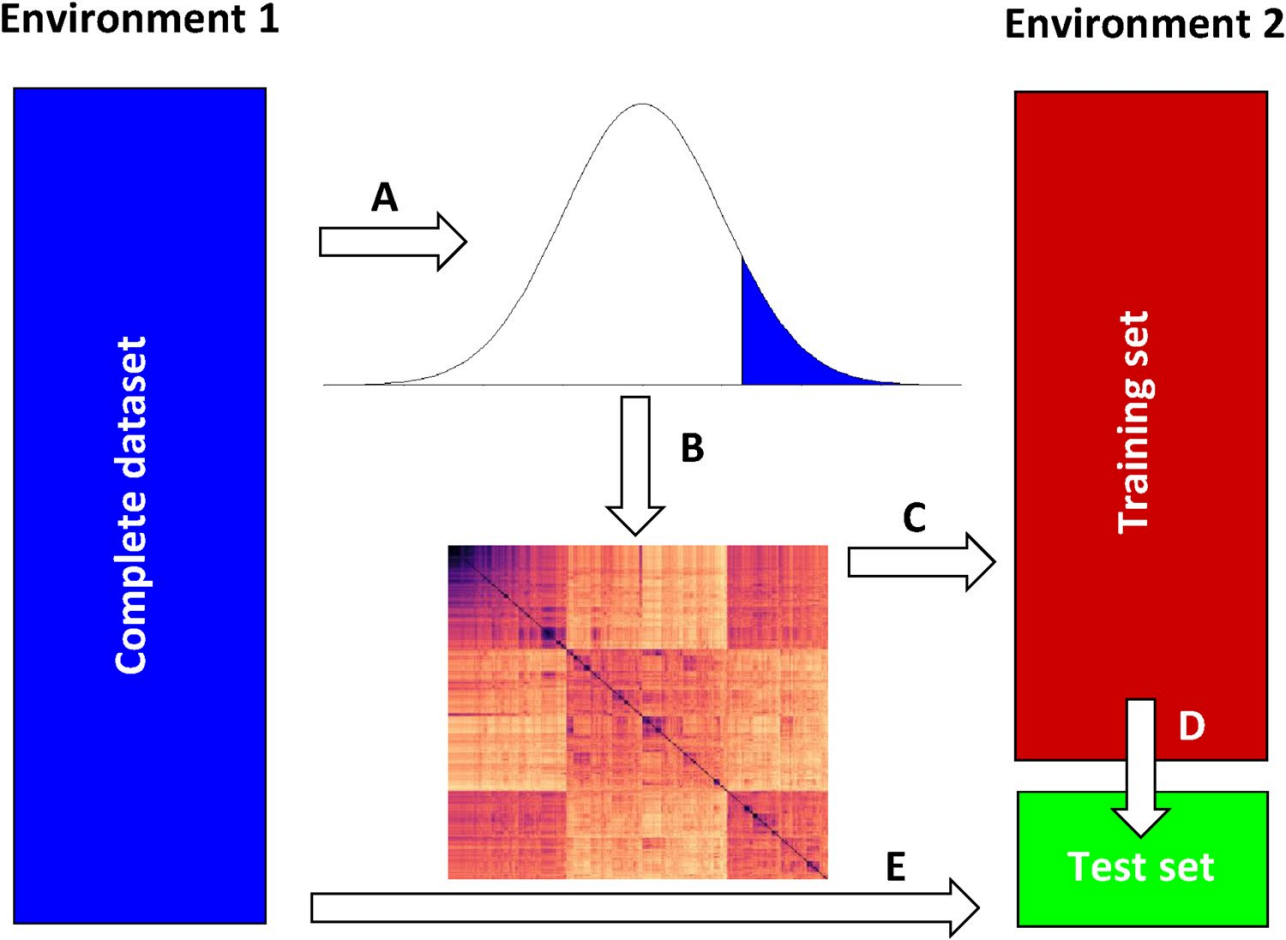
Basic scheme of uni- and bivariate sERRBLUP across environments. All pairwise SNP interaction effects and their variances are estimated from all data in environment 1, and effects are ordered either by absolute effect size or effect variance (**A**). Then, an epistatic relationship matrix for all lines is constructed from the top ranked subset of interaction effects (**B**) which in the univariate model is used in environment 2 (**C**) to predict phenotypes of the test set (green) from the respective training set (red, **D**). In the bivariate model, this information is combined with the complete data from environment 1 (blue, **E**) to predict the test set

The basic strategy for univariate and bivariate sERRBLUP across environments is illustrated in Fig. 1: first, all pairwise SNP interaction effects and their variances are estimated from all data in environment 1 and effects are ordered either by absolute effect size or by effect variance (A). Next, an epistatic relationship matrix for all lines is constructed from the top ranked subset of interaction effects (*B*). Then, this matrix is used in environment 2 (*C*) to predict phenotypes of the test set (green) from the respective training set (red) (*D*). This approach henceforth is termed ‘sERRBLUP across environments.’ In the case of bivariate sERRBLUP, both the full data panel from environment 1 and the training set from environment 2 are used in a bivariate prediction model.

The basic strategy for bivariate GBLUP and ERRBLUP is illustrated in Fig. 1 when the model is trained jointly on the complete dataset of environment 1 (*E*) and the training set of environment 2 (*D*). The test set of environment 2 is then predicted, using as dispersion matrix for the genetic effects either $\Gamma_{{{\text{VR}}}}$ or $\Gamma_{{{\text{ERR}}}}$.

### Use of multiple environments jointly

In addition to considering each environment separately, we used the average of all environments, except the current target environment, as an additional environment. This was considered for univariate sEERBLUP and all bivariate models.

### Estimation of variance and covariance components

Since we aimed at estimating variance components in each replicate of the cross-validation from the training data, variance component estimation with ASREML has a certain risk of non-convergence, in particular in models with a high number of parameters such as the models proposed here. Therefore, we needed to specify a strategy to deal with such cases in an automated manner. In univariate analyses, variance components were estimated using EMMREML (Akdemir and Godfrey 2015) in each run of a fivefold cross-validation based on the training set. In bivariate analyses, the variance components were estimated using ASReml-R (Butler et al. 2018). In the bivariate ERRBLUP and sERRBLUP models, the genetic and residual variance and covariance were estimated first from the full data set in a bivariate ASReml-R model for each combination of environments in each trait. If the estimation of variance components didn’t converge after 100 iterations, then the computation was stopped and the genetic and residual variance and covariance estimates at the last iteration (100) were extracted. These estimates were defined as the initial starting values of the bivariate ASReml-R model in each run of a fivefold cross-validation, followed by a re-estimation of the variance and covariance components based on the training set in the cross-validation. If the estimation of variance components did not converge at 50 iterations in each fold, the pre-estimated variance and covariance components based on the full dataset, which was defined as the initial start values of the model, were used as fixed values, so that the breeding values were estimated based on these pre-estimated parameters. It was verified from converged estimates that variance and covariance components estimated from the training set deviated only little from the variances and covariances from the full set (see Fig. S1). Also, the mean result obtained from just the converged replicates and the mean results of all replicates including the ones where variance and covariance components were fixed were rather similar (Fig. S2); only when the majority (> 20) of replicates failed to converge, substantial random fluctuation was observed. Thus, we argue that this strategy appears justifiable, but still the number of cases where estimates did not converge in fivefold cross-validation with 5 replicates and the combinations whose pre-estimation of variance components also did not converge in 100 iterations are detailed in the supplementary (Table S2–S9).

## Results

**Fig. 2:**
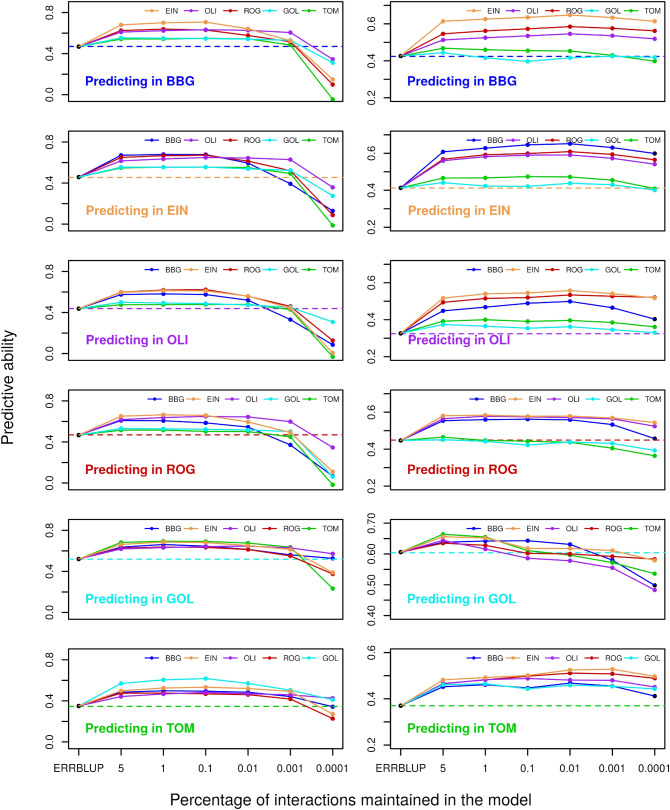
Predictive ability for univariate GBLUP within environment (dashed horizontal line), univariate ERRBLUP within environment (black filled circle), and univariate sERRBLUP across environments (solid colored lines) when SNP interaction selections are based on estimated effects variances in KE (left side) and PE (right side) for trait PH_V4. In each panel, the solid lines’ color indicates the environment in which the relationship matrices were determined by variable selection

Predictive abilities of univariate sERRBLUP across environments compared to univariate ERRBLUP and univariate GBLUP within environments for the trait PH_V4 are shown in Fig. 2 for KE and PE. Univariate GBLUP within the environment is used as a reference and is compared to results obtained with univariate ERRBLUP within environments and univariate sERRBLUP when the top 5, 1, 0.1, 0.01, 0.001, and 0.0001 percent of pairwise SNP interactions are maintained in the model. Figure 2 shows that the predictive abilities of univariate GBLUP and univariate ERRBLUP within the environment are almost identical (the highest deviation observed was 0.004). A considerable increase in predictive ability was observed when the top 1 or 0.1 percent of SNP interactions, selected based on their effect variances, were kept in the univariate sERRBLUP model. A more stringent selection, i.e., by considering only the top 0.01, 0.001, and 0.0001 percent of SNP interactions in the model, often led to a reduction in predictive ability, such that for the most stringent selection of 0.001 and 0.0001 percent, the predictive ability was sometimes even below the univariate GBLUP reference. This pattern is observed across all environments and is more pronounced in KE than PE. Results for the other traits are given in the Supplementary (Fig. S3a–S9a). In this study, estimated effect variances were identified as the best selection criteria in sERRBLUP, since sERRBLUP predictive abilities were observed to be more robust when the selection of pairwise SNP interaction was based on the effect variances compared to absolute effect sizes, especially when the top 0.001 and 0.0001 percent of interactions are maintained in the model (Fig. S10 and S11). In addition, the maximum predictive ability obtained from univariate sERRBLUP is almost identical when selecting SNP interactions based on absolute effect sizes or effect variances for both KE and PE (Fig. S12).

In the context of univariate models, we also investigated the predictive ability of univariate sERRBLUP when the variable selection was based on the training set from the same environment as the test set. This was exemplarily done within Bernburg for the trait PH_V4 (Fig. S13), illustrating that the predictive ability obtained from univariate sERRBLUP is marginally higher than univariate GBLUP only when the top 0.01 percent of interactions are kept in the model. When the selection of effects is too strict, with only 0.001 percent of interactions used, the predictive ability of univariate sERRBLUP within Bernburg is smaller than the one obtained with GBLUP, especially if the selection is based on effect sizes.

**Fig. 3:**
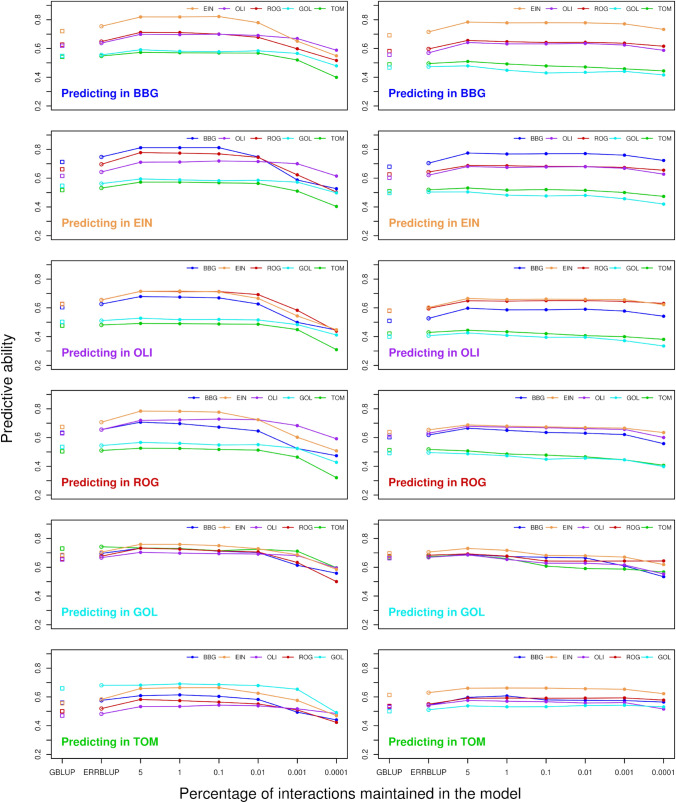
Predictive ability for bivariate GBLUP (open squares), bivariate ERRBLUP (open circles), and bivariate sERRBLUP (filled circles and solid lines) when SNP interaction selections are based on estimated effects variances in KE (left side) and PE (right side) for trait PH_V4. In each panel, the solid lines’ color indicates the additional environment used to predict the target environment

The predictive abilities of bivariate GBLUP, ERRBLUP, and sERRBLUP when SNP interactions were selected based on estimated effect variances are compared for trait PH_V4 in KE and PE in Fig. 3. Figure 3 shows that the bivariate ERRBLUP increases the predictive ability slightly compared to bivariate GBLUP with the maximum absolute increase of 0.03 in KE and 0.02 in PE across all environments’ combinations. A considerable increase in predictive ability is obtained in bivariate sERRBLUP mostly when the top 5 or 1 percent of interactions are maintained in the model. However, the bivariate sERRBLUP predictive abilities decrease dramatically for too stringent selection of pairwise SNP interactions such as 0.01, 0.001, or 0.0001 percent. Moreover, the reduction in predictive ability with too stringent factor selection is more severe for KE than for PE. This pattern is observed for the majority of environments for both landraces, and the results for other traits are shown in the supplementary (Fig. S3b–S9b).

**Fig. 4:**
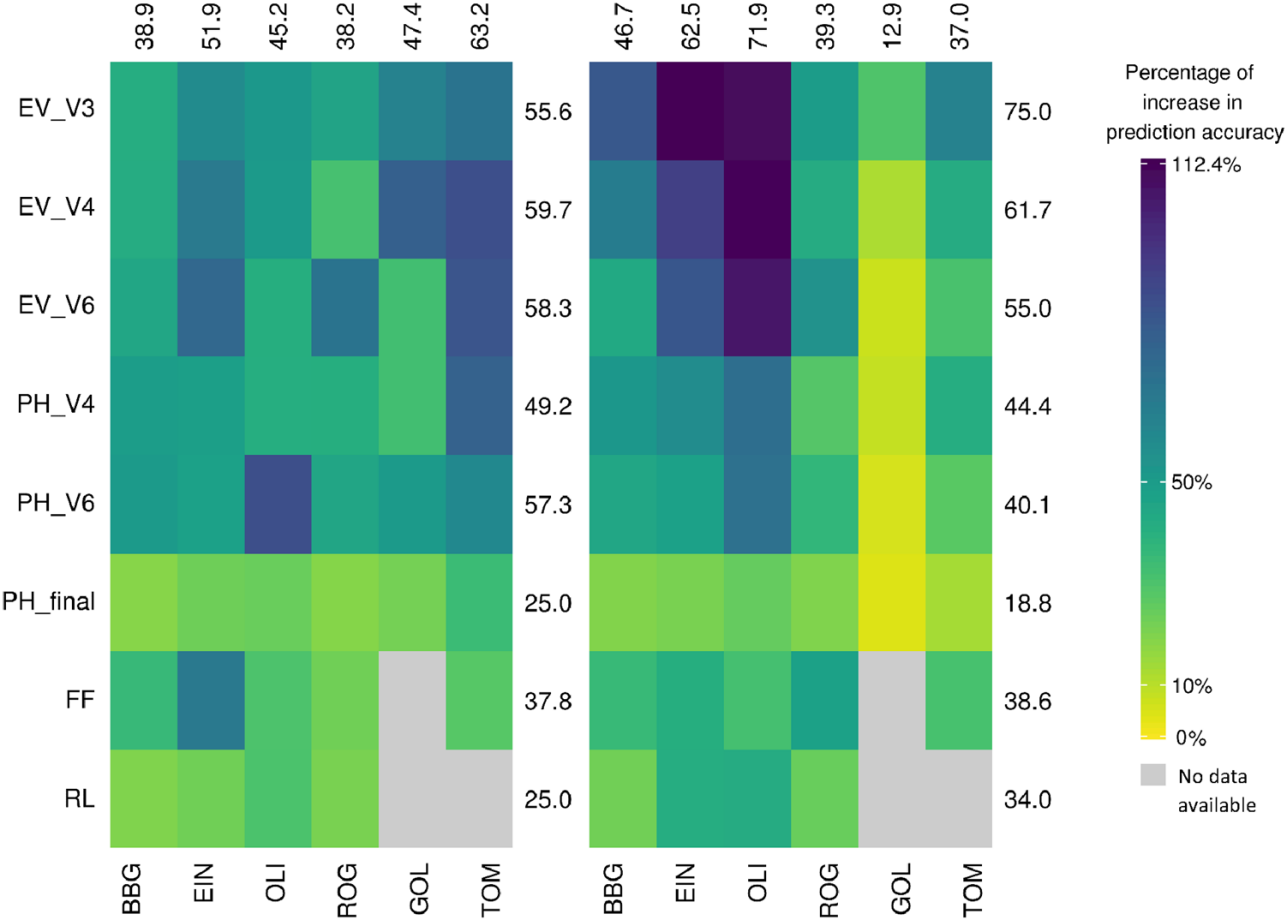
Percentage of increase in prediction accuracy from univariate GBLUP within environments to the maximum prediction accuracy of univariate sERRBLUP across environments when the SNP interaction selections are based on estimated effects variances in KE (left side) and in PE (right side). The average percentage of increase in prediction accuracy for each trait and environments are displayed in rows and columns, respectively

The relative increase in prediction accuracy of the best univariate sERRBLUP across environments compared to univariate GBLUP within environments for all traits and all locations is shown in form of a heat map in Fig. 4 for both landraces. The maximum relative increase in prediction accuracy among all traits and all environments in KE is 85.6 percent (PH_V6 in OLI), and in PE it is 112.4 percent (EV_V3 in EIN). Those highest increases in accuracy were found in traits and environment combinations where the univariate GBLUP prediction accuracy was particularly low. An increase is observed in each studied trait by location combination, with the smallest increase in both landraces for PH_final in BBG (20.1 percent in KE) or in GOL (5.9 percent in PE). In general, both plots in Fig. 4 demonstrate that for the majority of traits and environments, there is more than a 30 percent increase in prediction accuracy from univariate GBLUP within environments to the best univariate sERRBLUP across environments. The average increase across all combinations in KE is 47.1 percent and in PE is 46.7 percent. Note that this increase is somewhat inflated as a single GBLUP accuracy is compared against the best prediction from a set of various models (environment/selection proportions). However, even when using a set environment and a fixed proportion of interactions, there are still substantial gain. Exemplary, EIN with a proportion of 0.1 still leads to an increase of 43.1 percent in KE and 36.9 percent in PE (Fig. S14). The choice of EIN was made as it had the highest number of phenotyped lines (Table S1), while 0.1 in general led to stable models. Results using any other location or reasonable choice of the share of included interactions were very similar. The absolute increase in prediction accuracy is also shown as a heat map in Supplementary Fig. S15a, which indicates the average absolute increase of 0.204 in KE and 0.181 in PE.

**Fig. 5:**
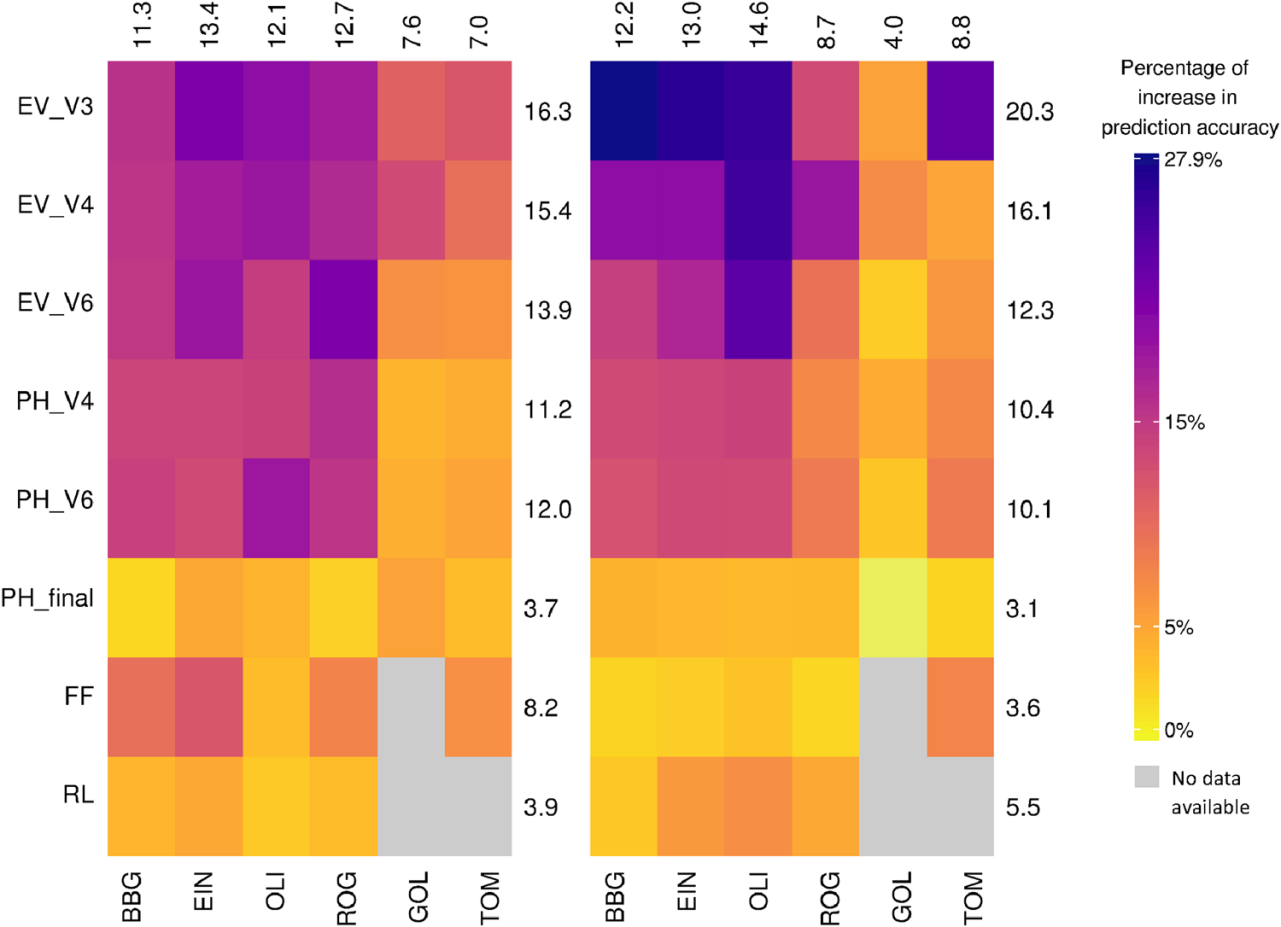
Percentage of increase in prediction accuracy from the maximum bivariate GBLUP to the maximum prediction accuracy of bivariate sERRBLUP when the SNP interaction selections are based on estimated effects variances in KE (left side) and in PE (right side). The average percentage of increase in prediction accuracy for each trait and environments are displayed in rows and columns, respectively

Figure 5 also shows the relative increase in prediction accuracy from the best bivariate GBLUP to the best bivariate sERRBLUP for all traits and all locations. The maximum increase in prediction accuracy among all traits and all environments is 21.1 percent (EV_V6 in ROG) in KE and 27.9 percent (EV_V3 in BBG) in PE. There is an increase across all studied traits in all environments except for the trait PH_final in PE which shows a relative decrease of 0.3 percent. The minimum increase in prediction accuracy in KE was also observed for PH_final (1.7 percent). In general, Fig. 5 shows that the relative increase in prediction accuracy from the best bivariate GBLUP to the best bivariate sERRBLUP is more than 7 percent for the majority of trait by location combinations in both landraces with an average increase of 10.9 percent in KE and 10.5 in PE across all combinations. The absolute increase in prediction accuracy of bivariate models is also shown as a heat map in supplementary (Fig. S15b) indicating an average absolute increase of 0.1 across all traits, environment combinations, and landraces.

**Fig. 6:**
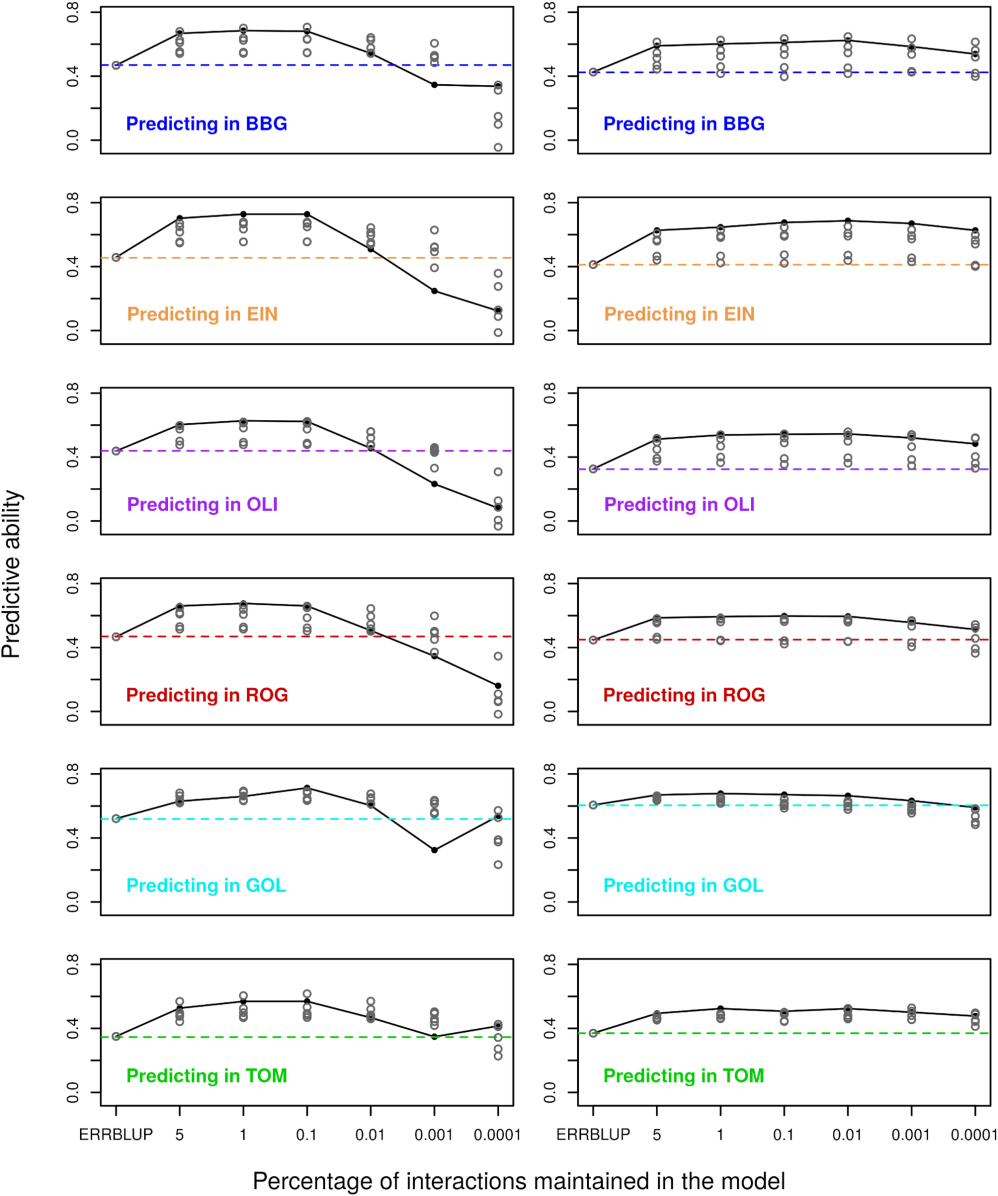
Predictive ability for univariate GBLUP within environment (dashed horizontal line), univariate ERRBLUP within environment (gray open circle), univariate sERRBLUP using a single environment for selecting the SNP interactions (gray open circles), and univariate sERRBLUP using all 5 environments jointly (filled black circles and solid line) for the SNP interaction selection based on estimated effects variances for trait PH_V4 in KE (left side) and PE (right side)

In addition to assessing the predictive ability of univariate sERRBLUP based on a single environment, Fig. 6 displays the comparison between the predictive ability obtained from univariate GBLUP and univariate ERRBLUP within environments, and univariate sERRBLUP across multiple environments jointly for trait PH_V4 in KE and PE. It is demonstrated that univariate sERRBLUP has a higher predictive ability than univariate GBLUP when interactions are selected based on all the other five environments jointly. The preliminary analysis also reveals the robustness of the selection strategy based on the effects variance compared to selection strategy based on the absolute effects sizes in univariate sERRBLUP across multiple environments jointly for KE (Fig. S16), while for PE it does not show a significant difference for the interaction selection strategy (Fig. S17). Figure 6 demonstrates that the predictive ability of univariate sERRBLUP across multiple environments jointly is as good as or better than using a single environment with few exceptions when the selection of effects is not too strict. With less than 0.1 percent of interactions used, predictive abilities deteriorate (especially so in KE) and selection from combined environments turns out to be worse than selection from single environments.

**Fig. 7:**
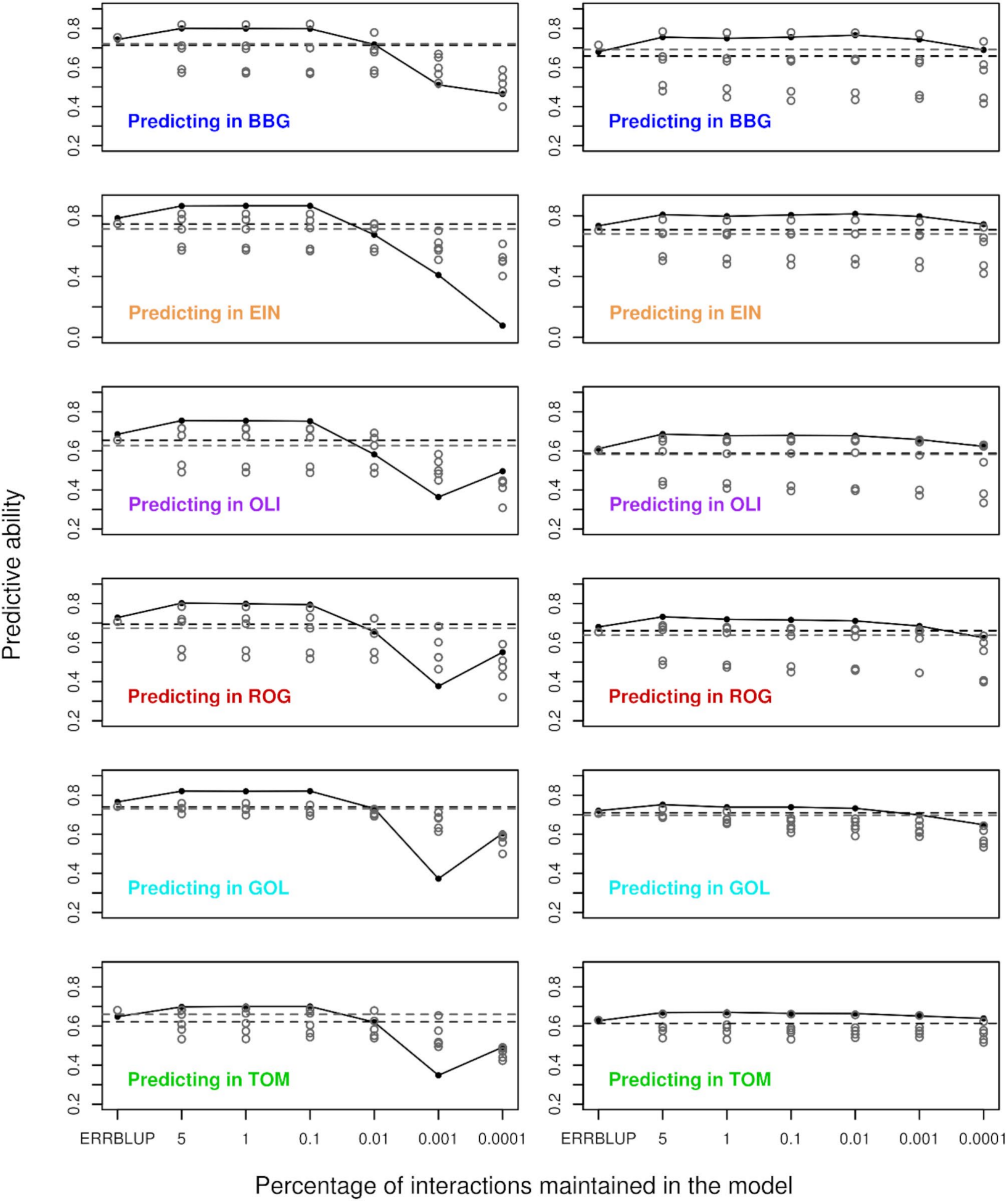
Predictive ability for bivariate GBLUP (black dashed horizontal line), bivariate ERRBLUP, and bivariate sERRBLUP (filled black circles) for the SNP interaction selection based on estimated effects variances using all 5 environments jointly for trait PH-V4 in KE (left side) and PE (right side). In each panel, gray horizontal line and first gray open circles refer to maximum bivariate GBLUP and maximum bivariate ERRBLUP, and the gray open circles at the top 5, 1, 0.1, 0.01, 0.001, 0.0001 quantiles refer to bivariate sERRBLUP using a single environment as an additional environment

Figure 7 illustrates the comparison between the predictive ability of bivariate GBLUP, ERRBLUP, and sERRBLUP across multiple environments jointly and the maximum predictive ability of bivariate GBLUP and ERRBLUP and all the predictive abilities of sERRBLUP when a single environment is considered as an additional environment for the trait PH_V4 in both KE and PE. The results indicate that bivariate sERRBLUP across multiple environments jointly increases the predictive ability compared to bivariate GBLUP and ERRBLUP across multiple environments jointly. In most cases, bivariate GBLUP, ERRBLUP, and sERRBLUP across multiple environments jointly perform as good as or better than when using a single environment.

## Discussion

The accuracy of genomic prediction when incorporating epistasis interactions in the model compared to prediction models with only main effects has been widely discussed over the last years. In particular, it was found that accounting for epistasis can increase predictive ability (Carlborg and Haley 2004; Hu et al. 2011; Huang et al. 2012; Wang et al. 2012; Mackay 2014; Jiang and Reif 2015; Ober et al. 2015; Rönnegård and Shen 2016).

A major concern in utilizing epistasis models has been the high computational load (Mackay 2014) which has been reduced for the full model including all interactions by utilizing marker based epistasis relationship matrices derived from Hadamard products of additive genomic relationship matrices (Jiang and Reif 2015; Ober et al. 2015; Martini et al. 2016). The key advantage of this approach is that the number of random effects in the model is reduced from the number of SNP interactions to the number of genotypes. While the approaches of Jiang and Reif (2015) and Martini et al. (2016) only capture the interactions whose products differ from zero (i.e., {22} genotype combinations for 0, 2 coded markers), our approach captures all possible genotype combinations ({00}, {02}, {20}, and {22}). Further, these epistasis relationship matrices and interaction effects were computed by bit-wise computations via the R-package miraculix (Schlather 2020), which carries out matrix multiplications about 15 times faster than regular matrix multiplications on genotype data in EpiGP R-package (Vojgani et al. 2021). In the analyzed datasets containing up to 30′212 SNPs (and thus 456′397′578 interactions), the computing time required to set up the sERRBLUP relationship matrix was about 810 min out of which around 330 min was required to estimate all pairwise SNP interaction effects and 480 min was required to set up the sERRBLUP relationship matrix for selected proportion of interactions by utilizing the R-package miraculix with 15 cores on a server cluster with Intel E5-2650 (2X12 core 2.2 GHz) processors. Computing times for sERRBLUP scale approximately quadratic in the number of markers were considered. The released EpiGP R-package (Vojgani et al. 2021), which is available at https://github.com/evojgani/EpiGP, has been utilized for ERRBLUP and sERRBLUP genomic prediction of phenotypes.

Our proposed epistasis model eventually can generate a considerable prohibitive computational load if the number of SNPs grows to hundreds of thousands (Vojgani et al. 2019). The computing time for sERRBLUP exhibits quadratic growth with increasing number of SNPs. A potential strategy to overcome these limitations is to achieve a feature reduction by SNP pruning, as was implemented in our maize dataset (Purcell et al. 2007; Chang et al. 2015). Another option to obtain an even stronger variable reduction than pruning might be the use of haplotype blocks (Pook et al. 2019). Although sERRBLUP model can be computationally challenging by increasing the number of SNPs, its predictive ability is constantly higher than the models such as RKHS, which reduces the computational time considerably (Table S10).

In this study, we showed that the predictive ability obtained by use of GBLUP and a full epistasis model with all pairwise SNP interactions included (ERRBLUP) was almost identical. In contrast, it was shown that the use of sERRBLUP increases predictive ability when only the most relevant SNP interactions are taken into account, regardless of the choice of the training environment, which is likely a result of enriching for true causal variant combinations among the list of all variant combinations used to construct the genetic covariance matrix. In our study, the maximum predictive ability with sERRBLUP was obtained by incorporating the top 5, 1, or 0.1 percent of pairwise SNP interactions, while a too strict selection of SNP interactions such as the top 0.01, 0.001, and 0.0001 percent often reduced the predictive ability. A similar loss in predictive ability with a too strict selection of interactions to be included in the model was also observed by Ober et al. (2015). The difference in interaction selection can be explained by the fact that the absolute number of interaction effects in the model is more important than the percentage of interaction effects. To illustrate this, the absolute numbers of interactions maintained in the model for the top 0.001 and 0.0001 percent of interactions in KE are, respectively, 3′235 and 323, which is less than the number of additive effects in KE (25′437) where the obtained sERRBLUP predictive ability is lower than GBLUP predictive ability. In addition, the possible differences in linkage can also lead to different redundancy patterns of interactions. Here we also saw the only major systematic difference between the two selection criteria: when SNP interactions were selected based on the magnitude of their estimated (absolute) effects, the loss in predictive ability when selecting too few interactions was much more severe than when SNP interactions were selected based on the variance associated with them. This phenomenon has been more prevalent in KE than in PE (Fig. S10–S11) and is valid in both scenarios, using information either from a single environment or from the average of all other environments (Fig. S16–S17). A potential reason for this is that the few interactions that remain in the model are highly linked, and thus, no proper representation of the overall population structure is possible anymore. This effect was even more pronounced when selecting based on effect sizes. Thus, we recommend the use of effect variances as a selection criterion in sERRBLUP applications since this should be conceptually more robust.

The bivariate models exhibited a considerably higher predictive ability than univariate models. In consequence, the bivariate GBLUP performed slightly better than the best univariate sERRBLUP in most cases (Fig. S18). Across all studied traits, the increase in prediction accuracy from GBLUP to sERRBLUP displays a similar pattern in both univariate and bivariate models. It has to be noted that this increase in predictive ability is exclusively caused by the modeling of epistasis in a bivariate statistical setting, while it is caused by both modeling of epistasis and borrowing information across environments through variable selection in the univariate statistical setting.

In general, it is expected that the predictive ability for phenotypes should be higher with higher heritability. In this study, the correlation between the heritability of all traits, which have been calculated on an entry-mean basis within each landraces (Hallauer et al. 2010) over all environments, was 0.296 with univariate GBLUP within environments and 0.543 with maximum univariate sERRBLUP across environments (Fig. S19a). Corresponding correlations were higher in the bivariate statistical setting of the respective models, with an increase in the respective correlation from maximum bivariate GBLUP (0.537) to maximum bivariate sERRBLUP (0.647) (Fig. S19b).

When comparing sERRBLUP to a traditional G × E model (Kang and Gorman 1989), the modeling approach is quite different. In sERRBLUP, the selection of marker-by-marker interactions is done based on a second environment. However, for the final estimation of the actual effect size, the data from the same environment are used. Thus, effect sizes can substantially differ between environments. In contrast with this, traditional G × E model will assign effects to specific marker-by-environment combinations. As included interactions between different environments in sERRBLUP are different, it is not possible to put concrete G × E effects on specific markers or marker-by-environment interactions, which would be the essence of traditional G × E models. As prediction performances are increasing quite substantially by the use of sERRBLUP, this still can be seen as an indication that effect regions are similar between environments (although effect sizes might differ).

Our results indicate that a higher number of phenotyped lines (in particular overlapping between environments) and including information from a more similar second environments were beneficial for prediction, e.g., when the two Spanish locations GOL or TOM were used as the second environment to predict a German environment, prediction accuracies were lower as these environments have substantially different climate and for some traits lower overlap between phenotyped lines. On the other hand, the best prediction results for GOL were obtained when using TOM as second environment and vice versa.

In both univariate and bivariate models, it was shown that the obtained predictive ability across multiple environments jointly was mostly equivalent or higher than the maximum predictive ability obtained based on a single environment. Thus, using an average across all other environments should be a robust alternative which in most cases will yield a result that is as good as or even better than the best single environment.

Overall, our results demonstrate that bivariate models can outperform univariate models and epistatic interactions can substantially increase the predictive ability. In the context of univariate models, it was shown that selecting a suitable subset of interactions based on other environments where phenotypic data of the full set of lines are available can substantially increase the predictive ability. As the ideal share $\pi$ of interactions to be included in sERRBLUP is not known in practice, one could consider to run a testing scheme with an additional validation set for the identification of a suitable $\pi$. As results were quite robust as long as a reasonable fraction (between 5 and 0.1 percent) of interactions were included in the model and this introduces further computational load, this should, however, usually not be required.

The presented approach can substantially improve the phenotype prediction accuracy in another environment by ‘borrowing’ information on effect regions from another variable. In our case, other variable was phenotypes of the same trait grown in different environments. However, one could also imagine using data from another growing season or even from a highly correlated second trait. This can be useful in sparse testing designs, e.g., where not all lines are grown in all environments. The suggested approach can be used to ‘impute’ missing phenotypes with a much increased accuracy compared to conventional approaches.

## Supplementary Information

Below is the link to the electronic supplementary material.

Supplementary file1 (PDF 5210 KB)

## Data Availability

All data and material are available through material transfer agreements upon request.

## References

[CR1] Abendroth LJ, Elmore RW, Boyer MJ, and Marlay SK (2011) Corn Growth and Development. PMR 1009. Iowa State Univ., Ames

[CR2] Aguilar I, Misztal I, Johnson DL, Legarra A, Tsuruta S, Lawlor TJ (2010). Hot topic: a unified approach to utilize phenotypic, full pedigree, and genomic information for genetic evaluation of Holstein final score. J Dairy Sci.

[CR3] Akdemir D and Godfrey OU (2015) EMMREML: Fitting Mixed Models with Known Covariance Structures. Available at: https://cran.r-project.org/package=EMMREML

[CR4] Akdemir D, Isidro-Sánchez J (2019). Design of training populations for selective phenotyping in genomic prediction. Sci Rep.

[CR5] Albrecht T, Wimmer V, Auinger H-J, Erbe M, Knaak C, Ouzunova M, Simianer H, Schön C-C (2011). Genome-based prediction of testcross values in maize. Theor Appl Genet.

[CR6] Ben Hassen M, Bartholomé J, Valè G, Cao T-V, Ahmadi N (2018). Genomic prediction accounting for genotype by environment interaction offers an effective framework for breeding simultaneously for adaptation to an abiotic stress and performance under normal cropping conditions in rice. G3.

[CR7] Bernardo R, Yu J (2007). Prospects for genomewide selection for quantitative traits in maize. Crop Sci.

[CR8] Browning SR, Browning BL (2007). Rapid and accurate haplotype phasing and missing data inference for whole genome association studies by use of localized haplotype clustering. Am J Hum Genet.

[CR9] Burgueño J, de Los Campos G, Weigel K, Crossa J (2012). Genomic prediction of breeding values when modeling genotype × environment interaction using pedigree and dense molecular markers. Crop Sci.

[CR10] Butler DG, Cullis BR, Gilmour AR, Gogel BJ, Thompson R (2018). ASReml-R Reference Manual Version 4.

[CR11] Carlborg Ö, Haley CS (2004). Epistasis: too often neglected in complex trait studies?. Nat Rev Genet.

[CR12] Chang CC, Chow CC, Tellier LC, Vattikuti S, Purcell SM, Lee JJ (2015). Second-generation PLINK: rising to the challenge of larger and richer datasets. Gigascience.

[CR13] Covarrubias-Pazaran G, Schlautman B, Diaz-Garcia L, Grygleski E, Polashock J, Johnson-Cicalese J, Vorsa N, Iorizzo M, Zalapa J (2018). Multivariate GBLUP improves accuracy of genomic selection for yield and fruit weight in biparental populations of vaccinium macrocarpon Ait. Front Plant Sci.

[CR14] Crossa J, de Los Campos G, Pérez P, Gianola D, Burgueño J, Araus JL, Makumbi D, Singh RP, Dreisigacker S, Yan J, Arief V, Banziger M, Braun H-J (2010). Prediction of genetic values of quantitative traits in plant breeding using pedigree and molecular markers. Genetics.

[CR15] Crossa J, Pérez P, de Los Campos G, Mahuku G, Dreisigacker S, Magorokosho C (2011). Genomic selection and prediction in plant breeding. Crop Improv.

[CR16] Da Y, Wang C, Wang S, Hu G (2014). Mixed model methods for genomic prediction and variance component estimation of additive and dominance effects using SNP markers. PLOS ONE.

[CR17] Daetwyler HD, Pong-Wong R, Villanueva B, Woolliams JA (2010). The impact of genetic architecture on genome-wide evaluation methods. Genetics.

[CR18] Daetwyler HD, Calus MPL, Pong-Wong R, de Los Campos G, Hickey JM (2013). Genomic prediction in animals and plants: simulation of data, validation, reporting, and benchmarking. Genetics.

[CR19] Filho J de A, Guimarães J, Silva FE, Resende M de, Muñoz P, Kirst M, Jr MR (2016) The contribution of dominance to phenotype prediction in a pine breeding and simulated population. Heredity 117(1): 33–41. 10.1038/hdy.2016.23 10.1038/hdy.2016.23 PMC4901355 27118156

[CR20] de Los CG, Gianila D, Rosa G, Weigel K, Crossa J (2010). Semi-parametric genomic-enabled prediction of genetic values using reproducing kernel Hilbert spaces methods. Gene Res.

[CR21] de Los Campos G, Naya H, Gianola D, Crossa J, Legarra A, Manfredi E, Weigel K, Cotes JM (2009). Predicting quantitative traits with regression models for dense molecular markers and pedigree. Genetics.

[CR22] de Los Campos G, Vazquez AI, Fernando R, Klimentidis YC, Sorensen D (2013). Prediction of complex human traits using the genomic best linear unbiased predictor. PLoS Genetics.

[CR23] Dekkers JCM (2007). Prediction of response to marker-assisted and genomic selection using selection index theory. J Anim Breed Genet.

[CR24] Desta ZA, Ortiz R (2014). Genomic selection: genome-wide prediction in plant improvement. Trends Plant Sci.

[CR25] Erbe M, Pimentel E, Sharifi AR, Simianer H (2010) Assessment of cross-validation strategies for genomic prediction in cattle. In: 9th world congress of genetics applied to livestock production, edited by German Society for Animal Science. German Society for Animal Science, Leipzig, Germany, pp 129–132

[CR26] Falconer DS, Mackay TFC (1996). Introduction to quantitative genetics.

[CR27] Gianola D, de Los Campos G, Hill WG, Manfredi E, Fernando R (2009). Additive genetic variability and the bayesian alphabet. Genetics.

[CR28] Gianola D, van Kaam JBCHM (2008). Reproducing Kernel hilbert spaces regression methods for genomic assisted prediction of quantitative traits. Genetics.

[CR29] Gianola D, Fernando RL, Stella A (2006). Genomic-assisted prediction of genetic value with semiparametric procedures. Genetics.

[CR30] Guo G, Zhao F, Wang Y, Zhang Y, Du L, Su G (2014). Comparison of single-trait and multiple-trait genomic prediction models. BMC Genet.

[CR31] Hallauer AR, Carena MJ, Miranda Filho JB (2010). Quantitative genetics in maize breeding.

[CR32] Hayes BJ, Goddard ME (2008). Technical note: prediction of breeding values using marker-derived relationship matrices. J Anim Sci.

[CR33] Hayes B, Goddard M (2010). Genome-wide association and genomic selection in animal breeding. Genome.

[CR34] He D, Kuhn D, Parida L (2016). Novel applications of multitask learning and multiple output regression to multiple genetic trait prediction. Bioinformatics.

[CR35] Henderson CR (1975) Best Linear Unbiased Estimation and Prediction under a Selection Model. Biometrics 31(2):423–447. 10.2307/2529430 1174616

[CR36] Henderson CR, Quaas RL (1976). Multiple trait evaluation using relatives’ records. J Anim Sci.

[CR37] Hill WG, Goddard ME, Visscher PM (2008). Data and theory point to mainly additive genetic variance for complex traits. PLoS Genetics.

[CR38] Hölker AC, Mayer M, Presterl T, Bolduan T, Bauer E, Ordas B, Brauner PC, Ouzunova M, Melchinger AE, Schön C-C (2019). European maize landraces made accessible for plant breeding and genome-based studies. Theor Appl Genet.

[CR39] Hu Z, Li Y, Song X, Han Y, Cai X, Xu S, Li W (2011). Genomic value prediction for quantitative traits under the epistatic model. BMC Genet.

[CR40] Huang W, Richards S, Carbone MA, Zhu D, Anholt RRH, Ayroles JF, Duncan L, Jordan KW, Lawrence F, Magwire MM, Warner CB, Blankenburg K, Han Y, Javaid M, Jayaseelan J, Jhangiani SN, Muzny D, Ongeri F, Perales L, Wu Y-Q, Zhang Y, Zou X, Stone EA, Gibbs RA, Mackay TFC (2012). Epistasis dominates the genetic architecture of Drosophila quantitative traits. Proc Natl Acad Sci USA.

[CR41] Jarquin D, Howard R, Crossa J, Beyene Y, Gowda M, Martini JWR, Covarrubias Pazaran G, Burgueño J, Pacheco A, Grondona M, Wimmer V, Prasanna BM (2020). Genomic prediction enhanced sparse testing for multi-environment trials. G3: Genes|Genomes|Genetics.

[CR42] Jiang Y, Reif JC (2015). Modeling epistasis in genomic selection. Genetics.

[CR43] Jiang Y, Reif JC (2020). Efficient algorithms for calculating epistatic genomic relationship matrices. Genetics.

[CR44] Jiao Y, Peluso P, Shi J, Liang T, Stitzer MC, Wang B, Campbell MS, Stein JC, Wei X, Chin C-S, Guill K, Regulski M, Kumari S, Olson A, Gent J, Schneider KL, Wolfgruber TK, May MR, Springer NM, Antoniou E, McCombie WR, Presting GG, McMullen M, Ross-Ibarra J, Dawe RK, Hastie A, Rank DR, Ware D (2017). Improved maize reference genome with single-molecule technologies. Nature.

[CR45] Jones B (2012). Predicting phenotypes. Nat Rev Genet.

[CR46] Kang MS, Gorman DP (1989). Genotype × environment interaction in maize. Agron J.

[CR47] Karaman E, Cheng H, Firat MZ, Garrick DJ, Fernando RL (2016). An upper bound for accuracy of prediction using GBLUP. PLoS ONE.

[CR48] Legarra A, Christensen OF, Aguilar I, Misztal I (2014). Single Step, a general approach for genomic selection. Livest Sci.

[CR49] Mackay TFC (2014). Epistasis and quantitative traits: using model organisms to study gene-gene interactions. Nat Rev Genet.

[CR50] Martini JWR, Wimmer V, Erbe M, Simianer H (2016). Epistasis and covariance: how gene interaction translates into genomic relationship. Theor Appl Genet.

[CR51] Martini JWR, Gao N, Cardoso DF, Wimmer V, Erbe M, Cantet RJC, Henner S (2017). Genomic prediction with epistasis models: on the marker-coding-dependent performance of the extended GBLUP and properties of the categorical epistasis model (CE). BMC Bioinfo.

[CR52] Martini JWR, Rosales F, Ha N-T, Heise J, Wimmer V, Kneib T (2019). Lost in translation: on the problem of data coding in penalized whole genome regression with interactions. G3: Gene|Genomes|Genetics.

[CR53] Martini JWR, Toledo FH, Crossa J (2020). On the approximation of interaction effect models by hadamard powers of the additive genomic relationship. Theor Popul Biol.

[CR54] Meuwissen THE, Hayes BJ, Goddard ME (2001). Prediction of total genetic value using genome-wide dense marker maps. Genetics.

[CR55] Momen M, Mehrgardi AA, Sheikhi A, Kranis A, Tusell L, Morota G, Rosa GJM, Gianola D (2018). Predictive ability of genome-assisted statistical models under various forms of gene action. Sci Rep.

[CR56] Montesinos-López OA, Montesinos-López A, Crossa J, Toledo FH, Pérez-Hernández O, Eskridge KM, Rutkoski J (2016). A Genomic bayesian multi-trait and multi-environment model. G3: Genes|Genomes|Genetics.

[CR57] Mrode RA (2014). Linear models for the prediction of animal breeding values.

[CR58] Ober U, Huang W, Magwire M, Schlather M, Simianer H, Mackay TFC (2015). accounting for genetic architecture improves sequence based genomic prediction for a drosophila fitness trait. PLOS ONE Public Library Sci.

[CR59] Pérez P, de Los CG, Crossa J, Gianola D (2010). Genomic-Enabled prediction based on molecular markers and pedigree using the bayesian linear regression package in R. Plant Genome.

[CR60] Pook T, Schlather M, de Los Campos G, Mayer M, Schoen CC, Simianer H (2019). HaploBlocker: creation of subgroup specific haplotype blocks and libraries. Genetics.

[CR61] Pook T, Mayer M, Geibel J, Weigend S, Cavero D, Schoen CC, Simianer H (2020). Improving imputation quality in BEAGLE for crop and livestock data. G3: Genes|Genomes|Genetics.

[CR62] Purcell S, Neale B, Todd-Brown K, Thomas L, Ferreira MAR, Bender D, Maller J, Sklar P, de Bakker PIW, Daly MJ, Sham PC (2007). PLINK: a tool set for whole-genome association and population-based linkage analyses. Am J Hum Genet.

[CR63] Rönnegård L, Shen X (2016) Genomic prediction and estimation of marker interaction effects. bioRxiv. 10.1101/038935

[CR64] Schlather M (2020) Efficient Calculation of the Genomic Relationship Matrix. bioRxiv. 10.1101/2020.01.12.903146

[CR65] Schrauf MF, Martini JWR, Simianer H, de Los Campos G (2020). Phantom epistasis in genomic selection: on the predictive ability of epistatic models. G3: Genes|Genomes|Genetics.

[CR66] Schulthess AW, Zhao Y, Longin CFH, Reif JC (2018). Advantages and limitations of multiple-trait genomic prediction for Fusarium head blight severity in hybrid wheat (Triticum aestivum L.). Theor Appl Genetics.

[CR67] Stich B, Van Ingheland D (2018). Prospects and potential uses of genomic prediction of key performance traits in tetraploid potato. Front in Plant Sci.

[CR68] Unterseer S, Bauer E, Haberer G, Seidel M, Knaak C, Ouzunova M, Meitinger T, Strom TM, Fries R, Pausch H, Bertani C, Davassi A, Mayer KF, Schön C-C (2014). A powerful tool for genome analysis in maize: 584 development and evaluation of the high density 600 k SNP genotyping array. BMC Genomics.

[CR69] Utz HF, Melchinger AE, Schön CC (2000) Bias and sampling error of the estimated proportion of genotypic variance explained by quantitative trait Loci determined from experimental data in maize using cross validation and validation with independent samples. Genetics 154(4):1839–1849. 10.1093/genetics/154.4.1839 PMC1461020 10866652

[CR70] VanRaden P (2007). Efficient estimation of breeding values from dense genomic data. J Dairy Sci.

[CR71] VanRaden P (2008). Efficient methods to compute genomic predictions. J Dairy Sci.

[CR72] Velazco JG, Jordan DR, Mace ES, Hunt CH, Malosetti M, van Eeuwijk FA (2019). Genomic prediction of grain yield and drought-adaptation capacity in sorghum is enhanced by multi-trait analysis. Front Plant Sci.

[CR73] Vojgani E, Pook T and Simianer H (2019) EpiGP: Epistatic relationship matrix based genomic prediction of phenotypes. https://github.com/evojgani/EpiGP

[CR74] Vojgani E, Pook T, and Simianer H (2021) Phenotype prediction under epistasis. Epistasis: Methods in molecular biology, vol 2212. Humana, New York, pp 105–120. 10.1007/978-1-0716-0947-7_8 10.1007/978-1-0716-0947-7_8 33733353

[CR75] Wang D, El-Basyoni IS, Baenziger PS, Crossa J, Eskridge KM, Dweikat I (2012). Prediction of genetic values of quantitative traits with epistatic effects in plant breeding populations. Heredity.

[CR76] Wang J, Zhou Z, Zhe Z, Li H, Liu D, Zhang Q, Bradbury PJ, Buckler ES, Zhiwu Z (2018). Expanding the BLUP alphabet for genomic prediction adaptable to the genetic architectures of complex traits. Heredity.

[CR77] Wimmer V, Lehermeier C, Albrecht T, Auinger H-J, Wang Y, Schön C-C (2013). Genome-wide prediction of traits with different genetic architecture through efficient variable selection. Genetics.

[CR78] Windhausen VS, Atlin GN, Hickey JM, Crossa J, Jannink J-L, Sorrells ME, Raman B, Cairns JE, Tarekegne A, Semagn K, Beyene Y, Grudloyma P, Technow F, Riedelsheimer C, Melchinger AE (2012). Effectiveness of genomic prediction of maize hybrid performance in different breeding populations and environments. G3.

[CR79] Wolc A, Stricker C, Arango J, Settar P, Fulton JE, O’Sullivan NP, Preisinger R, Habier D, Fernando R, Garrick DJ, Lamont SJ, Dekkers JCM (2011). Breeding value prediction for production traits in layer chickens using pedigree or genomic relationships in a reduced animal model. Genetics Sel Evol.

[CR80] Wright S (1931). Evolution in mendelian populations. Genetics.

