# Supplementary material for: Accounting for epistasis improves genomic prediction of phenotypes with univariate and bivariate models across environments
Source: Theor Appl Genet. 2021 Jun 11;134(9):2913–30. doi: 10.1007/s00122-021-03868-1 (PMC8354961; doi:10.1007/s00122-021-03868-1)
Supplement: Supplementary file 1 — Supplementary file1 (PDF 5210 KB) [file 122_2021_3868_MOESM1_ESM.pdf]

Theoretical and Applied Genetics

Accounting for epistasis improves genomic prediction of phenotypes with univariate and bivariate models across environments

Elaheh Vojgani[1*], Torsten Pook[1], Johannes W.R. Martini[2], Armin C. Hölker[3], Manfred Mayer[3], Chris-Carolin Schön[3], Henner Simianer[1]

[1] University of Goettingen, Center for Integrated Breeding Research, Animal Breeding and Genetics Group, Goettingen, Germany; [2] International Maize and Wheat Improvement Center (CIMMYT), Texcoco, State of Mexico, Mexico; [3] Plant Breeding, TUM School of Life Sciences Weihenstephan, Technical University of Munich, Freising, Germany

Corresponding author

Correspondence to Elaheh Vojgani

Email: vojgani@gwdg.de

ORCID: 0000-0003-4375-3531

Supplemental tables

Table S1 Phenotypic traits descriptions, locations, number of lines, mean, minimum, maximum and standard deviation of phenotypic traits in each location for KE (blue numbers) and PE (red numbers).

Trait	Definition	Location	Number of Phenotyped lines	Mean	Minimum	Maximum	Standard deviation
**EV_V3**	Early vigour at V3 stage scored on scale from 1 (very poor early vigour) to 9 (very high early vigour)	**BBG**	459\393	4.10\4.68	0.78\1.00	7.28\7.55	1.28\1.18
		**EIN**	462\393	4.11\4.70	0.86\1.03	9.00\9.03	1.31\1.18
		**OLI**	440\389	5.31\6.17	1.22\3.30	8.05\8.74	1.15\0.86
		**ROG**	460\390	5.35\5.84	1.71\2.90	7.90\7.92	0.95\0.75
		**GOL**	210\204	6.31\6.67	4.07\5.49	8.49\7.98	0.69\0.51
		**TOM**	210\204	5.51\6.15	1.93\3.84	7.34\8.45	0.99\0.67
**EV _V4**	Early vigour at V4 stage scored on scale from 1 (very poor early vigour) to 9 (very high early vigour)	**BBG**	459\393	3.85\4.65	0.67\0.93	8.29\8.49	1.48\1.48
		**EIN**	462\393	4.24\4.82	0.94\1.52	7.07\7.46	1.11\0.98
		**OLI**	439\389	5.27\6.07	0.80\2.99	7.52\8.36	1.08\0.75
		**ROG**	459\390	5.44\5.85	2.65\2.88	7.86\7.94	0.92\0.78
		**GOL**	210\204	5.71\5.98	3.37\3.91	7.89\7.89	0.81\0.83
		**TOM**	210\204	5.26\5.75	2.59\3.92	6.89\7.35	0.83\0.61
**EV _V6**	Early vigour at V6 stage scored on scale from 1 (very poor early vigour) to 9 (very high early vigour)	**BBG**	459\393	3.92\4.64	0.74\0.84	8.75\8.22	1.39\1.41
		**EIN**	462\393	5.03\5.54	0.97\1.51	8.05\8.39	1.24\1.06
		**OLI**	437\388	5.30\6.07	0.54\3.56	7.17\8.09	0.96\0.74
		**ROG**	461\390	5.55\5.91	1.02\2.52	8.07\7.76	0.95\0.77
		**GOL**	210\204	6.24\6.24	3.90\3.81	8.45\7.94	0.85\0.85
		**TOM**	210\204	5.58\5.86	2.96\3.90	7.66\7.91	0.92\0.68
**PH_V4**	Mean plant height of three plants of the plot at V4 stage in cm	**BBG**	461\393	35.86\41.48	9.27\16.38	60.40\62.85	8.43\7.93
		**EIN**	462\393	34.49\38.73	6.90\20.43	53.14\57.94	7.24\6.17
		**OLI**	440\388	18.43\22.55	7.35\11.89	31.11\35.75	3.93\3.87
		**ROG**	460\390	25.50\28.10	9.23\13.63	42.29\41.54	4.60\4.53
		**GOL**	210\204	62.88\68.98	34.30\38.39	88.24\95.30	9.79\10.96
		**TOM**	210\204	41.60\47.45	11.98\25.37	63.89\72.12	8.71\8.27

Trait	Definition	Location	Number of Phenotyped lines	Mean	Minimum	Maximum	Standard deviation
**PH_V6**	Mean plant height of three plants of the plot at V6 stage in cm	**BBG**	461\393	61.75\69.08	19.41\30.36	93.84\100.39	11.80\11.12
		**EIN**	462\393	62.40\69.36	21.41\36.53	95.54\98.80	11.89\9.62
		**OLI**	440\390	36.74\45.35	8.34\14.78	58.40\72.48	8.69\8.53
		**ROG**	459\390	61.46\68.91	32.17\30.35	89.74\94.77	9.34\9.52
		**GOL**	210\204	94.21\98.30	37.28\54.75	127.54\130.51	15.05\15.29
		**TOM**	210\204	83.86\92.35	48.46\57.81	119.07\124.98	14.41\12.79
**PH_final**	Final plant height after flowering in cm	**BBG**	461\393	142.65\120.60	95.85\59.78	210.08\179.03	20.53\19.11
		**EIN**	462\393	159.18\141.35	100.84\69.01	228.96\211.14	21.57\21.10
		**OLI**	432\387	118.17\112.46	58.74\58.55	175.81\173.15	21.95\20.48
		**ROG**	461\390	137.04\122.25	74.25\63.56	211.14\201.92	22.32\20.56
		**GOL**	209\204	115.68\102.69	49.27\30.21	167.58\149.14	21.73\23.59
		**TOM**	210\204	157.99\144.61	81.92\79.28	245.00\195.36	24.82\18.95
**FF**	Days after sowing until female flowering (days until 50% of the plot showed silks)	**BBG**	461\393	82.10\82.08	69.45\69.78	95.74\92.04	4.31\4.18
		**EIN**	462\393	82.55\81.78	70.36\68.86	102.02\101.50	5.23\5.17
		**OLI**	346\347	83.32\82.41	72.60\69.46	92.13\91.54	3.76\3.68
		**ROG**	458\389	73.06\71.91	62.45\59.10	91.22\88.03	4.82\4.47
		**TOM**	209\203	76.88\74.16	63.93\62.13	93.28\92.17	5.58\4.64
**RL**	Root lodging score from 1 to 9 (1 belonged to no lodging and 9 belonged to severe lodging)	**BBG**	461\392	5.02\3.03	0.59\0.03	9.58\9.22	2.78\2.39
		**EIN**	462\393	3.48\2.23	0.63\0.76	9.21\8.08	2.29\1.54
		**OLI**	439\388	2.59\1.80	0.59\0.52	9.15\7.65	1.64\1.19
		**ROG**	460\390	2.39\1.50	0.96\0.95	9.01\8.50	2.21\1.13

Table S2 The percentage of bivariate sERRBLUP model convergence in 5-fold cross validation with 5 replicates for trait PH-V4 for both KE and PE (black percentages), only KE (blue percentages) and only PE (red percentages). The starts represent the non-convergence of pre estimated variance components based on the full set.

Predicted Environment	Additional Environment	Top 5	Top 1	Top 0.1	Top 0.01	Top 0.001
**BBG**	**EIN**	100%	100%	100%	100%	100%*
**BBG**	**GOL**	0%*	8%*	12%	100%	100%
**EIN**	**BBG**	100%	100%	100%	100%	100%*
**EIN**	**GOL**	100%*	100%*	28%*	100%	100%
**OLI**	**GOL**	16%*	16%*	100%*	100%	100%
**ROG**	**BBG**	100%	100%	100%	100%	100%*
**ROG**	**GOL**	100%*	100%*	100%*	100%	100%
**TOM**	**BBG**	100%*	100%*	100%*	0%*	100%*
**TOM**	**EIN**	100%	100%*	100%*	96%	100%*
**TOM**	**GOL**	100%*	100%*	100%*	64%	100%*

Table S3 The percentage of bivariate sERRBLUP model convergence in 5-fold cross validation with 5 replicates for trait EV_V3 for both KE and PE (black percentages), only KE (blue percentages) and only PE (red percentages). The starts represent the non-convergence of pre estimated variance components based on the full set.

Predicted Environment	Additional Environment	Top 5	Top 1	Top 0.1	Top 0.01	Top 0.001
**BBG**	**EIN**	100%	100%	100%	100%	96%*
**BBG**	**GOL**	96%	100%	100%	100%	100%
**BBG**	**TOM**	100%	100%	100%	100%	88%
**EIN**	**ROG**	100%	100%	92%	96%	92%
**EIN**	**BBG**	100%	100%	100%	100%	88%*
**EIN**	**TOM**	100%	100%	96%	96%	96%
**OLI**	**ROG**	100%	100%	100%	92%	96%
**OLI**	**EIN**	100%	100%	100%	92%	0%* / 96%
**OLI**	**BBG**	100%	100%	96%	96% / 88%	100%* / 76%
**OLI**	**GOL**	96%	96%	88%	96%	92%
**OLI**	**TOM**	92%	96%	72%	72%*	60%*
**ROG**	**EIN**	100%	100%	96%	52%*	0%* / 76%
**ROG**	**BBG**	100%	100%	100%	96% / 92%	0%*
**ROG**	**GOL**	100%	100%	96%	92%	96%
**ROG**	**TOM**	100%	100%	100%	84%	100%
**GOL**	**ROG**	100%	100%	100%	92%	100%
**GOL**	**EIN**	100%	100%	100%	100%	0%*
**GOL**	**BBG**	100%	100%	96%	96%	80%* / 96%
**GOL**	**OLI**	96%	92%	96%	92%	88%
**GOL**	**TOM**	100%	100%	100%	88% / 80%	80% / 96%
**TOM**	**EIN**	96%	92%	64%	44%*	0%*
**TOM**	**BBG**	100%	100%	100%	96%	80%*
**TOM**	**OLI**	96%	100%	100%	100%	92%
**TOM**	**GOL**	0%*	96%	100%	80%	96% / 96%

Table S4 The percentage of bivariate sERRBLUP model convergence in 5-fold cross validation with 5 replicates for trait EV_V4 for both KE and PE (black percentages), only KE (blue percentages) and only PE (red percentages). The starts represent the non-convergence of pre estimated variance components based on the full set.

Predicted Environment	Additional Environment	Top 5	Top 1	Top 0.1	Top 0.01	Top 0.001
**BBG**	**EIN**	100%	100%	100%	100%	12%*
**BBG**	**GOL**	100%	100%	96%	68%	56%
**EIN**	**OLI**	100%	96%	100%	100%	100%
**OLI**	**EIN**	88%	88%	96% / 16%*	96% / 4%*	8%* / 8%*
**OLI**	**BBG**	100%	100%	96%	100%	100%
**OLI**	**GOL**	100%	100%	100%	96%	100%
**OLI**	**TOM**	100%	100%	100%	96%	96%
**ROG**	**EIN**	100%	100%	100%	100%	0%*
**GOL**	**EIN**	100%	100%	100%	100%	0%*
**GOL**	**TOM**	100%	96%	100%	100%	100%
**TOM**	**ROG**	100%	96%	100%	92%	100%
**TOM**	**EIN**	100%	96%	88%	100%	100%
**TOM**	**BBG**	100%	96%	25%*	40%*	80%
**TOM**	**GOL**	80%	80%	96% / 48%*	96%	80%

Table S5 The percentage of bivariate sERRBLUP model convergence in 5-fold cross validation with 5 replicates for trait EV_V6 for both KE and PE (black percentages), only KE (blue percentages) and only PE (red percentages). The starts represent the non-convergence of pre estimated variance components based on the full set.

Predicted Environment	Additional Environment	Top 5	Top 1	Top 0.1	Top 0.01	Top 0.001
BBG	ROG	100%	100%	100%	100%	80%*
BBG	EIN	100%	100%	96%	92%	0%*
BBG	GOL	0%* / 25%*	0%* / 48%*	24%* / 24%*	0%*	100%
EIN	ROG	100%	100%	96%	80%* / 96%	52%* / 96%
EIN	GOL	4%* / 0%*	4%* / 88%*	48%* / 0%*	32%	100%
OLI	EIN	100%	100%	88%	88%	4%* / 84%
OLI	ROG	100%	100%	100%	100%	0%*
OLI	GOL	25%* / 0%*	72% / 96%	24%* / 4%*	12%*	100%
ROG	EIN	100%	100%	92%	80%	40%* / 88%
ROG	BBG	96%	96%	100%	96% / 84%*	96% / 52%*
ROG	OLI	100%	100%	96%	100%	96%
ROG	GOL	25% / 0%*	0%* / 96%	0%* / 36%	0%	100%
GOL	ROG	100%	100%	100%	100%	96%*
GOL	EIN	100%	100%	100%	100%	80%*
TOM	ROG	100%	96%	24%*	60%* / 96%	0%* /72%
TOM	EIN	100%	100%	72%	72%	0%*
TOM	BBG	100%	76%	76% / 96%	84%	84%
TOM	OLI	100%	100%	100%	100%	96%
TOM	GOL	0%* / 0%	0%* / 84%	0%* / 0%	16%* / 0%*	25%* / 60%*

Table S6 The percentage of bivariate sERRBLUP model convergence in 5-fold cross validation with 5 replicates for trait PH_V6 for both KE and PE (black percentages), only KE (blue percentages) and only PE (red percentages). The starts represent the non-convergence of pre estimated variance components based on the full set.

Predicted Environment	Additional Environment	Top 5	Top 1	Top 0.1	Top 0.01	Top 0.001
BBG	ROG	100%	100%	100%	96%	80%*
BBG	EIN	92%	100%	100%	100%	100%
BBG	OLI	100%	100%	100%	100%	36%
BBG	GOL	100%	100%	96%	96%	92%*
EIN	ROG	100%	100%	100%	100%	0%*
EIN	BBG	100%	100%	80%	100%	32%*
EIN	OLI	100%	100%	100%	100%	96%
EIN	GOL	100%	100%	100%	100%	100%*
OLI	ROG	100%	100%	96%	68%	16%*
OLI	EIN	100%	100%	100%	100%	92%
OLI	BBG	100%	100%	100%	100%	56%*
OLI	GOL	100%	100%	100%	96%	0%*
ROG	EIN	100%	100%	100%	100%	92%
ROG	BBG	100%	100%	100%	96%	84%*
ROG	OLI	100%	100%	100%	100%	0%*
ROG	GOL	100%	100%	100%	100%	8%*
GOL	ROG	100%	100%	100%	100%	96%*
GOL	BBG	100%	100%	100%	100%	0%* / 96%
GOL	OLI	100%	100%	100%	100%	0%*
TOM	ROG	100%	100%	92%	88%	40%*
TOM	EIN	100%	100%	100%	100%	100%
TOM	BBG	100%	100%	32%	92%	0%*
TOM	OLI	100%	100%	100%	96%	4%*
TOM	GOL	52%*	68%* / 88%	72%* / 92%	92%	16%* / 96%

Table S7 The percentage of bivariate sERRBLUP model convergence in 5-fold cross validation with 5 replicates for trait PH_final for both KE and PE (black percentages), only KE (blue percentages) and only PE (red percentages). The starts represent the non-convergence of pre estimated variance components based on the full set.

Predicted Environment	Additional Environment	Top 5	Top 1	Top 0.1	Top 0.01	Top 0.001
BBG	ROG	52%* / 0%*	0%*/ 0%*	0%*/ 0%*	0%*	100%
BBG	EIN	100%	100%	100%	100%	96%
BBG	TOM	36%*	4%*	0%*	92%	100%
EIN	ROG	0%*/ 25%	0%*/ 0%*	0%*/ 0%*	96% / 0%*	92%
EIN	BBG	8%*	4%*	0%*	96%	96%
EIN	OLI	100%	100%	100%	100%	92%
EIN	TOM	64%	4%*	0%*	100%	100%
OLI	ROG	0%*/ 4%*	0%*/ 0%*	0%*/ 0%*	0%*	96%
OLI	EIN	100%	96%	100%	100%	100%
OLI	BBG	4%*	0%*	0%*	100%	96%
OLI	GOL	100%	100%	100%	100%	100%
OLI	TOM	28%*	12%*	0%*	96% / 84%*	92%
ROG	EIN	100%	100%	100%	100%	96%
ROG	BBG	40%*	0%*	0%*	100%	100%
ROG	TOM	24%*	8%*	4%*	88%	100%
GOL	ROG	8%* / 0%*	4%* / 4%*	8%* / 4%*	0%*	100%
GOL	BBG	8%*	4%*	4%*	100%	100%
GOL	TOM	0%*	0%*	4%*	88%	100%
TOM	ROG	0%*/ 0%*	0%*/ 0%*	0%*/ 0%*	36%* / 0%*	48%* / 96%
TOM	EIN	100%	100%	84%	76%	40%* / 96%
TOM	BBG	8%*	0%*	0%*	88%	52%*
TOM	OLI	100%	100%	92%	92%	96%
TOM	GOL	100%	100%	96%	100%	96%

Table S8 The percentage of bivariate sERRBLUP model convergence in 5-fold cross validation with 5 replicates for trait FF for both KE and PE (black percentages), only KE (blue percentages) and only PE (red percentages). The starts represent the non-convergence of pre estimated variance components based on the full set.

Predicted Environment	Additional Environment	Top 5	Top 1	Top 0.1	Top 0.01	Top 0.001
BBG	EIN	4%*	4%*	0%*	92% / 32%*	80%
BBG	ROG	0%*	0%*	0%* / 0%*	80% / 0%*	68%
EIN	BBG	96% / 36%*	92% / 96%	92% / 0%*	88%* / 0%*	88% / 96%
EIN	ROG	0%*	0%*	0%* / 0%*	96% / 0%*	96% / 84%
OLI	ROG	0%*	92% / 0%*	16%* / 0%*	10 / 0%*	92% / 76%
OLI	BBG	100%	84%	0%*	4%*	96% / 32%*
OLI	EIN	64%*	20%	0%*	52%*	44%*
ROG	EIN	0%*	0%*	0%*	80%	52%*
ROG	BBG	40%	100%	0%*	0%*	96%
TOM	EIN	28%*	20%*	24%*	40%*	72% / 12%*
TOM	BBG	72%	100%	4%*	8%*	96%
TOM	OLI	100%	100%	96%	96%	96%
TOM	ROG	12%*	96% / 0%	0%* / 4%*	20%* / 4%*	4%* / 40%*

Table S9 The percentage of bivariate sERRBLUP model convergence in 5-fold cross validation with 5 replicates for trait RL for both KE and PE (black percentages), only KE (blue percentages) and only PE (red percentages). The starts represent the non-convergence of pre estimated variance components based on the full set.

Predicted Environment	Additional Environment	Top 5	Top 1	Top 0.1	Top 0.01	Top 0.001
**BBG**	**EIN**	100%	100%	100%	100%	96%
**EIN**	**ROG**	100%	100%	96%	96%	96%
**EIN**	**BBG**	100%	72%	60%*	20%*	96% / 16%*
**EIN**	**OLI**	92%	92%	92%	96%	68%
**ROG**	**EIN**	100%	100%	96%	88%	68%
**ROG**	**BBG**	100%	100%	100%	96%	96%
**ROG**	**OLI**	100%	100%	96%	100%	100%

Table S10 The predictive ability of RKHS, univariate GBLUP within environments, univariate ERRBLUP within environments and maximum univariate sERRBLUP across environments (blue numbers) and the maximum predictive ability of bivariate GBLUP, bivariate ERRBLUP and bivariate sERRBLUP (red numbers) in KE and PE for the trait PH_V4.

Landrace	Locations	RKHS	GBLUP	ERRBLUP	sERRBLUP
KE	BBG	0.479	0.470/0.721	0.468/0.754	0.707/0.822
	EIN	0.479	0.455/0.713	0.458/0.748	0.679/0.812
	OLI	0.435	0.439/0.627	0.438/0.655	0.623/0.717
	ROG	0.460	0.469/0.674	0.467/0.707	0.665/0.784
	GOL	0.543	0.519/0.730	0.521/0.742	0.694/0.759
	TOM	0.353	0.346/0.660	0.350/0.681	0.617/0.691
PE	BBG	0.423	0.424/0.692	0.426/0.715	0.648/0.784
	EIN	0.420	0.412/0.680	0.413/0.704	0.652/0.775
	OLI	0.334	0.324/0.582	0.326/0.602	0.558/0.665
	ROG	0.422	0.449/0.638	0.447/0.654	0.584/0.688
	GOL	0.590	0.604/0.697	0.606/0.706	0.664/0.731
	TOM	0.342	0.370/0.614	0.370/0.630	0.528/0.662

Supplemental figures

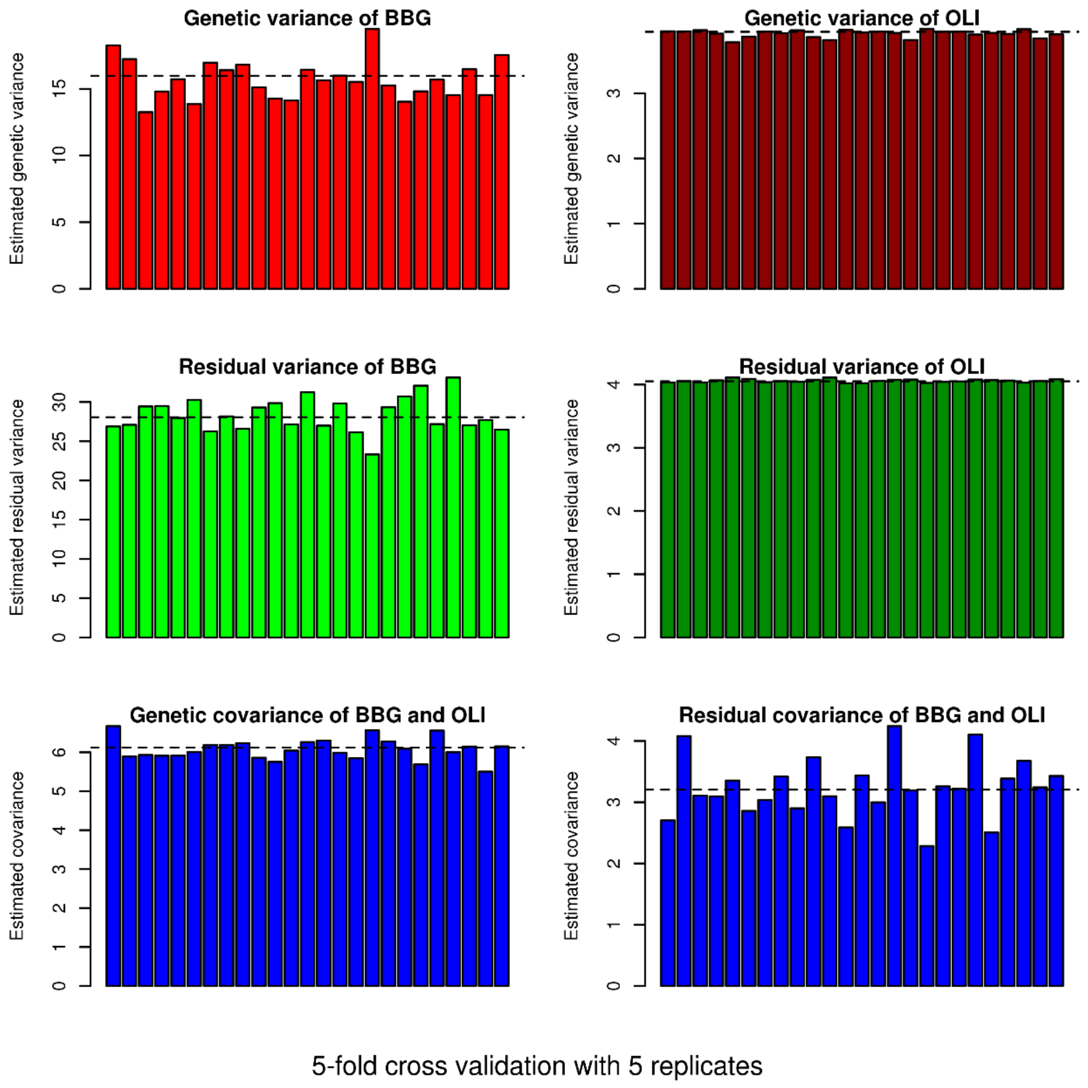

Fig. S1 Comparison of pre estimated genetic and residual variances and covariances of converged bivariate sERRBLUP model (top 5%) based on the full dataset (dashed horizontal lines) and estimated genetic and residual variances and covariances of converged bivariate sERRBLUP (top 5%) based on training set in each run of 5-fold cross validation with 5 replicates (colored bars) for predicting BBG when the additional environment is OLI in KE for trait PH_V4.

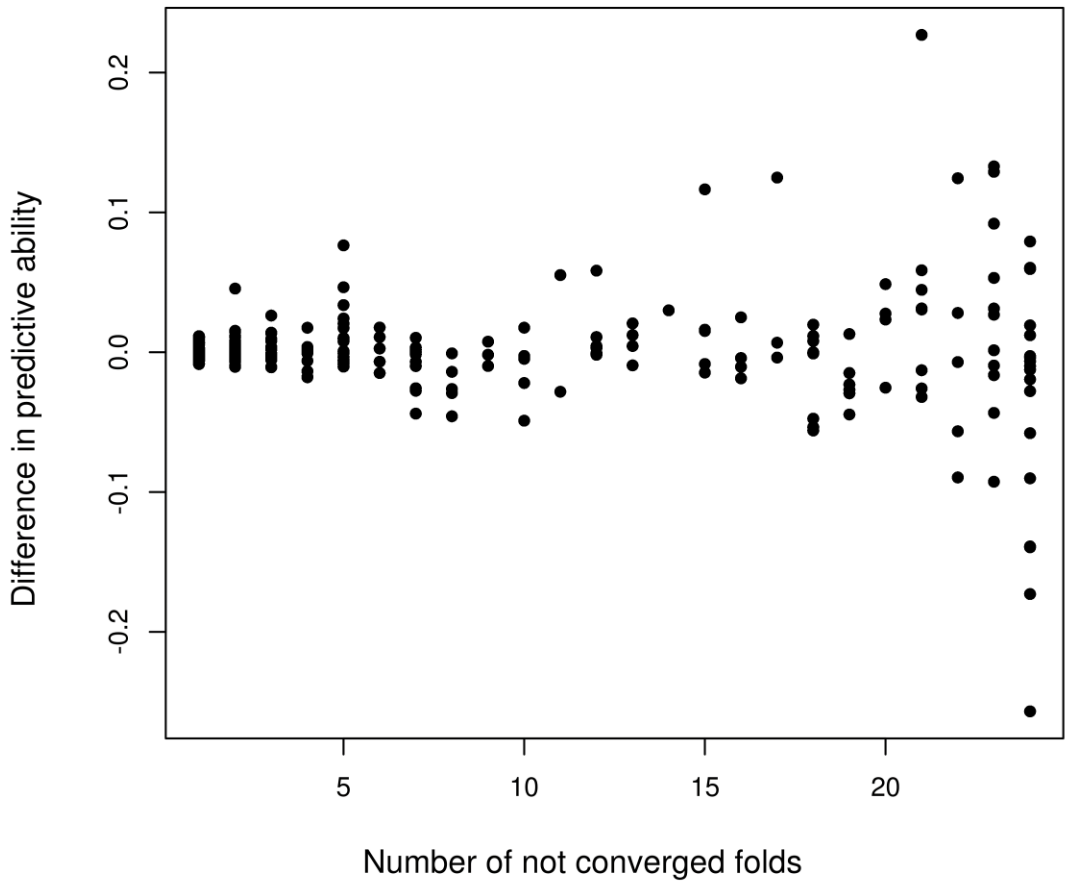

Fig. S2 The difference between the mean predictive ability of only the converged folds and the mean predictive ability of all folds in 5-fold cross validation with 5 replicates vs. the number of the folds (1 to 24) which did not converge across all traits in all combinations for both KE and PE.

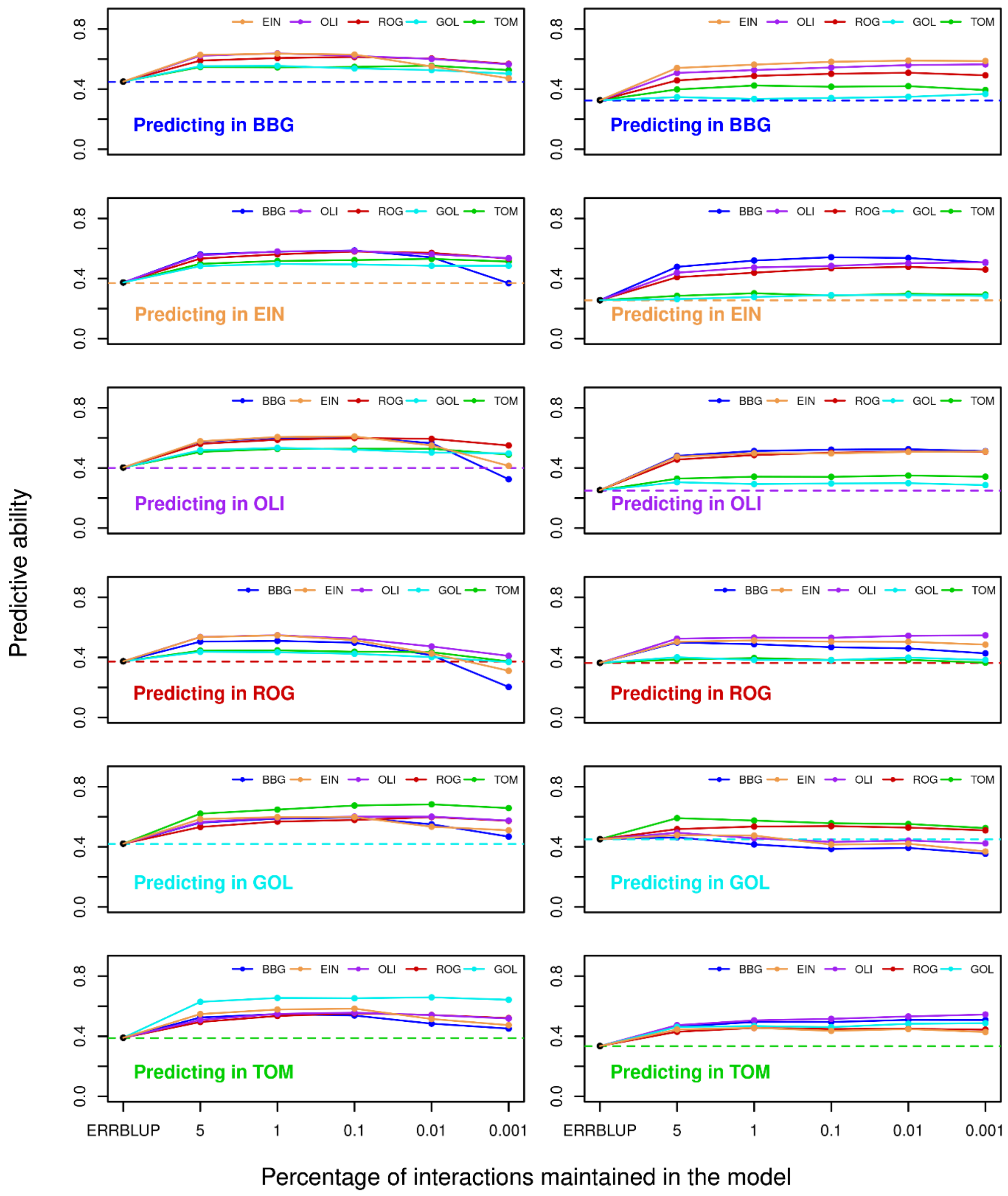

Fig. S3a Predictive ability for univariate GBLUP within environment (dashed horizontal line), univariate ERRBLUP within environment (black filled circle) and univariate sERRBLUP across environments when the SNP interaction selections are based on estimated effects variances (solid colored lines) for trait EV_V3 in KE (left side plots) and PE (right side plots). In each panel, the solid lines' color indicates the environment in which the relationship matrices determined by variable selection.

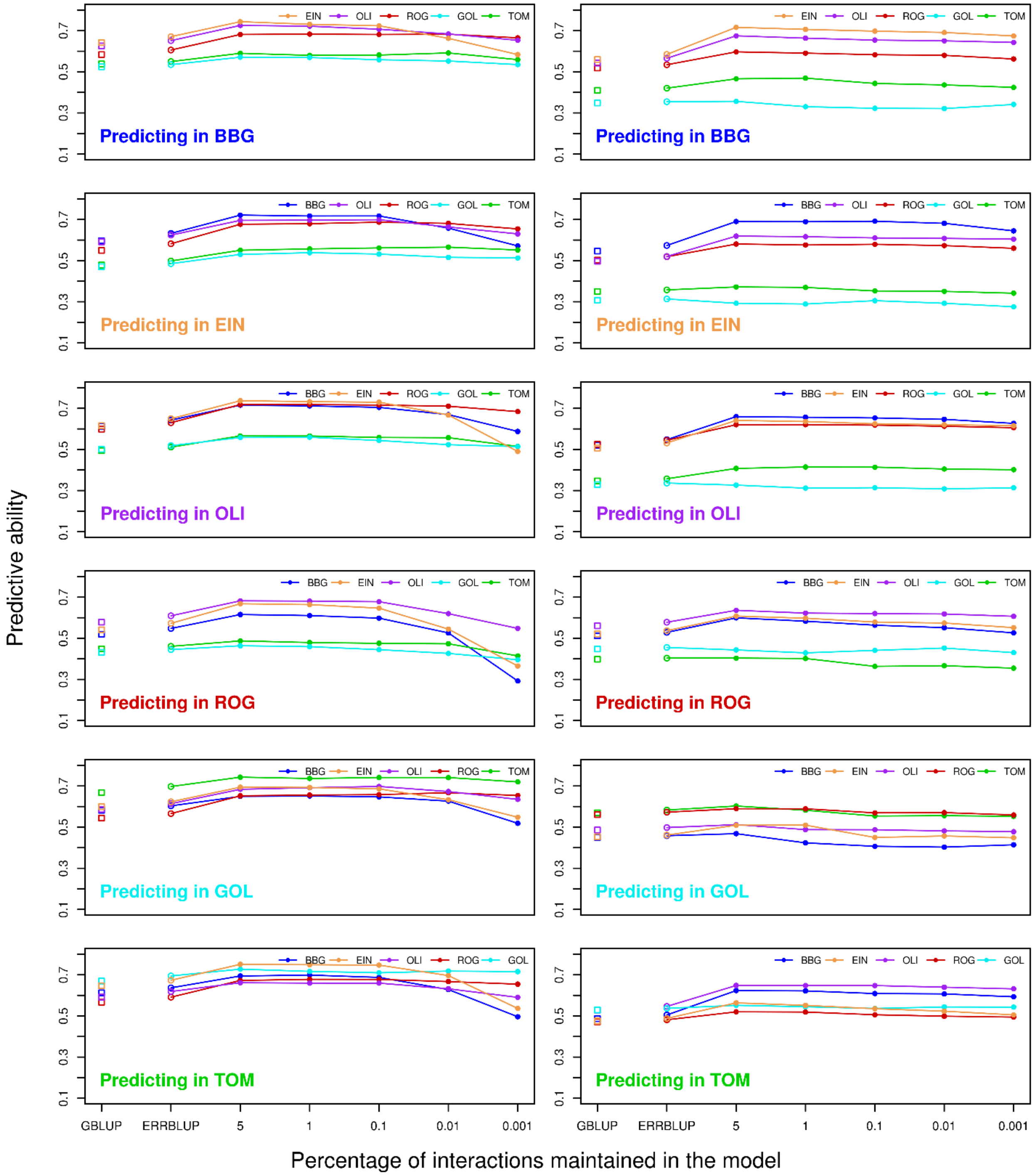

Fig. S3b Predictive ability for bivariate GBLUP (open squares), bivariate ERRBLUP (open circles) and bivariate sERRBLUP (filled circles and solid lines) when SNP interaction selections are based on estimated effects variances in KE (left side) and PE (right side) for trait EV-V3. In each panel, the solid lines' color indicates the additional environment used to predict the target environment.

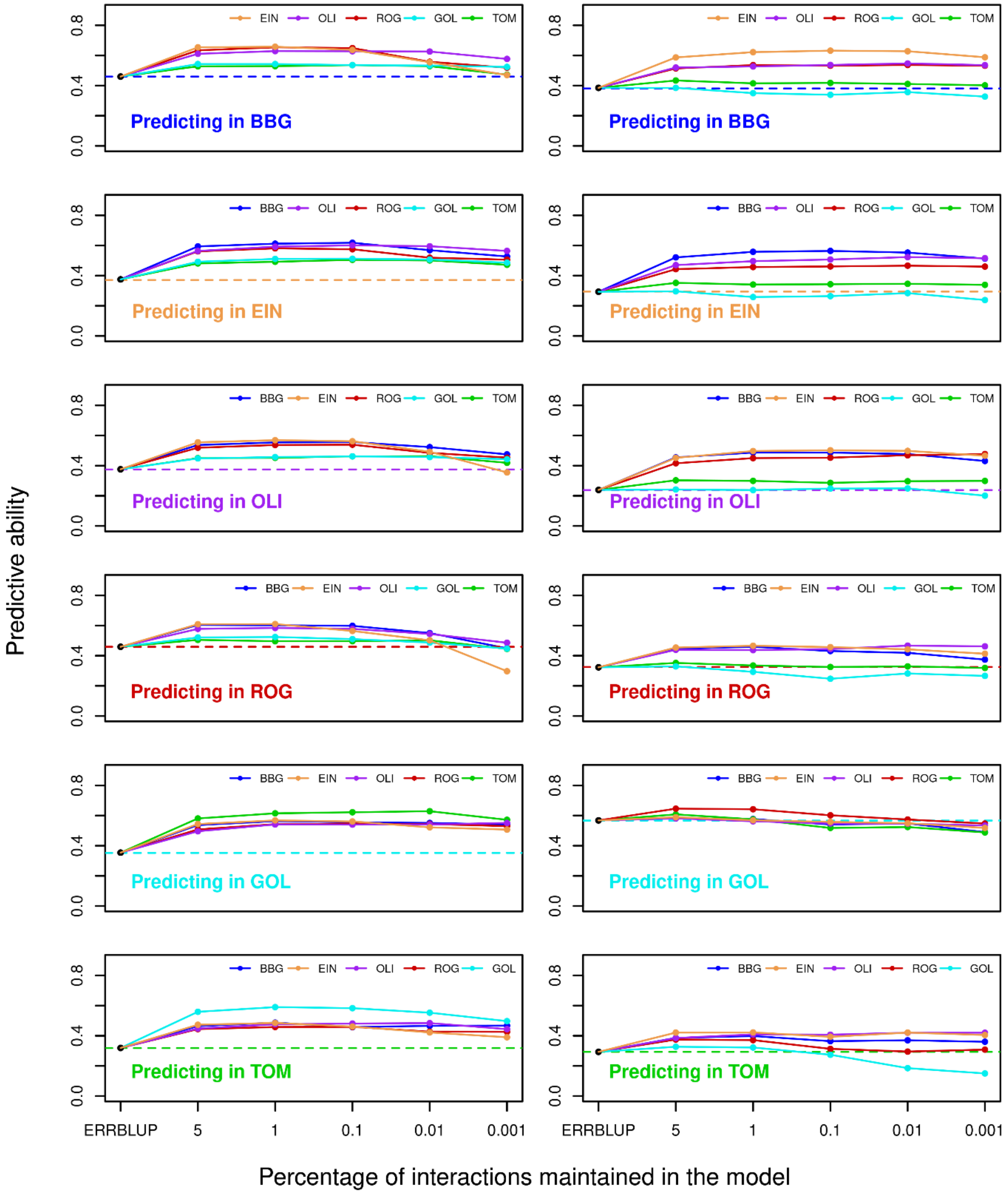

Fig. S4a Predictive ability for univariate GBLUP within environment (dashed horizontal line), univariate ERRBLUP within environment (black filled circle) and univariate sERRBLUP across environments when the SNP interaction selections are based on estimated effects variances (solid colored lines) for trait EV_V4 in KE (left side plots) and PE (right side plots). In each panel, the solid lines' color indicates the environment in which the relationship matrices determined by variable selection.

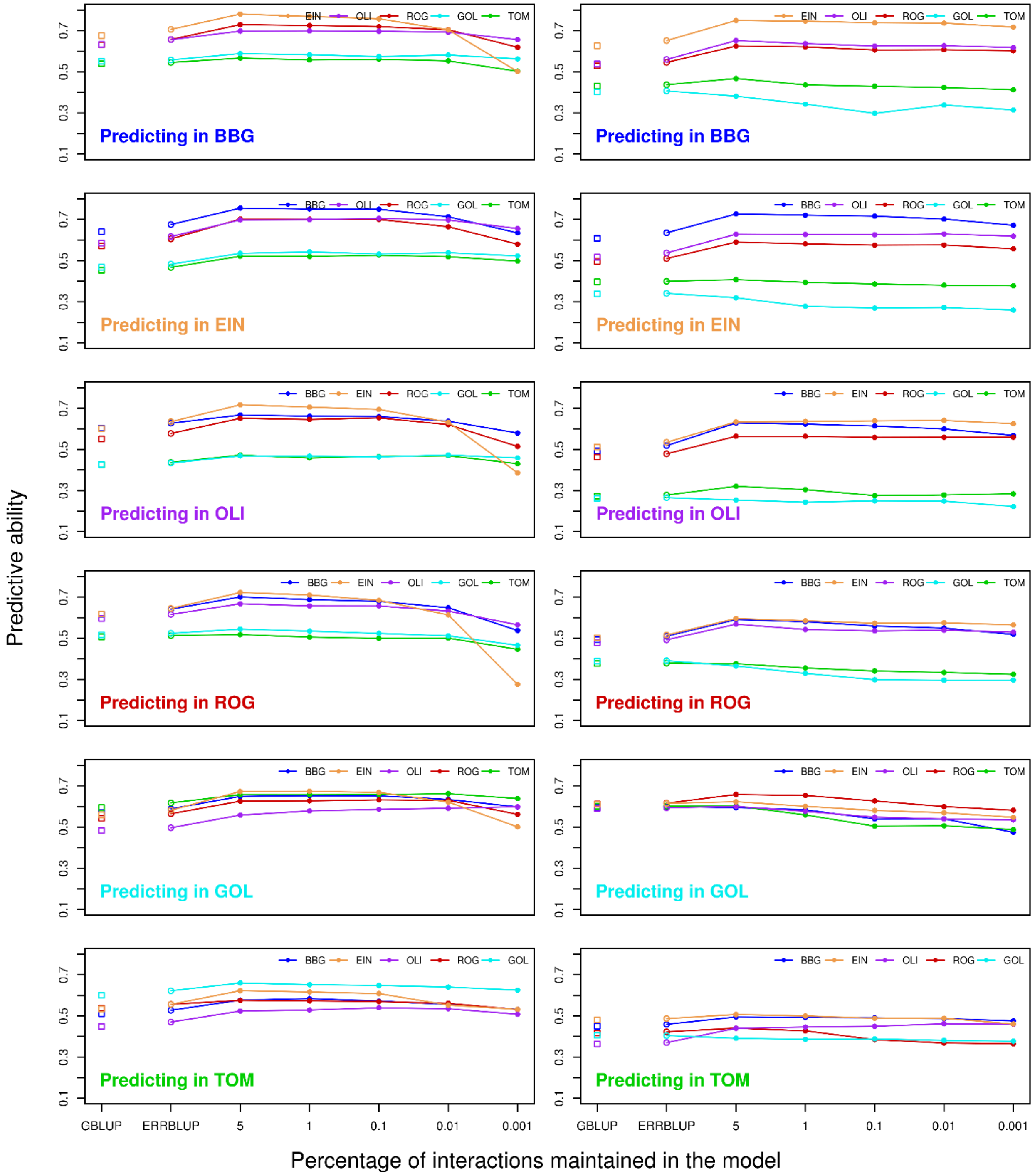

Fig. S4b Predictive ability for bivariate GBLUP (open squares), bivariate ERRBLUP (open circles) and bivariate sERRBLUP (filled circles and solid lines) when SNP interaction selections are based on estimated effects variances in KE (left side) and PE (right side) for trait EV-V4. In each panel, the solid lines' color indicates the additional environment used to predict the target environment.

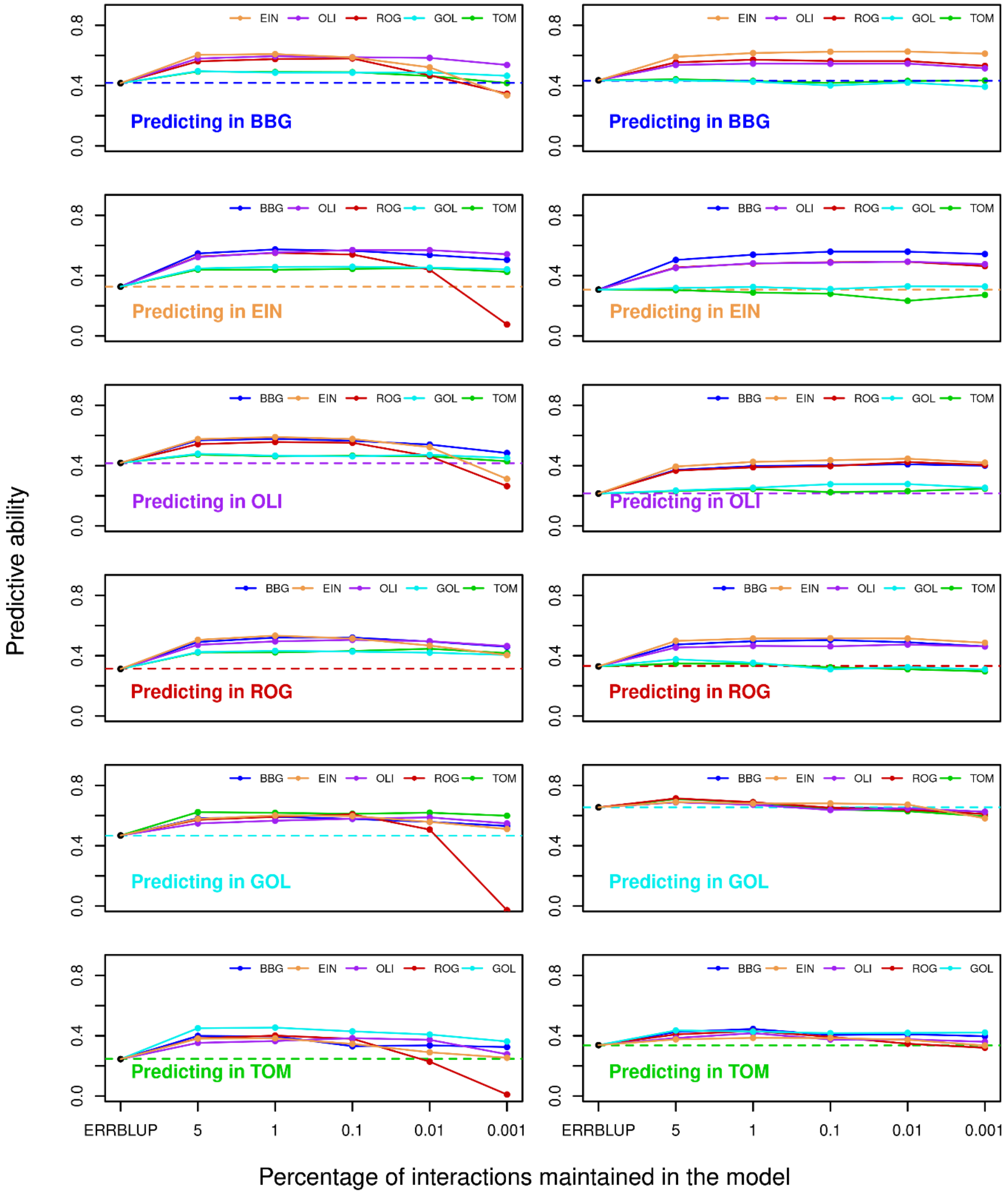

Fig. S5a Predictive ability for univariate GBLUP within environment (dashed horizontal line), univariate ERRBLUP within environment (black filled circle) and univariate sERRBLUP across environments when the SNP interaction selections are based on estimated effects variances (solid colored lines) for trait EV_V6 in KE (left side plots) and PE (right side plots). In each panel, the solid lines' color indicates the environment in which the relationship matrices determined by variable selection.

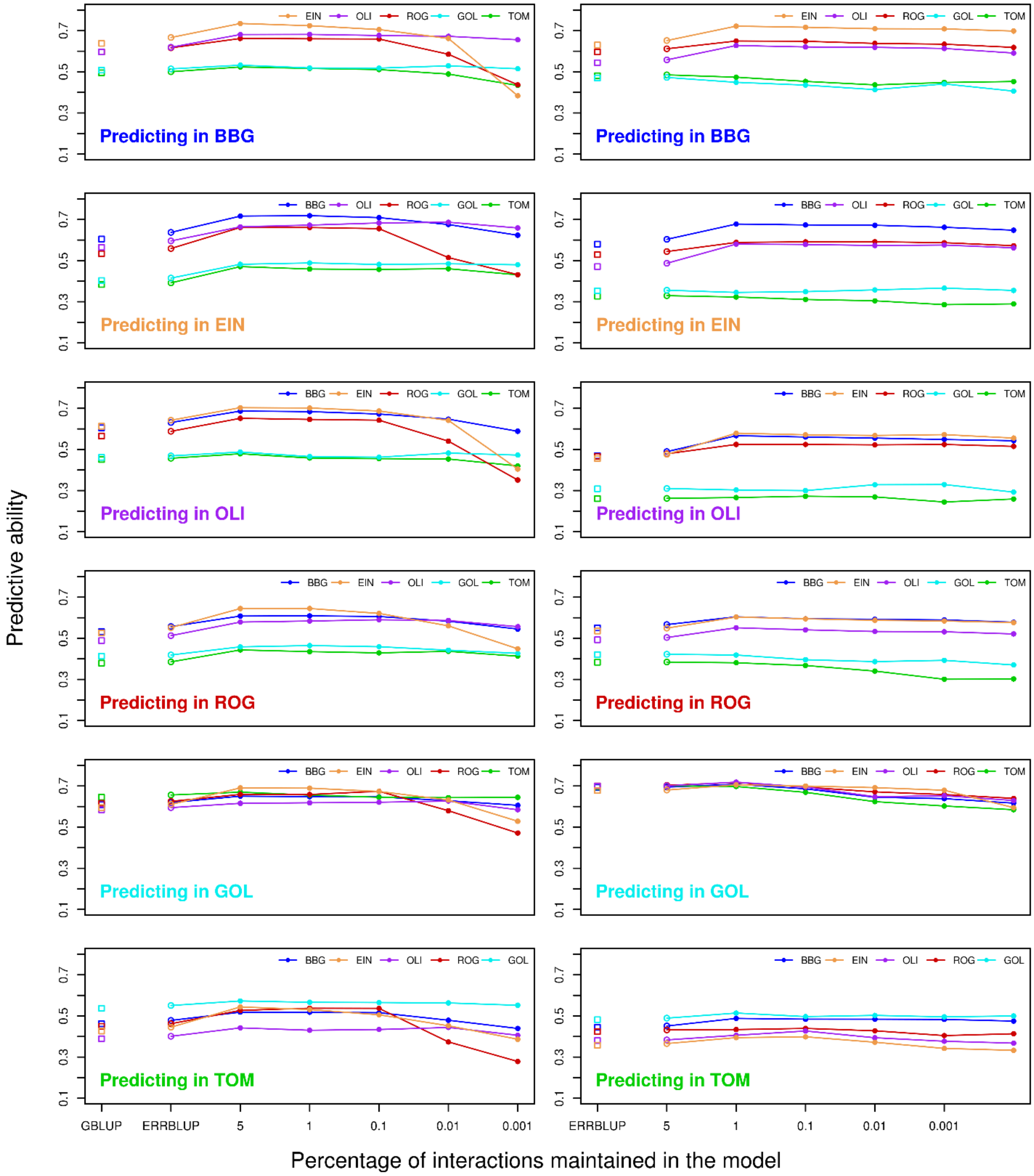

Fig. S5b Predictive ability for bivariate GBLUP (open squares), bivariate ERRBLUP (open circles) and bivariate sERRBLUP (filled circles and solid lines) when SNP interaction selections are based on estimated effects variances in KE (left side) and PE (right side) for trait EV-V6. In each panel, the solid lines' color indicates the additional environment used to predict the target environment.

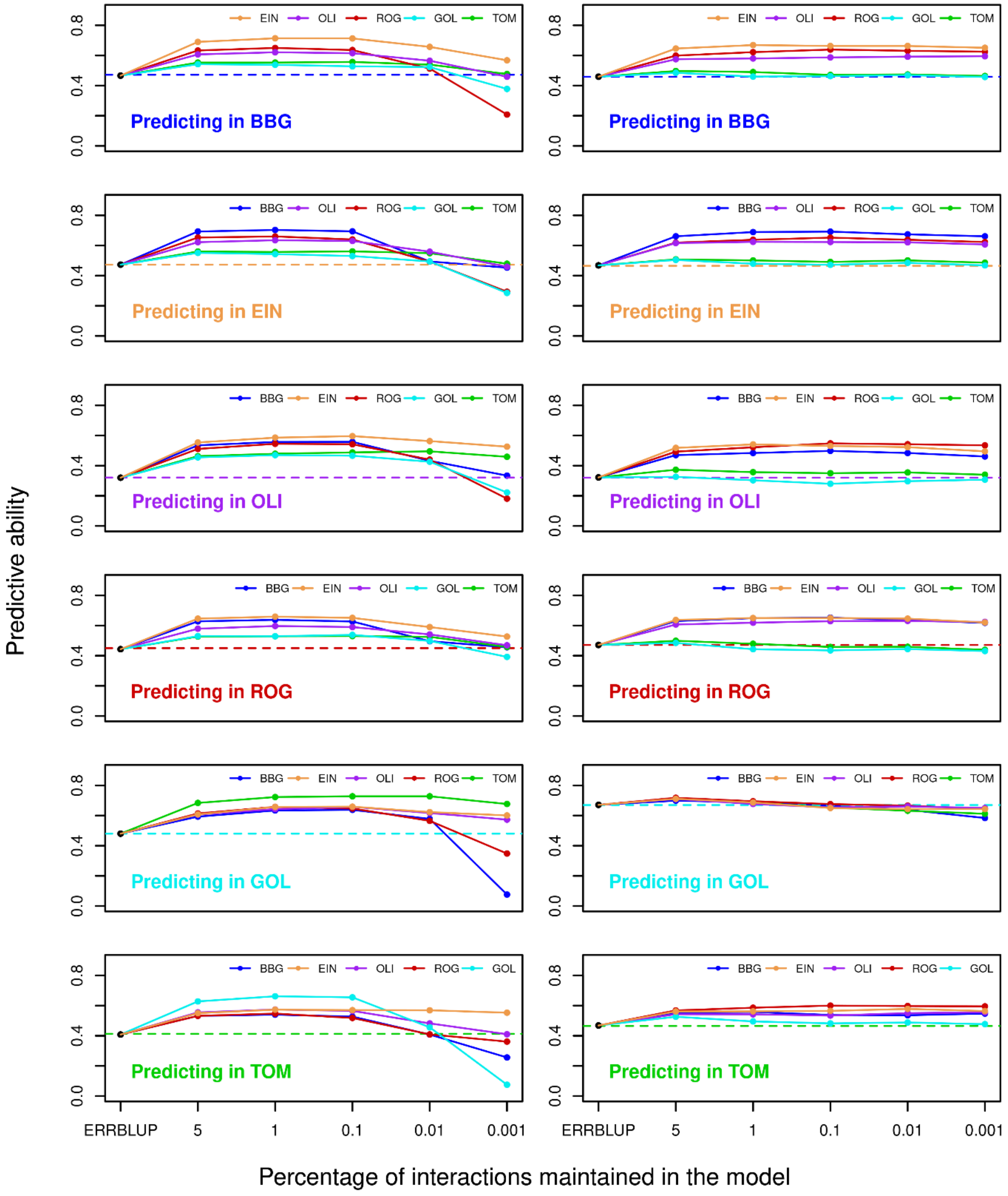

Fig. S6a Predictive ability for univariate GBLUP within environment (dashed horizontal line), univariate ERRBLUP within environment (black filled circle) and univariate sERRBLUP across environments when the SNP interaction selections are based on estimated effects variances (solid colored lines) for trait PH-V6 in KE (left side plots) and PE (right side plots). In each panel, the solid lines' color indicates the environment in which the relationship matrices determined by variable selection.

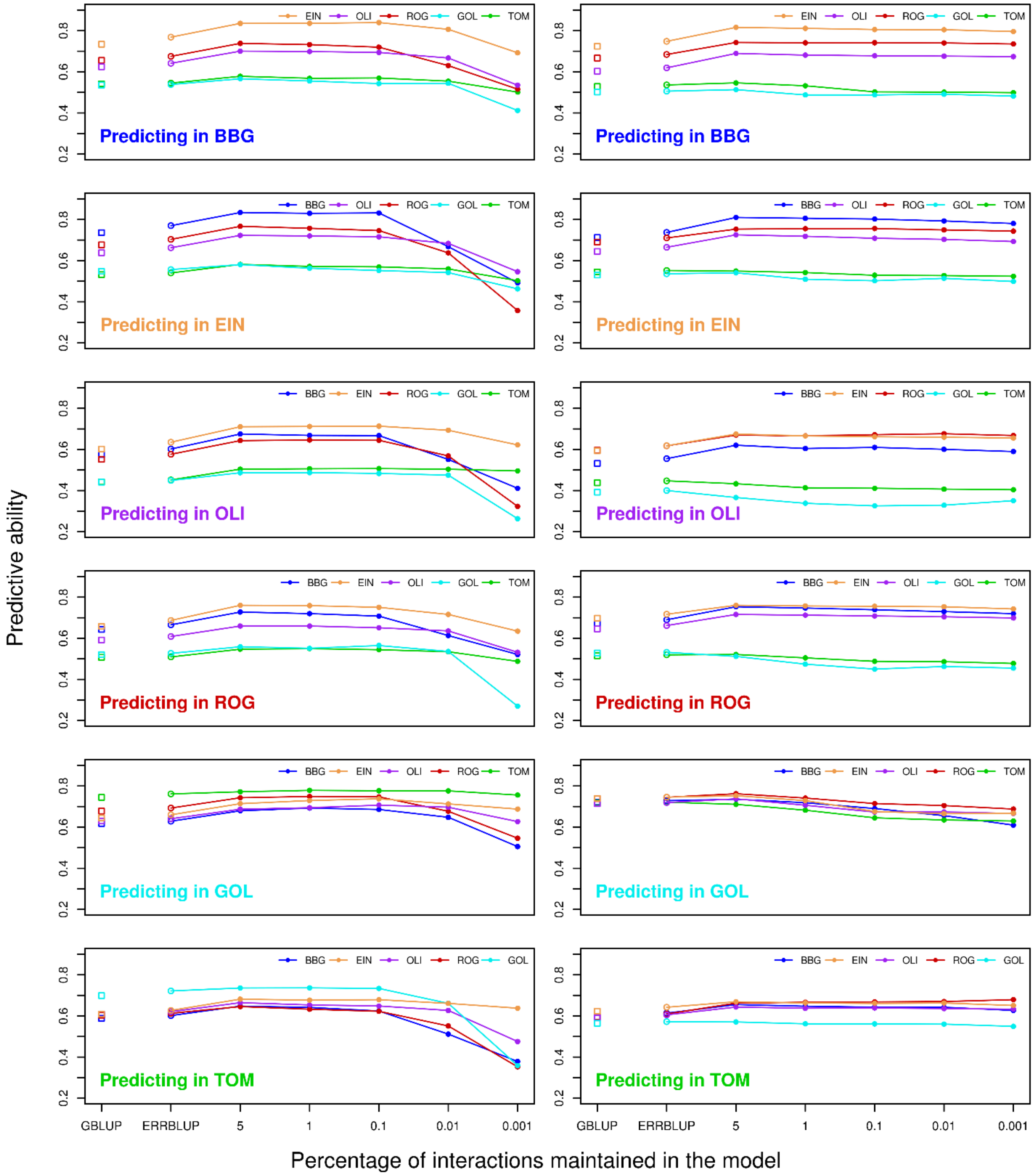

Fig. S6b Predictive ability for bivariate GBLUP (open squares), bivariate ERRBLUP (open circles) and bivariate sERRBLUP (filled circles and solid lines) when SNP interaction selections are based on estimated effects variances in KE (left side) and PE (right side) for trait PH-V6. In each panel, the solid lines' color indicates the additional environment used to predict the target environment.

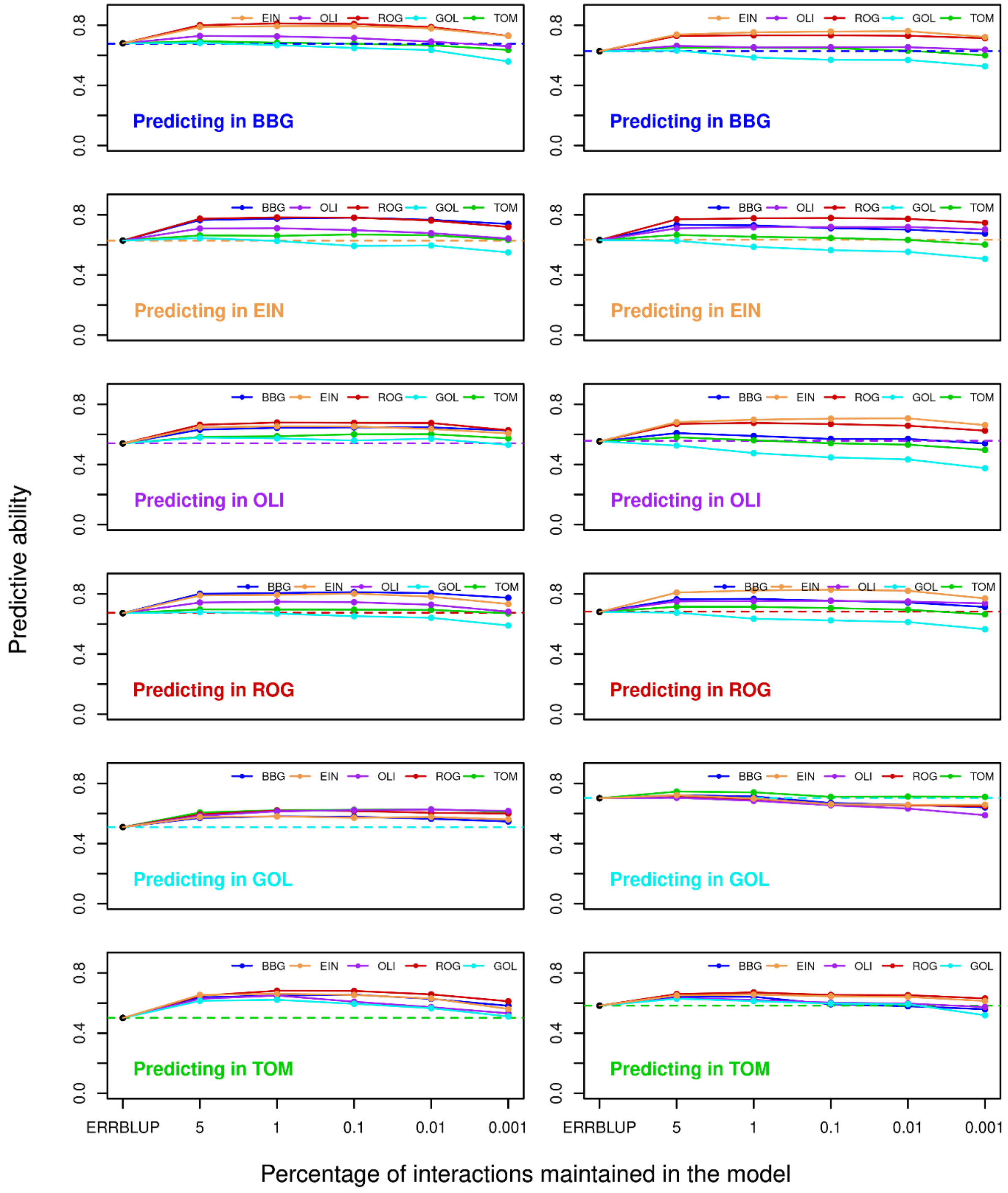

Fig. S7a Predictive ability for univariate GBLUP within environment (dashed horizontal line), univariate ERRBLUP within environment (black filled circle) and univariate sERRBLUP across environments when the SNP interaction selections are based on estimated effects variances (solid colored lines) for trait PH-final in KE (left side plots) and PE (right side plots). In each panel, the solid lines' color indicates the environment in which the relationship matrices determined by variable selection.

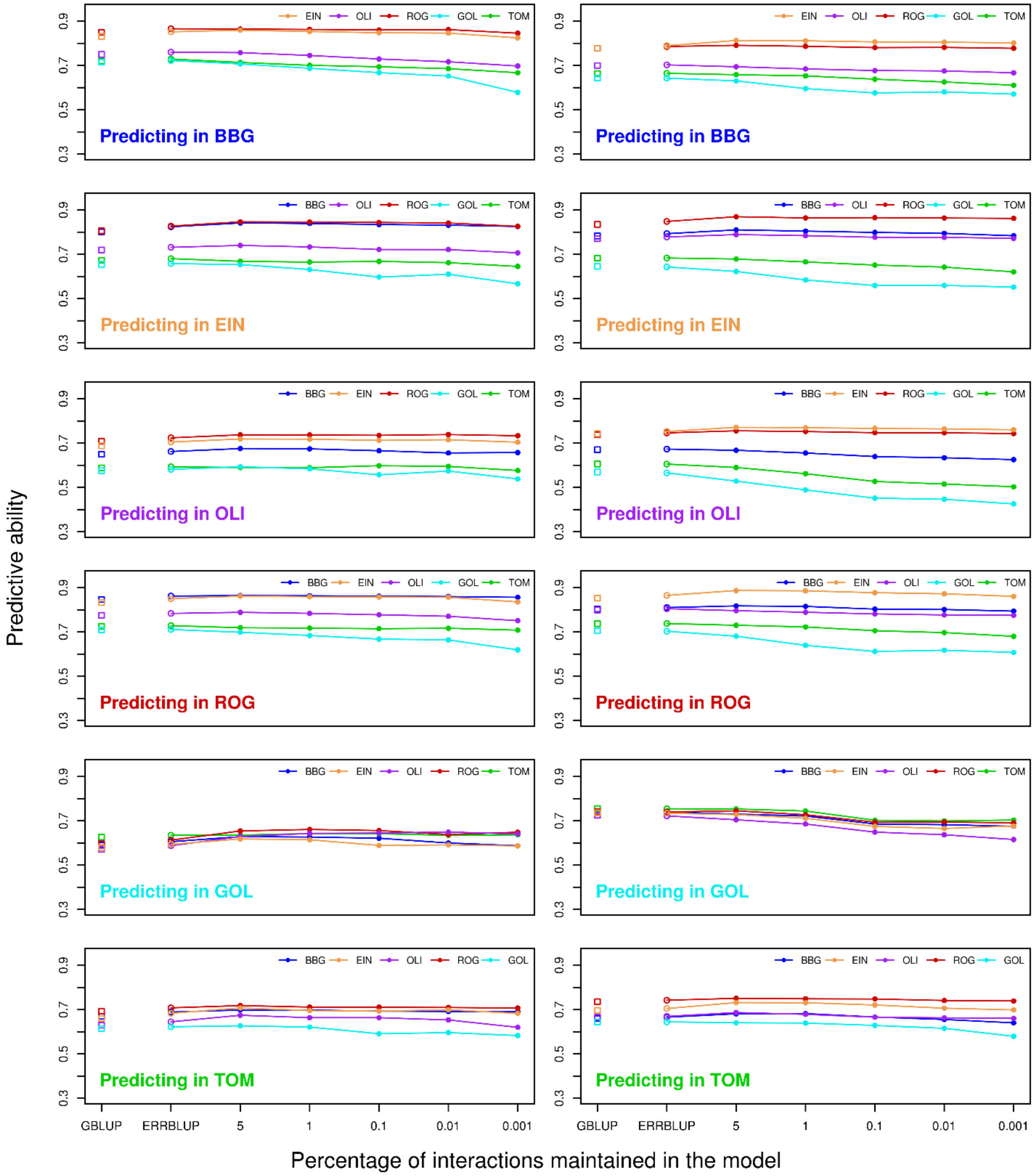

Fig. S7b Predictive ability for bivariate GBLUP (open squares), bivariate ERRBLUP (open circles) and bivariate sERRBLUP (filled circles and solid lines) when SNP interaction selections are based on estimated effects variances in KE (left side) and PE (right side) for trait PH-final. In each panel, the solid lines' color indicates the additional environment used to predict the target environment.

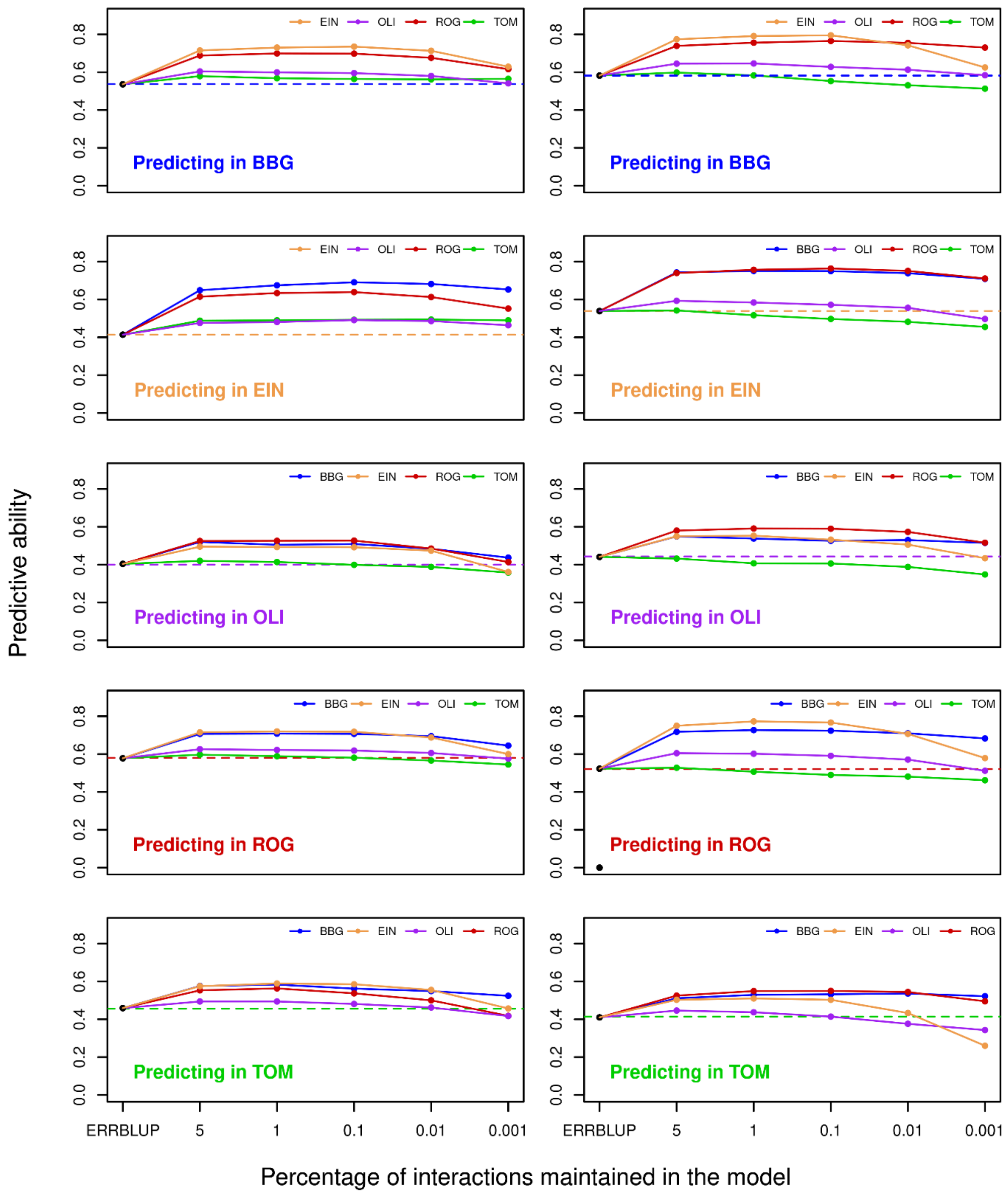

Fig. S8a Predictive ability for univariate GBLUP within environment (dashed horizontal line), univariate ERRBLUP within environment (black filled circle) and univariate sERRBLUP across environments when the SNP interaction selections are based on estimated effects variances (solid colored lines) for trait FF in KE (left side plots) and PE (right side plots). In each panel, the solid lines' color indicates the environment in which the relationship matrices determined by variable selection.

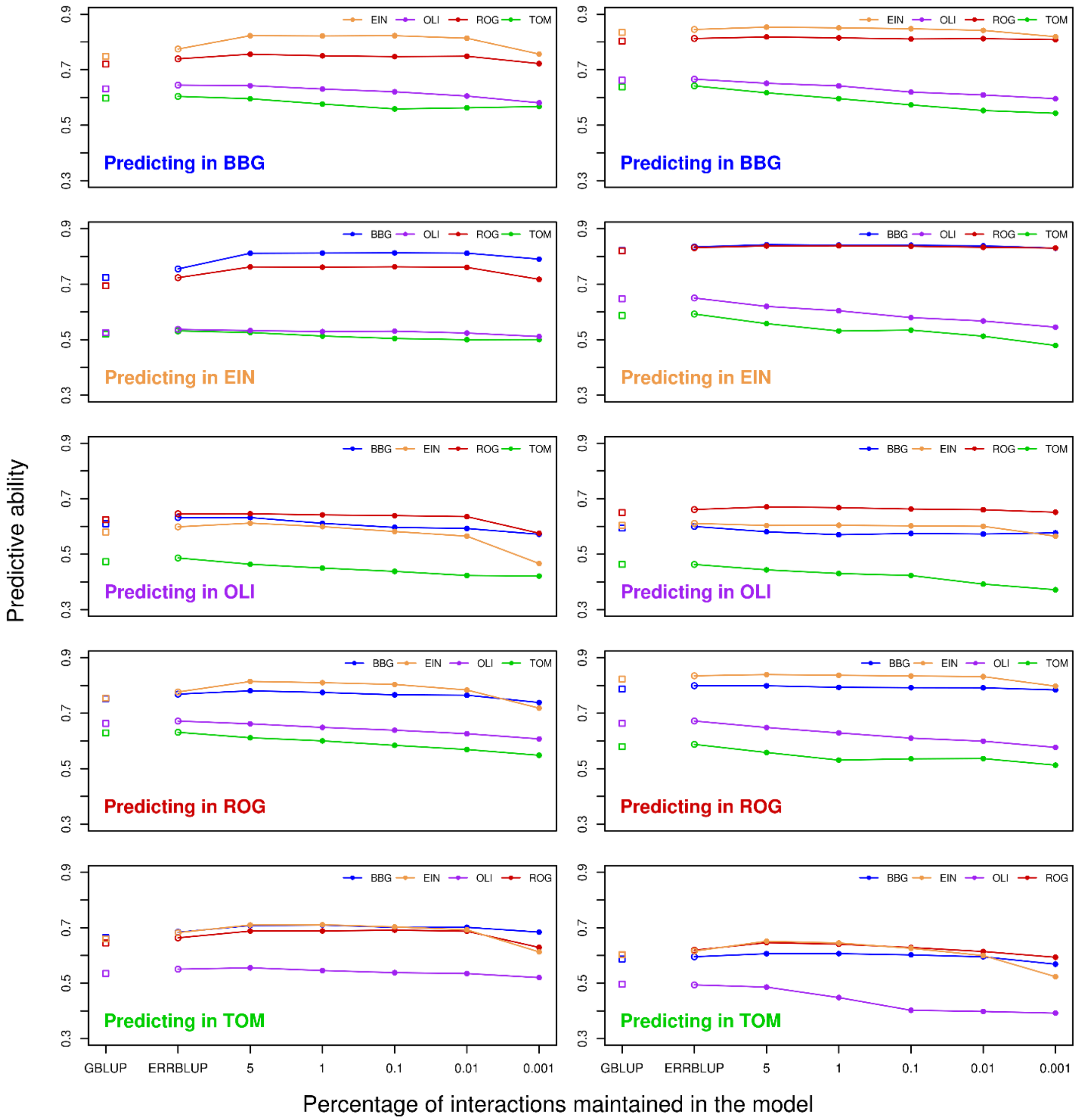

Fig. S8b Predictive ability for bivariate GBLUP (open squares), bivariate ERRBLUP (open circles) and bivariate sERRBLUP (filled circles and solid lines) when SNP interaction selections are based on estimated effects variances in KE (left side) and PE (right side) for trait FF. In each panel, the solid lines' color indicates the additional environment used to predict the target environment.

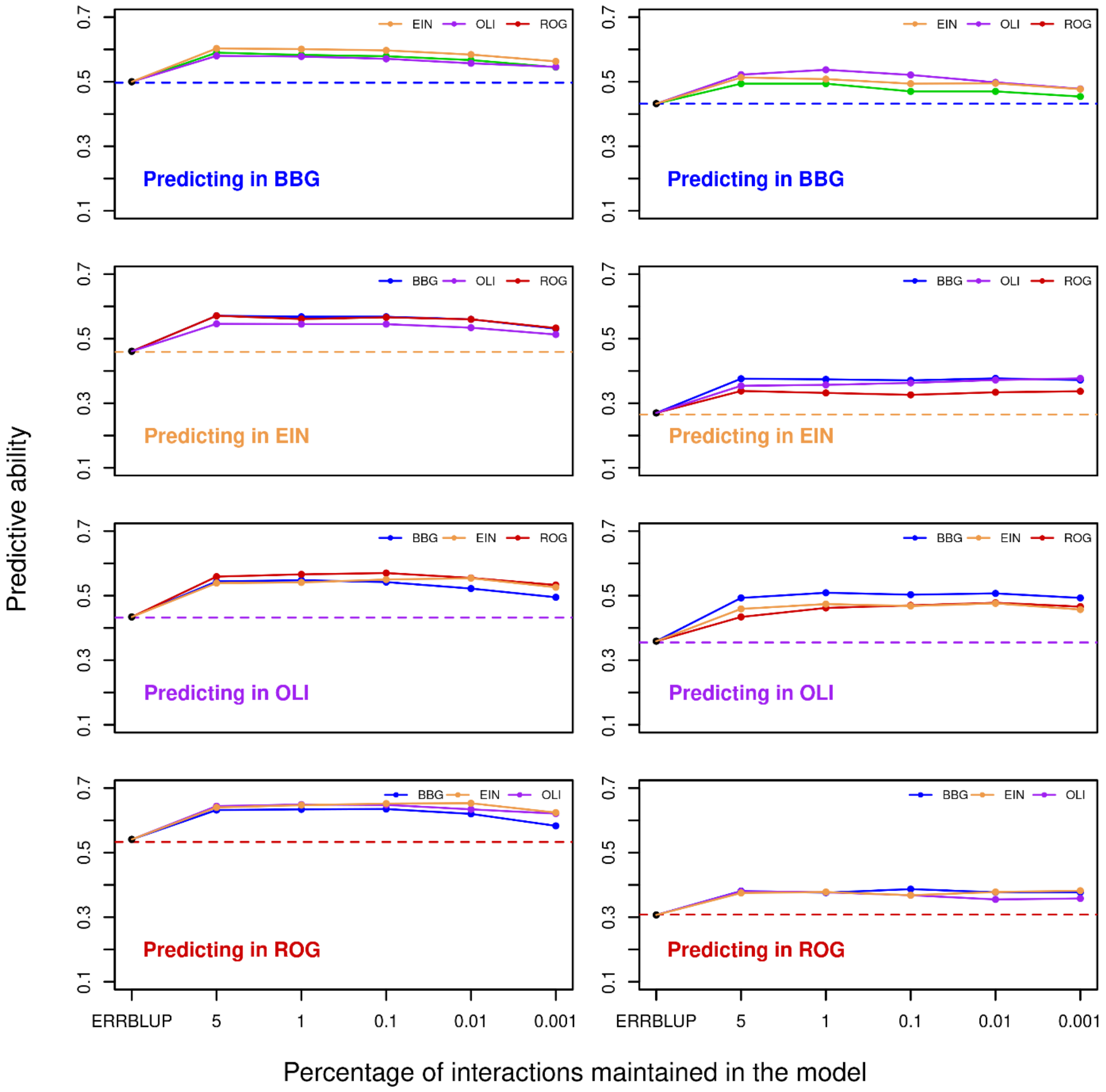

Fig. S9a Predictive ability for univariate GBLUP within environment (dashed horizontal line), univariate ERRBLUP within environment (black filled circle) and univariate sERRBLUP across environments when the SNP interaction selections are based on estimated effects variances (solid colored lines) for trait RL in KE (left side plots) and PE (right side plots). In each panel, the solid lines' color indicates the environment in which the relationship matrices determined by variable selection.

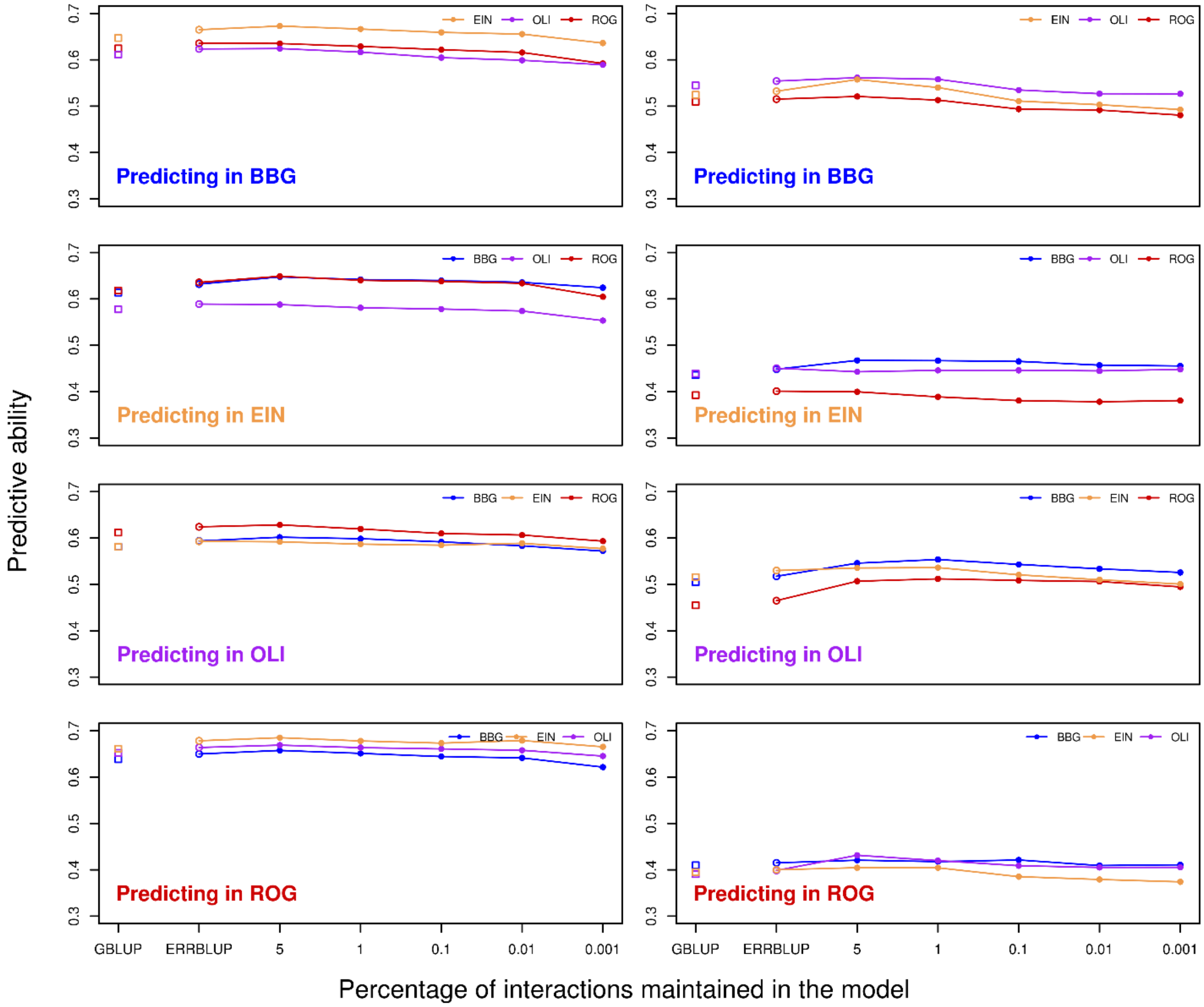

Fig. S9b Predictive ability for bivariate GBLUP (open squares), bivariate ERRBLUP (open circles) and bivariate sERRBLUP (filled circles and solid lines) when SNP interaction selections are based on estimated effects variances in KE (left side) and PE (right side) for trait RL. In each panel, the solid lines' color indicates the additional environment used to predict the target environment

.

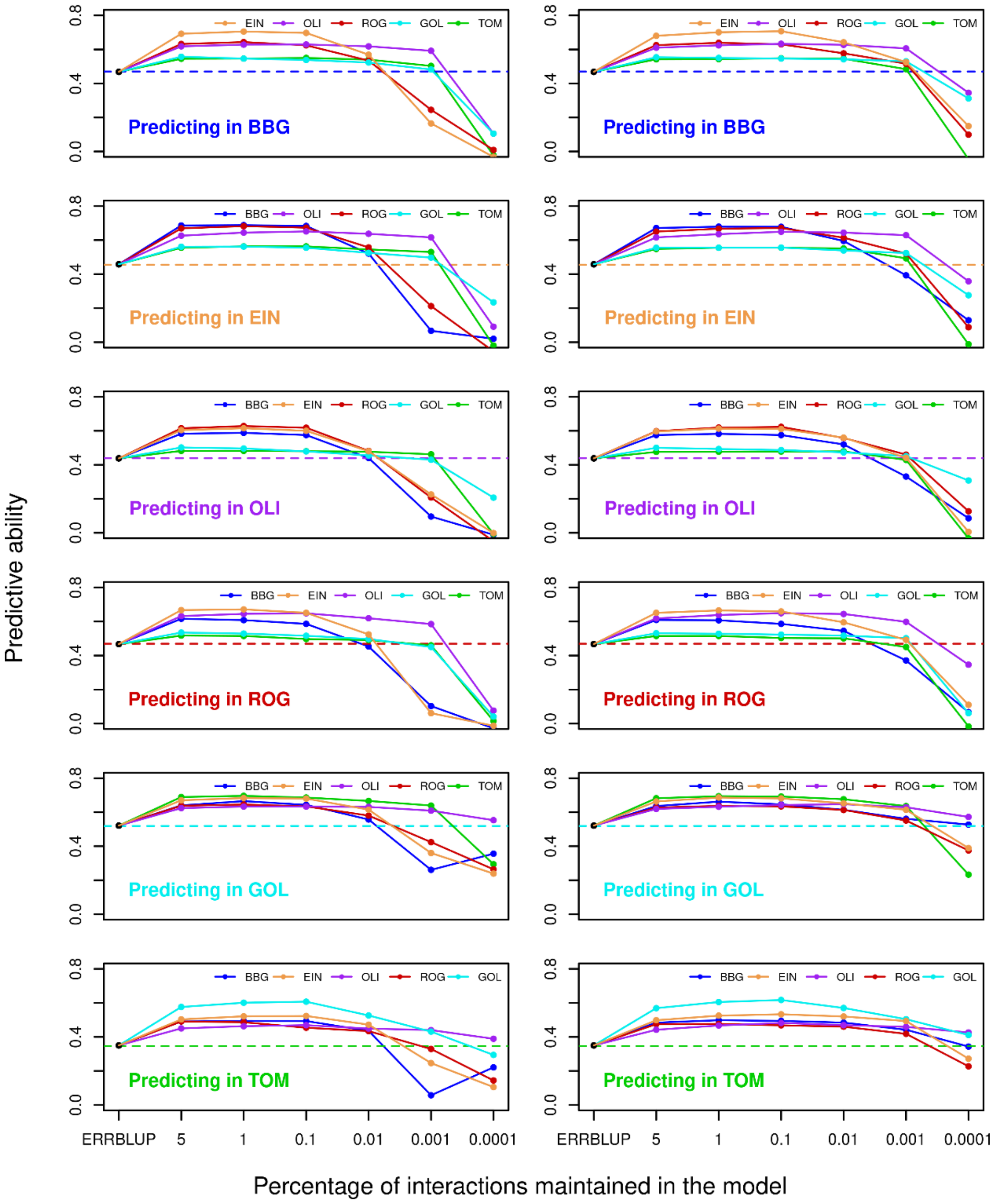

Fig. S10 Predictive ability for univariate GBLUP within environment (dashed horizontal line), univariate ERRBLUP within environment (black filled circle) and univariate sERRBLUP across environments (solid colored lines) when SNP interaction selections are based on estimated effects sizes (left side) and estimated effects variances (right side) for trait PH-V4 in KE. In each panel, the solid lines' color indicates the environment in which the relationship matrices were determined by variable selection.

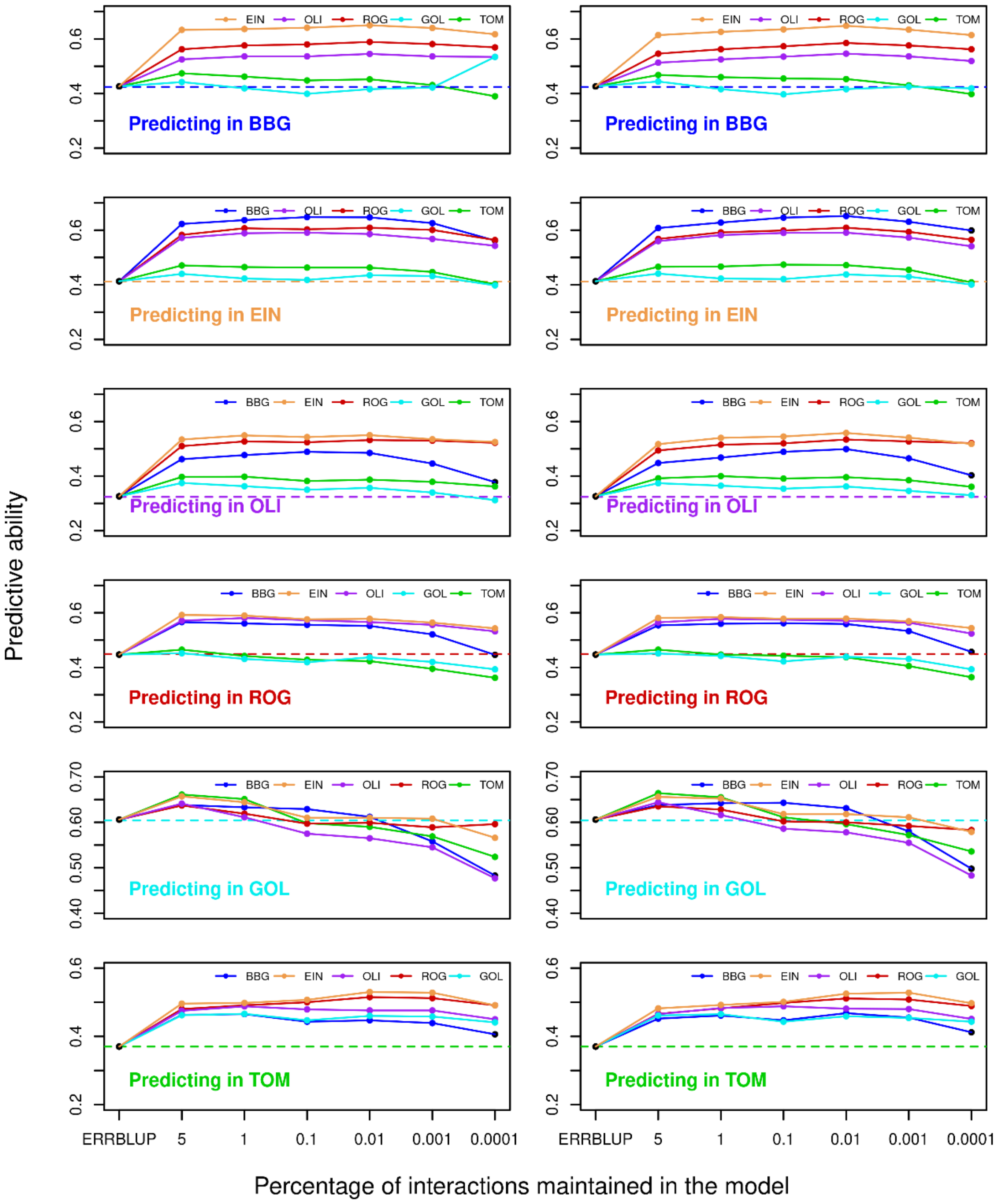

Fig. S11 Predictive ability for univariate GBLUP within environment (dashed horizontal line), univariate ERRBLUP within environment (black filled circle) and univariate sERRBLUP across environments (solid colored lines) when SNP interaction selections are based on estimated effects sizes (left side) and estimated effects variances (right side) for trait PH-V4 in PE. In each panel, the solid lines' color indicates the environment in which the relationship matrices were determined by variable selection.

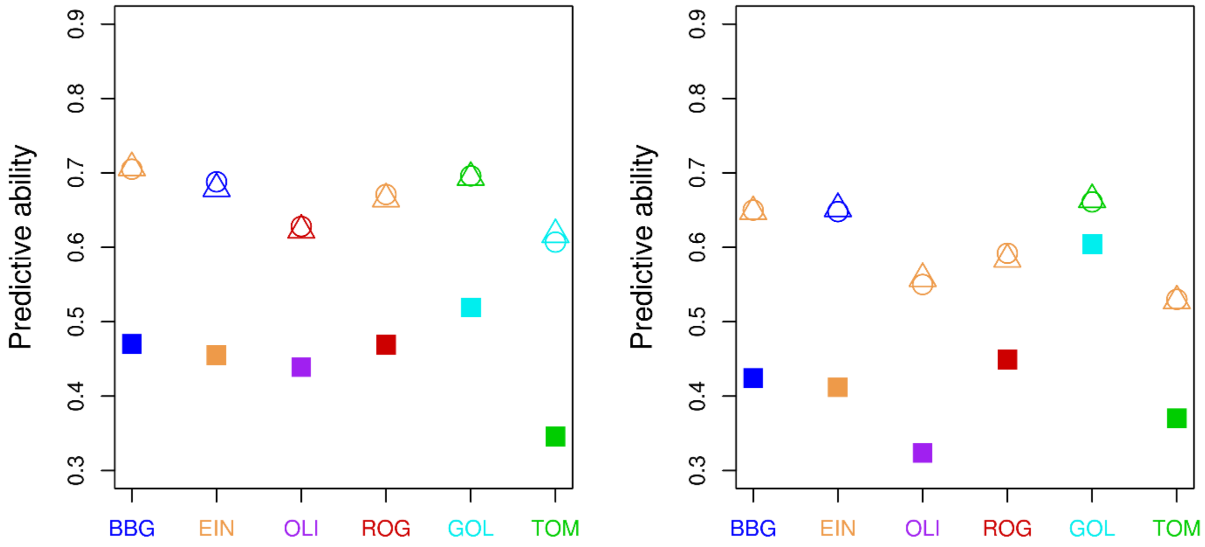

Fig. S12 Comparison of predictive ability of univariate GBLUP within environments (filled squares) and the maximum predictive ability of univariate sERRBLUP across environments when the SNP interaction selections are based on estimated effects sizes (circles) and estimated effects variances (triangles) for trait PH-V4 in KE (left side plot) and in PE (right side plot). The colors dark blue, orange, purple, red, light blue and green represent the environments BBG, EIN, OLI, ROG, GOL and TOM, respectively. The circles' and triangles' colors indicate the environment which had the maximum predictive ability for this respective target environment.

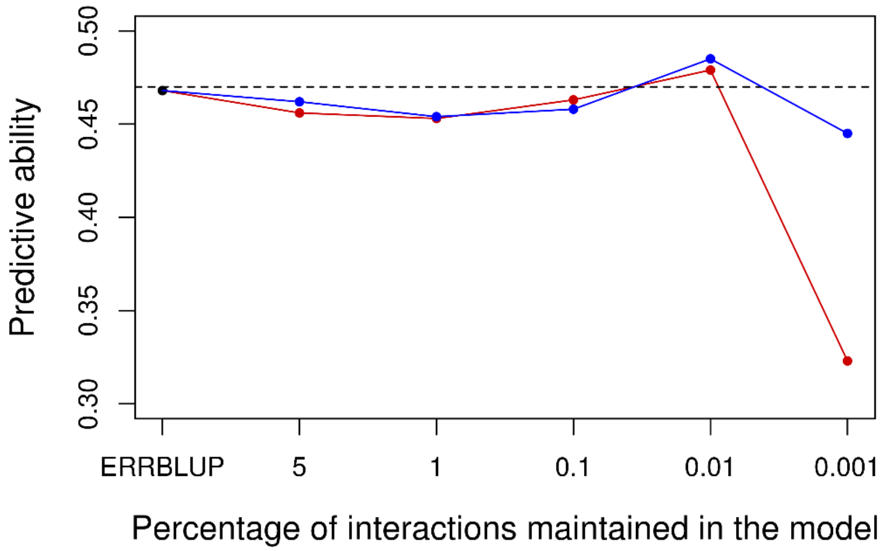

Fig. S13 Predictive ability for univariate GBLUP within Bernburg (dashed horizontal line), univariate ERRBLUP within Bernburg (black filled circle) and univariate sERRBLUP when the SNP interaction selections are based on estimated effects variances (blue solid line) and estimated effect sizes (red solid line) within Bernburg for trait PH-V4 in KE.

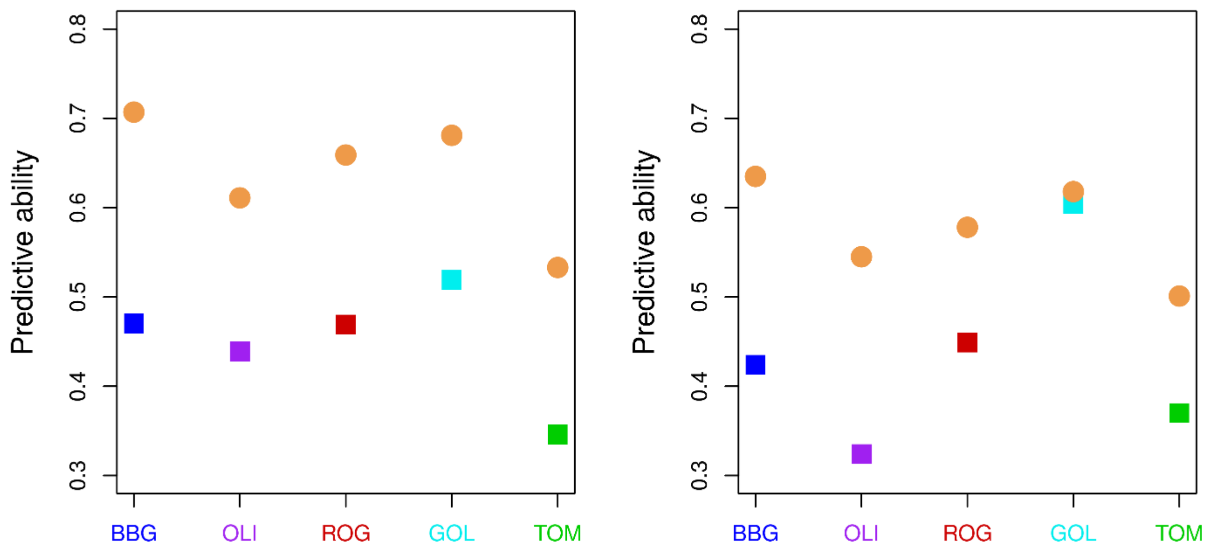

Fig. S14 Comparison of predictive ability of univariate GBLUP within environments (filled squares) and the maximum predictive ability of univariate sERRBLUP across EIN when the SNP interaction selections are based on estimated effects variances (orange circles) for trait PH-V4 in KE (left side plot) and in PE (right side plot). The colors dark blue, purple, red, light blue and green represent the environments BBG, OLI, ROG, GOL and TOM, respectively.

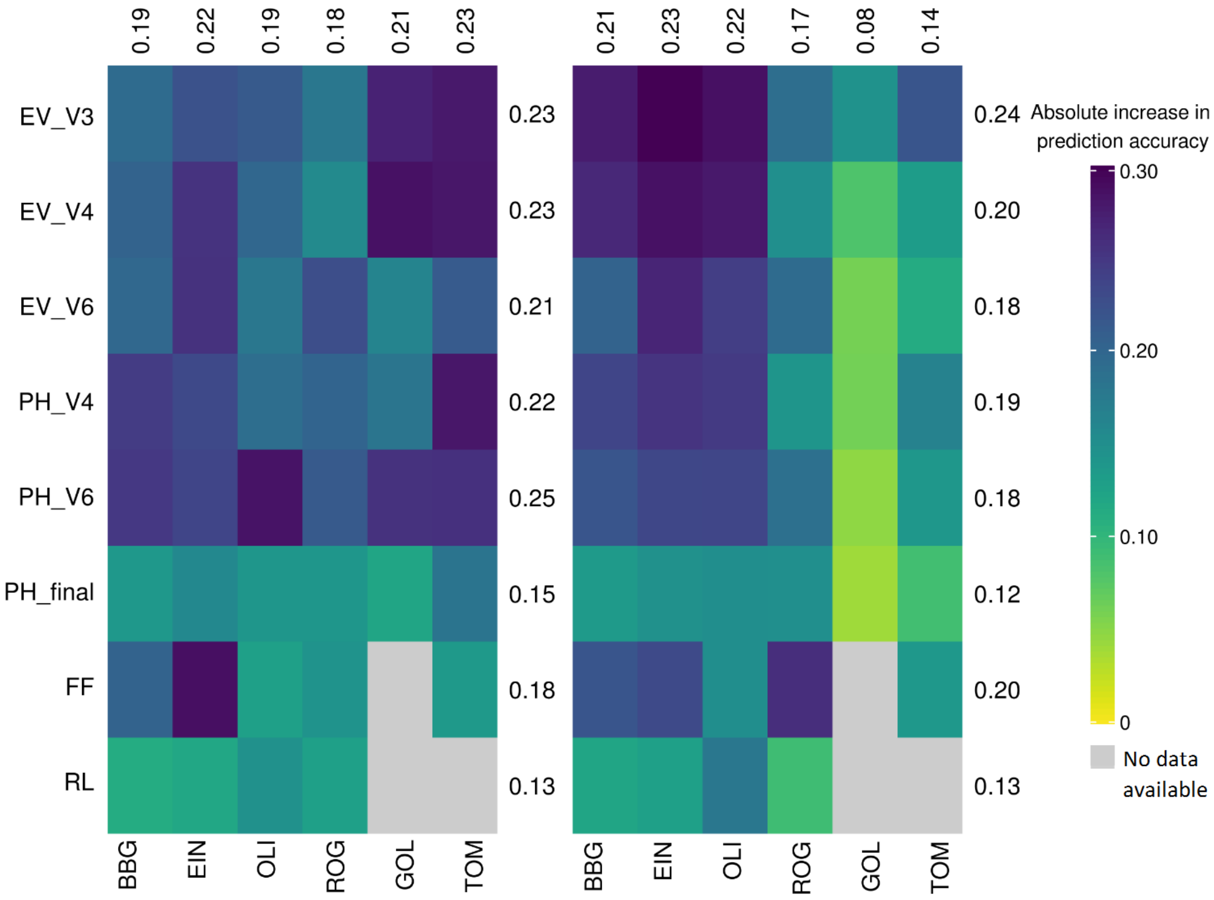

Fig. S15a Absolute increase in prediction accuracy from univariate GBLUP within environments to the maximum prediction accuracy of univariate sERRBLUP across environments when the SNP interaction selections are based on estimated effects variances in KE (left side plot) and in PE (right side plot). The average of absolute increase in prediction accuracy for each trait and environments are display in rows and columns, respectively.

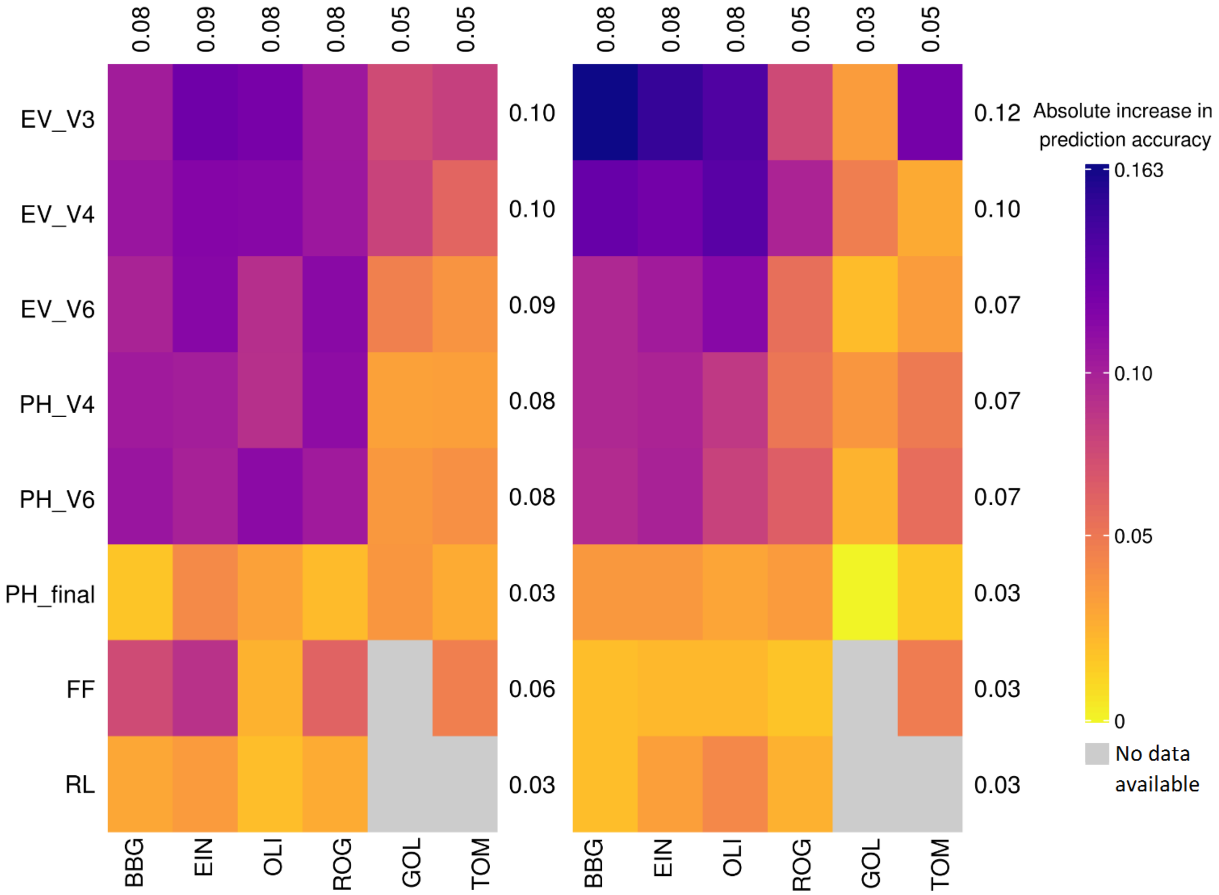

Fig. S15b Absolute increase in prediction accuracy from maximum bivariate GBLUP the maximum prediction accuracy of bivariate sERRBLUP when the SNP interaction selections are based on estimated effects variances in KE (left side plot) and in PE (right side plot). The average of absolute increase in prediction accuracy for each trait and environments are display in rows and columns, respectively.

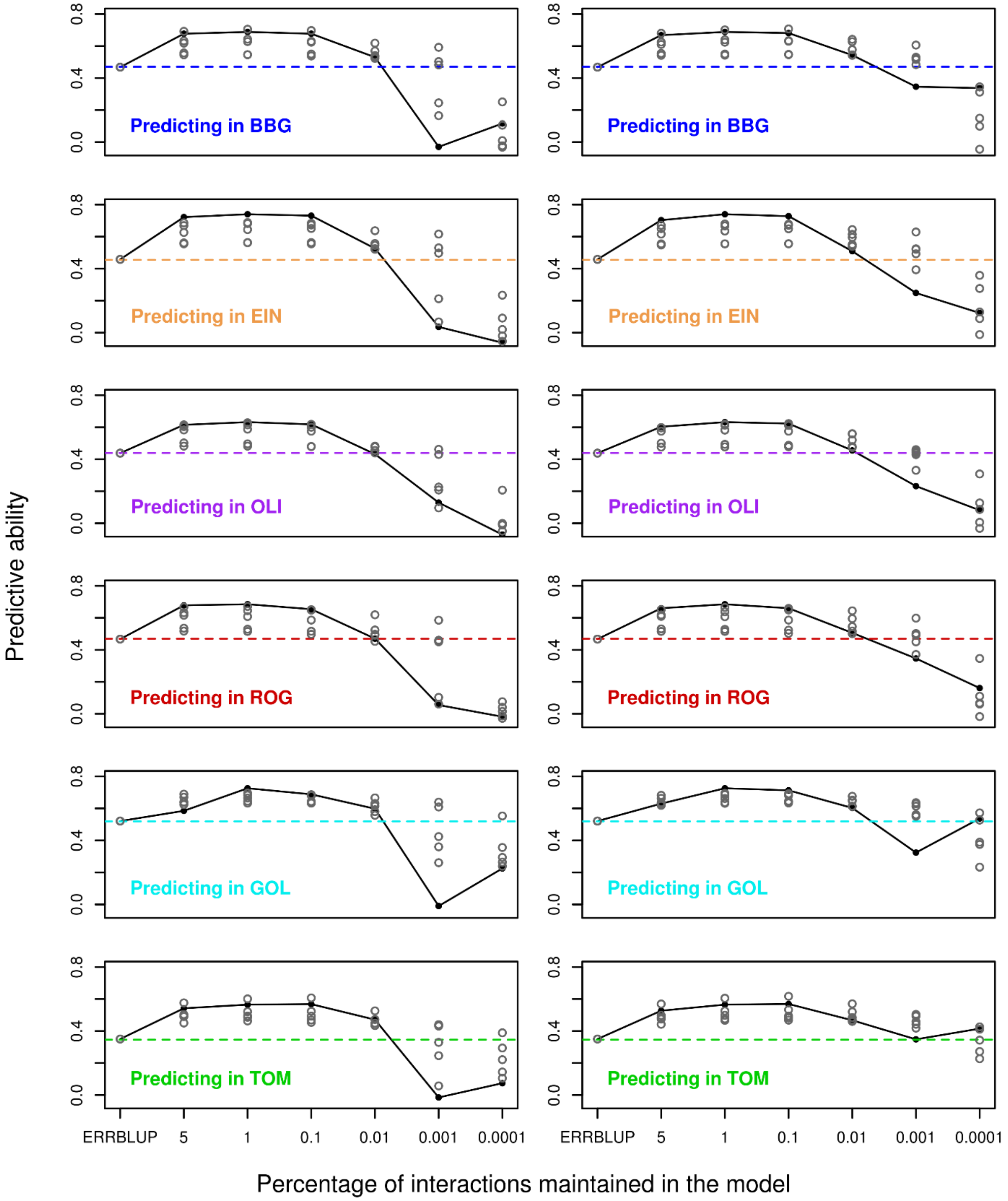

Fig. S16 Predictive ability for univariate GBLUP within environment (dashed horizontal line), univariate ERRBLUP within environment (gray open circle), univariate sERRBLUP using a single environment for selecting the SNP interactions (gray open circles) and univariate sERRBLUP using all 5 environments jointly (filled black circles and solid line) for the SNP interaction selection based on estimated effects sizes (left side) and estimated effects variances (right side) for trait PH-V4 in KE.

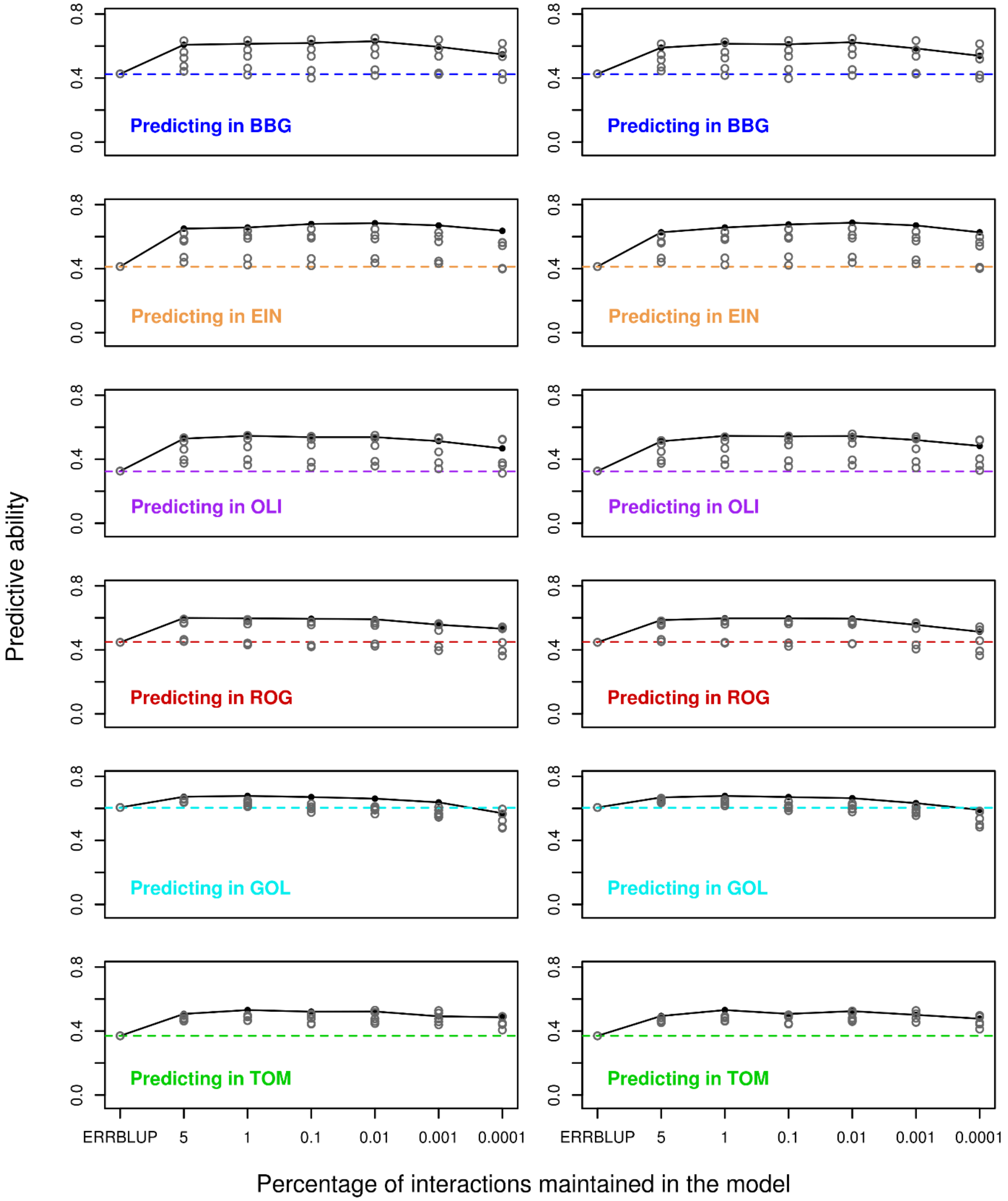

Fig. S17 Predictive ability for univariate GBLUP within environment (dashed horizontal line), univariate ERRBLUP within environment (gray open circle), univariate sERRBLUP using a single environment for selecting the SNP interactions (gray open circles) and univariate sERRBLUP using all 5 environments jointly (filled black circles and solid line) for the SNP interaction selection based on estimated effects sizes (left side) and estimated effects variances (right side) for trait PH-V4 in PE.

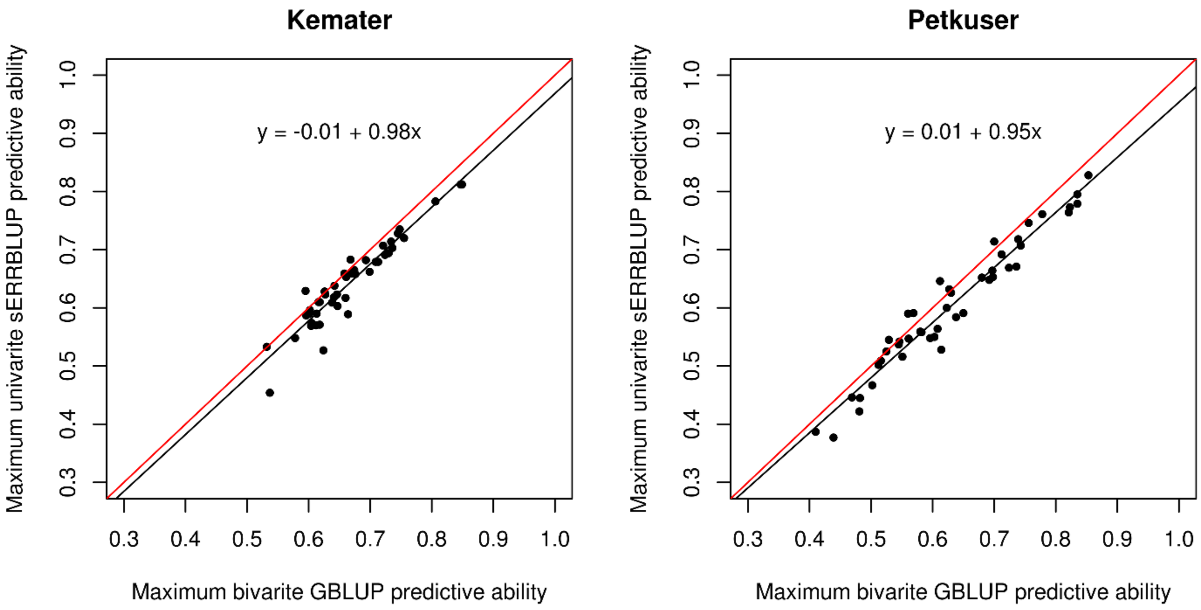

Fig. S18 The comparison between the maximum predictive abilities of bivariate GBLUP within all six environments and the maximum predictive abilities of univariate sERRBLUP across environments for all traits in KE (left side plot) and PE (right side plot). In each plot, the diagonal line (red line) and the overall linear regression line (black line) with the regression formula are shown.

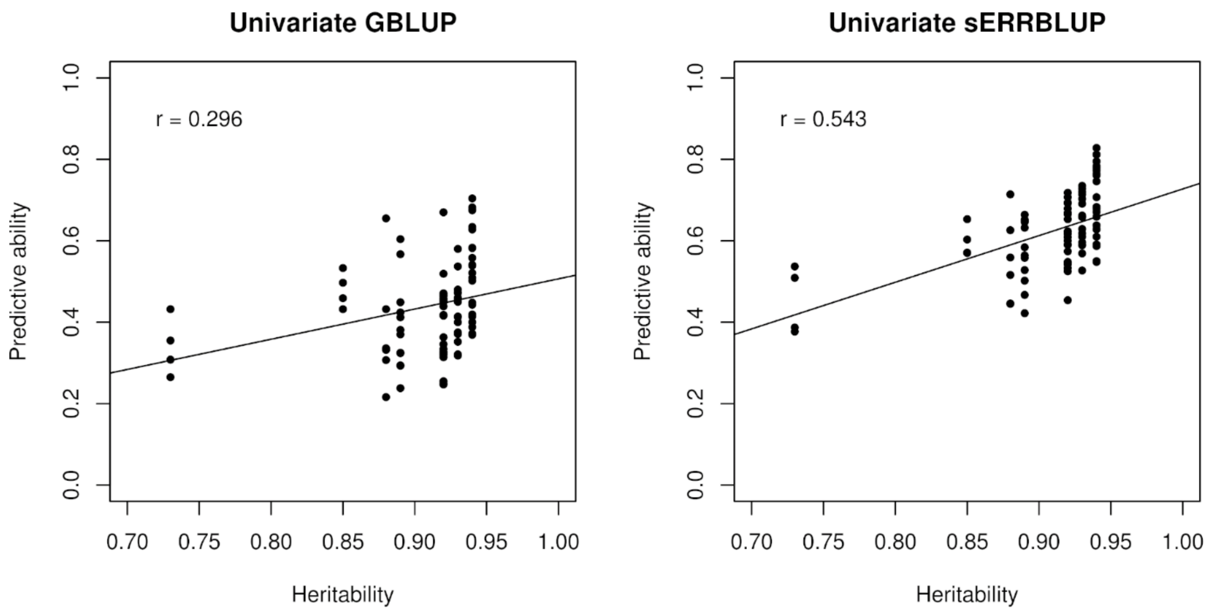

Fig. S19a The correlation between all eight traits' heritabilities and predictive abilities of univariate GBLUP within environments (left side) and maximum predictive abilities of univariate sERRBLUP across environments (right side) in both landraces. The black lines indicate the overall linear regression lines.

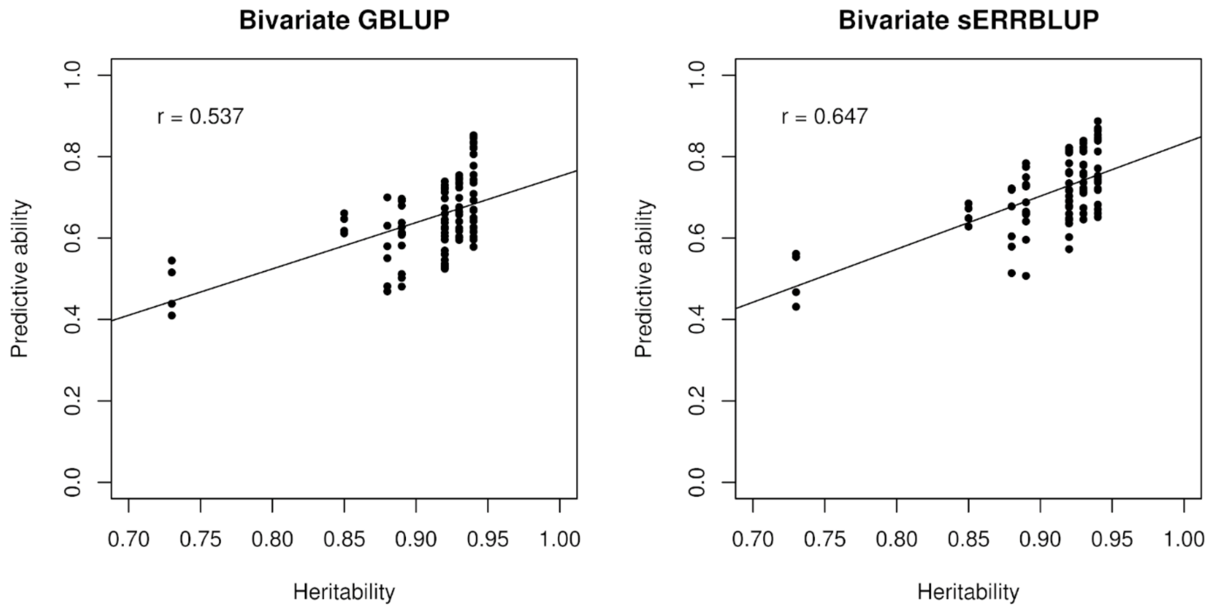

Fig. S19b The correlation between all eight traits' heritabilities and the maximum predictive abilities of bivariate GBLUP (left side) and maximum predictive abilities of bivariate sERRBLUP (right side) for all environments in both landraces. The black lines indicate the overall linear regression lines.
